# An *Orai1* gain-of-function tubular aggregate myopathy mouse model phenocopies key features of the human disease

**DOI:** 10.1038/s44318-024-00273-4

**Published:** 2024-10-17

**Authors:** Nan Zhao, Antonio Michelucci, Laura Pietrangelo, Sundeep Malik, Linda Groom, Jennifer Leigh, Thomas N O’Connor, Takahiro Takano, Paul D Kingsley, James Palis, Simona Boncompagni, Feliciano Protasi, Robert T Dirksen

**Affiliations:** 1 Department of Pharmacology and Physiology, University of Rochester Medical Center, Rochester, NY USA; 2 Department of Chemistry, Biology, and Biotechnology, University of Perugia, Perugia, Italy; 3 CAST, Center for Advanced Studies and Technology & DMSI, Department of Medicine and Aging Sciences, University Gabriele d’Annunzio of Chieti-Pescara, Chieti, Italy; 4 Department of Pediatrics, University of Rochester Medical Center, Rochester, NY USA; 5 CAST, Center for Advanced Studies and Technology & DNICS, Department of Neuroscience and Clinical Sciences, University Gabriele d’Annunzio of Chieti-Pescara, Chieti, Italy

**Keywords:** Muscle Disease, Calcium Signaling, ORAI1, Mitochondria, Proteomics, Genetics, Gene Therapy & Genetic Disease, Molecular Biology of Disease, Musculoskeletal System

## Abstract

Tubular aggregate myopathy (TAM) is a heritable myopathy primarily characterized by progressive muscle weakness, elevated levels of creatine kinase (CK), hypocalcemia, exercise intolerance, and the presence of tubular aggregates (TAs). Here, we generated a knock-in mouse model based on a human gain-of-function mutation which results in a severe, early-onset form of TAM, by inducing a glycine-to-serine point mutation in the ORAI1 pore (*Orai1*^*G100S/+*^ or GS mice). By 8 months of age, GS mice exhibited significant muscle weakness, exercise intolerance, elevated CK levels, hypocalcemia, and robust TA presence. Unexpectedly, constitutive Ca^2+^ entry in mutant mice was observed in muscle only during early development and was abolished in adult skeletal muscle, partly due to reduced ORAI1 expression. Consistent with proteomic results, significant mitochondrial damage and dysfunction was observed in skeletal muscle of GS mice. Thus, GS mice represent a powerful model for investigation of the pathophysiological mechanisms that underlie key TAM symptoms, as well as those compensatory responses that limit the damaging effects of uncontrolled ORAI1-mediated Ca^2+^ influx.

## Synopsis

Tubular aggregate myopathy (TAM) is caused by mutations in ORAI1 and STIM1 subunits of the Ca^2+^-release-activated Ca2+ (CRAC) channel. This study found that mice with a gain-of-function mutation in the ORAI1 channel pore that results in TAM in humans (Orai1^G100S/+^ mice) possess multiple hallmarks of the human disease.

- Orai1^G100S/+^ mice exhibit a robust presence of tubular aggregates at 8 months of age.
- Orai1^G100S/+^ mice exhibit several additional features of TAM including muscle weakness, elevated creatine kinase (CK), hypocalcemia, and exercise intolerance.
- Initial enhanced constitutive Ca^2+^ entry observed during early development is abolished in muscle fibers from Orai1^G100S/+^ adult mice.
- Proteomic, morphological, and functional analyses reveal alterations in mitochondrial structure and function in skeletal muscle of adult Orai1^G100S/+^ mice.

## Abstract (web summary)

The human mutation in the Orai1 calcium channel responsible for tubular aggregate myopathy is transferred to create a mouse model that offers insight into the underlying pathological etiology.

## Graphical abstract

**Figure:**
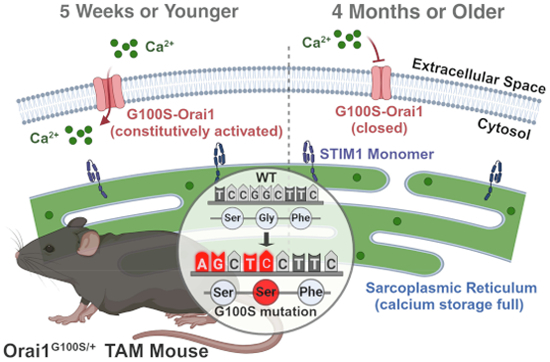

**Figure:**
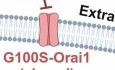

## Introduction

Calcium (Ca^2+^) is a universal second messenger that regulates a multitude of cellular processes including cell motility, gene transcription, exocytosis, and muscle contraction (Bagur and Hajnoczky, 2017; Feske et al, 2006; Zhang et al, 2005). As a crucial second messenger, intracellular Ca^2+^ is maintained at a low level ( ≤ 100 nM), while Ca^2+^ stored in the endoplasmic/sarcoplasmic reticulum (ER/SR) can be rapidly released to transiently increase cytosolic Ca^2+^ levels upon activation (Bagur and Hajnoczky, 2017). During sustained and/or repetitive Ca^2+^ release, ER/SR stores can become depleted, which activates store-operated Ca^2+^ entry (SOCE) to promote store refilling. SOCE is coordinated by STIM1 Ca^2+^ sensor proteins in the ER/SR membrane and Ca^2+^-permeable ORAI1 channels in the plasma membrane (Feske et al, 2006; Zhang et al, 2005). Upon store depletion, the unbinding of Ca^2+^ from STIM1 luminal EF hands results in STIM1 oligomerization and translocation to ER/SR membrane junctions where STIM1 oligomers are able to interact with and activate ORAI1 channels, thus promoting Ca^2+^ influx from the extracellular space (Hogan and Rao, 2007; Stathopulos et al, 2006).

Mutations in the SOCE machinery are associated with various human disorders. Recessive, loss-of-function mutations in STIM1 or ORAI1 result in reduced protein expression or disrupted STIM1-ORAI1 coupling, thus leading to insufficient SOCE and severe combined immunodeficiency (Feske et al, 2006; Lacruz and Feske, 2015; Picard et al, 2009). On the other hand, dominant gain-of-function (GoF) mutations in the STIM1 and ORAI1 proteins lead to enhanced SOCE function and constitutive Ca^2+^ entry in the absence of ER/SR store depletion (Lacruz and Feske, 2015; Morin et al, 2020) that results in tubular aggregate myopathy (TAM) (Bohm et al, 2017; Bohm et al, 2013; Endo et al, 2015; Garibaldi et al, 2017; Harris et al, 2017; Sallinger et al, 2020). TAM exhibits a wide range of disease severity with symptoms that primarily affect skeletal muscle, including progressive muscle weakness, elevated creatine kinase (CK) levels, hypocalcemia, exercise intolerance, as well as muscle cramping and myalgia (Chevessier et al, 2005). Additional multi-systemic manifestations, including thrombocytopenia and hyposplenism, are observed in some individuals with TAM (typically in individuals with mutations in STIM1, but not ORAI1), which reflects in a clinical continuum with Stormorken and York Platelet Syndrome (Bohm et al, 2017; Bohm et al, 2013; Endo et al, 2015; Garibaldi et al, 2017; Harris et al, 2017; Hedberg et al, 2014; Misceo et al, 2014; Stormorken et al, 1985; Walter et al, 2015). Despite variations in their symptoms, a consistent histological feature observed in muscle biopsies from all TAM patients is the presence of tubular aggregates (TAs) (Bohm et al, 2017; Bohm et al, 2013; Chevessier et al, 2005; Endo et al, 2015; Garibaldi et al, 2017; Harris et al, 2017). TAs are morphological abnormalities characterized by abnormal accumulations of highly ordered and tightly packed tubules within skeletal muscle fibers, appearing as honeycomb-like structures (Chevessier et al, 2005; Chevessier et al, 2004). Several studies indicate that TAs are formed by membranes of SR origin, though the precise molecular mechanisms that underlie their formation and growth are unclear (Bohm et al, 2017; Bohm et al, 2013; Chevessier et al, 2005; Endo et al, 2015; Harris et al, 2017; Salviati et al, 1985).

Four different STIM1 TAM mouse models were previously described that phenocopy the multi-systemic aspects of TAM (Cordero-Sanchez et al, 2020; Gamage et al, 2018; Grosse et al, 2007; Silva-Rojas et al, 2019). However, none of these STIM1 models of TAM exhibit TAs and no ORAI1 mouse models of TAM are reported in the literature. Here, we generated mice with a pore mutation in the mouse ORAI1 protein (glycine to serine mutation at amino acid 100 or G100S), analogous to the G98S mutation in ORAI1 found in humans with TAM. This mutation results in a severe, childhood onset form of TAM that has been identified in at least three unrelated TAM families (Bohm et al, 2017; Endo et al, 2015). Positioned within the first transmembrane region of ORAI1, residue G98 serves as the gating hinge in a rigid section of the ORAI1 pore, thus being critical for channel opening upon activation (Zhang et al, 2011). When mutated to a serine residue, the ORAI1 pore does not close properly, resulting in a constitutively activated ORAI1 channel that permits Ca^2+^ influx independent of Ca^2+^ store depletion and STIM1 oligomerization (Zhang et al, 2011). Patients with the G98S mutation in ORAI1 exhibit primarily skeletal muscle manifestations, including myopathy, exercise intolerance, and a robust presence of TAs upon electron microscopy (EM) analysis of muscle biopsies. Individuals with TAM also exhibit elevated serum CK levels, hypocalcemia, ichthyosis, and miosis (Bohm et al, 2017; Endo et al, 2015). While hematological phenotypes (anemia, bleeding disorders) are observed in some TAM individuals, they are not typically observed in individuals with the G98S mutation in ORAI1 (Bohm et al, 2017; Endo et al, 2015).

Here, we primarily focused on the skeletal muscle aspects of heterozygous *Orai1*^*G100S/+*^ (GS) mice, characterizing their muscle structure and function, as well as Ca^2+^ handling ability. GS mice exhibited muscle weakness, exercise intolerance, hypocalcemia, elevated CK levels, and a robust presence of TAs by 8 months (8 M) of age. Despite the well-documented GoF effect of the G100S mutation in ORAI1 (Bohm et al, 2017; Endo et al, 2015; Zhang et al, 2011), constitutive Ca^2+^ entry was only observed in muscle cells during early development (myotubes and muscle fibers up to 5 weeks of age), but was abolished after adulthood (4 M or older). Moreover, SOCE was significantly reduced across all ages, which was due in part to a significant reduction in ORAI1 protein expression. The postnatal ablation of constitutive Ca^2+^ entry and lifelong reduction in SOCE might represent an adaptation of muscle designed to limit excessive Ca^2+^-induced muscle damage and necrosis. In addition, morphological and functional studies using purified mitochondria and isolated *flexor digitorum brevis* (FDB) fibers from GS mice, as well as the proteomic analysis of GS *tibialis anterior* muscle, revealed substantial mitochondrial damage and dysfunction, suggesting a connection between ORAI1 activity and mitochondrial structure/function.

## Results

### Generation of *Orai1*^*G100S/+*^ mice

Knock-in *Orai1*^*G100S/+*^ (GS) mice were generated by the Mouse Genome Editing Resource Facility at University of Rochester Medical Center using a CRISPR/Cas9 gene editing approach (details provided in “Methods”) (Fig. EV1A,B). Heterozygous *Orai1* mice were backcrossed with wild-type (WT, *Orai1*^*+/+*^) mice of the same genetic background (C57Bl/6 J) for more than seven generations to establish the congenic line and select against potential off-target editing. Unexpectedly, we found that, while crossing female GS mice with male WT mice resulted in pregnancies (16 different successful crosses), the female GS mice committed parental infanticide after delivery where all pups were destroyed/eaten. However, no such issues were observed when WT female mice were bred with male GS mice. Thus, this breeding scheme (Fig. EV1C) was adopted for all experiments used in this study. Following 49 successful pregnancies using this breeding scheme, a total of 274 offspring were produced, with an average litter size of ~5.7 pups/litter. Among all pups, there were 146 WT mice and 135 GS mice (1.08:1), consistent with the expected Mendelian ratio (1:1) for this breeding scheme.

**Figure EV1:**

Generation of GS mouse model. (**A**) CRISPR-Cas9 strategy used to generate heterozygous GS knock-in mice. The G100S mutation is located at the end of exon 1. A donor template with desired glycine-to-serine mutation and altered PAM region was introduced to mouse embryos. (**B**) Sanger sequencing confirming heterozygous expression of the glycine to serine mutation at ORAI1 amino acid position 100. (**C**) Cartoon depicting the breeding strategy used in this study to generate WT and GS mice.

We also attempted to produce homozygous mutant mice (*Orai1*^*G100S/G100S*^) by intercrossing heterozygous male and female GS mice. However, in spite of nine confirmed pregnancies from these heterozygous crosses, no viable litters were realized (all pups were maternally cannibalized within 24 h). Therefore, embryos were surgically removed at embryonic day 14.5–16.5 and genotyped from 5 additional pairs of confirmed heterozygous crossed pregnancies. From a total of 35 embryos genotyped from these 5 breeding pairs, 13 WT mice, 22 GS mice, and 0 *Orai1*^*G100S/G100S*^ mice were identified (1:1.7:0). This ratio deviates significantly from the expected Mendelian ratio of 1:2:1 (chi‐square test: *P* = 0.0104), consistent with a likely early embryonic lethality of Orai1^*G100S/G100S*^ homozygosity.

### Phenotypic characterization of GS mice

**Figure 1:**
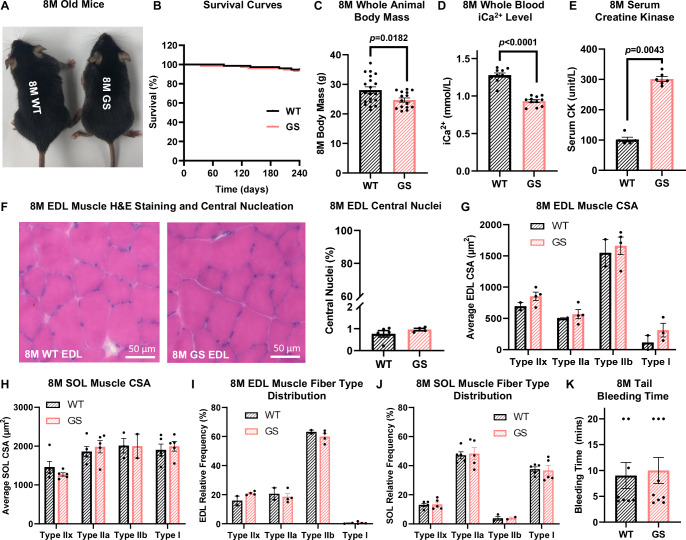
Phenotypic characterization of 8 M old GS mice. (**A**) GS mice showed a similar physical appearance as WT mice at 8 M of age. (**B**) Kaplan–Meier survival curves of WT and GS mice from a total of 76 8 M old WT mice and 73 8 M old GS mice. (**C**) Average ( ±SEM) body mass of 8 M old WT (*n* = 20) and GS (*n* = 15) mice. Significance was calculated using Student’s *t* test. The exact *P* value is labeled in the figure when *P* < 0.05. (**D**) Average ( ± SEM) whole blood iCa^2+^ level of 8 M old WT (*n* = 10) and GS (*n* = 11) mice. Significance was calculated using Student’s *t* test. The exact *P* value is labeled in the figure when *P* < 0.05. (**E**) Average ( ± SEM) serum CK level in 8 M old WT (*n* = 5) and GS (*n* = 6) mice. Significance was calculated using Mann–Whitney test. The exact *P* value is labeled in the figure when *P* < 0.05. (**F**) Representative H&E staining images of EDL muscle cross sections showed a similar percentage of central nucleation between 8 M WT and GS mice. Average ( ± SEM) percentage of fibers with central nuclei was quantified from the position of nuclei in 300 muscle fibers/genotype. (**G**, **H**) Average ( ± SEM) cross-sectional area (CSA) of different muscle fiber types in EDL (**G**) and SOL (**H**) muscles of 8 M old WT and GS mice. For EDL muscle, *n* = 2 and 4 for WT and GS group, respectively. For SOL muscle, *n* = 5 for both WT and GS group. (**I**, **J**) Average ( ± SEM) fiber type distribution of EDL (**I**) and SOL (**J**) muscles of 8 M WT and GS mice. For SOL muscle, *n* = 2 and 4 for WT and GS group, respectively. For EDL muscle, *n* = 5 for both WT and GS group. (**K**) Average ( ± SEM) tail bleeding time in 8 M WT (*n* = 8) and GS (*n* = 9) mice. Source data: available online for (**B**–**K**).

8 M heterozygous GS mice showed no overt physical abnormalities and the Kaplan–Meier survival analyses revealed no signs of early mortality (Fig. 1A,B). Monitoring the body mass of GS mice over 12 months revealed a modest, but significant reduction at 8 M and 12 M of age (Figs. 1C and EV2A). However, *extensor digitorum longus* (EDL) muscle mass from GS mice was not significantly different at any age assessed (4, 8 and 12 M) and *soleus* (SOL) muscle mass was equivalent between 8 M old WT and GS mice (Fig. EV2B,C).

**Figure EV2:**

Additional phenotypic characterization of GS mice. (**A**) Compared to age-matched WT mice, GS mice showed no significant differences in average body mass at 4 M of age, but significantly reduced body mass at 12 M of age. For 4 M old mice, *n* = 11 and 10 for WT and GS mice, respectively. For 12 M old mice, *n* = 9 for WT mice and *n* = 9 for GS mice. Significance was calculated using Student’s *t* test. The exact *P* value is labeled in the figure when *P* < 0.05. (**B**) GS mice showed similar EDL muscle mass at 4, 8 and 12 M of age as WT mice. For 4 M old mice, *n* = 11 and 10 for WT and GS mice, respectively. For 8 M old mice, *n* = 10 and 9 for WT and GS mice, respectively. For 12 M old mice, *n* = 7 and 8 for WT and GS mice, respectively. (**C**) WT (*n* = 7) and GS (*n* = 7) mice exhibited similar SOL muscle mass at 8 M of age. (**D**) Whole blood analysis of Na^+^, K^+^, glucose, and pH from 8 M old WT (*n* = 10) and GS (*n* = 11) mice. (**E**, **F**) Representative fiber type images of EDL (**E**) and SOL (**F**) muscles from 8 M old WT and GS mice.

In line with *ORAI1*^*G98S/+*^ TAM patients who frequently exhibit hypocalcemia and elevated serum CK levels (Bohm et al, 2017; Endo et al, 2015), an average of 27% reduction in whole blood ionized calcium (iCa^2+^) was observed in 8 M GS mice, while all other blood parameters (including sodium [Na^+^], potassium [K^+^], glucose, and pH) remain unaltered (Figs. 1D and EV2D). Similarly, serum CK levels in 8 M GS mice were increased ~3-fold compared to that of age-matched WT mice (Fig. 1E), consistent with a low level of muscle damage observed in TAM patients. Hematoxylin and Eosin (H&E) staining of EDL muscle from 8 M GS mice revealed an absence of significant dystrophic features, muscle regeneration, and central nuclei (Fig. 1F). Cross-sectional analysis of EDL and SOL muscles revealed normal fiber size and fiber type distribution in 8 M old GS mice (Figs.1G–J and EV2E,F).

Individuals with TAM resulting from the G98S mutation in human ORAI1 do not typically exhibit hematological problems. However, since thrombocytopenia and anemia are observed in some TAM patients caused by STIM1 mutations (Morin et al, 2020), as well as three different STIM1 TAM mouse models (Cordero-Sanchez et al, 2020; Grosse et al, 2007; Silva-Rojas et al, 2019), we conducted blood cell analyses and assessed clotting function in 8 M old WT and GS mice. Complete blood count (CBC) analysis revealed that blood platelet counts, mean platelet volume (MPV), red blood cell number, hemoglobin (HGB), hematocrit, and white blood cells (including lymphocytes, monocytes, and granulocytes) were all not significantly altered between 8 M old WT and GS mice (Appendix Fig. S1). Consistent with these findings and most clinical observations of *ORAI1*^*G98S/+*^ TAM patients (Bohm et al, 2017; Endo et al, 2015), GS mice exhibited normal hemostasis with no change in blood clotting rate (Fig. 1K).

### Tubular aggregates in GS mice

**Figure 2:**
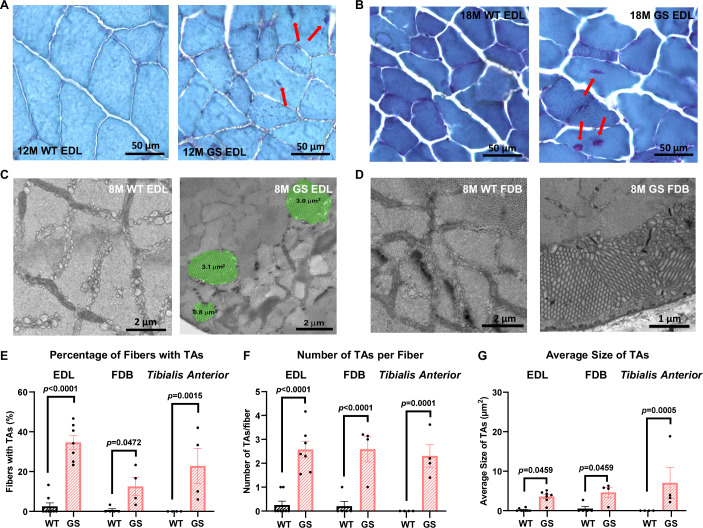
Tubular aggregates in fast-twitch muscles of GS mice. (**A**, **B**) Representative Gomori trichrome images of EDL muscles from 12 M (**A**) and 18 M (**B**) old WT (left) and GS (right) mice. TAs are visualized as pink/purple inclusions as indicated by red arrows. (**C**, **D**) Representative EM images of cross sections of EDL (**C**) and FDB (**D**) muscles from 8 M old WT (left) and GS (right) mice. Images were taken from different muscle fibers. TAs (colored in green) for the GS EDL fiber shown in (**C**) exhibit a honeycomb-like structure. (**E**–**G**) Average (±SEM) percentage of fibers with TAs (**E**), number of TAs/fiber (**F**), and TA size (**G**) in EDL (left), FDB (middle), and *tibialis anterior* (right) muscles of 8 M old WT and GS mice. *n* = 8, 5, 4 for WT mice and *n* = 7, 4, 4 for GS mice in sequential order. Significance was calculated using two-way ANOVA followed by Holm–Sidak’s multiple comparisons post hoc test. The exact *P* value is labeled in the figure when adjusted *P* < 0.05. Source data: available online for (**A**–**G**).

The presence of TAs in muscle biopsies from TAM patients is a key diagnostic histological hallmark of the disease (Chevessier et al, 2005; Chevessier et al, 2004). Consistent with that observed in muscle biopsies from TAM patients (Bohm et al, 2017; Endo et al, 2015), Gomori trichrome staining where TAs appear as pink/purple inclusions confirmed the presence of TAs (red arrows) in EDL muscle sections from 12 M and 18 M old GS mice, but not in corresponding muscles from representative age-matched WT mice (Fig. 2A,B). These findings were corroborated by quantitative EM measurements of honeycomb-like TA structures in fast-twitch EDL and FDB muscles of 8 M old GS mice (but not in slow-twitch muscles, like SOL) (Fig. 2C,D). Compared to 8 M old WT mice, where only a single TA was occasionally observed within a single fiber, TAs were detected in ~30% of EDL fibers, ~10% of FDB fibers, and ~20% of *tibialis anterior* fibers from 8 M old GS mice (Fig. 2E). In addition, muscles from 8 M GS mice exhibited significant increases in both the average number of TAs/fiber (Fig. 2F) and TA size (Fig. 2G). Previous studies found that TAs naturally occur in skeletal muscle of aged (e.g. 24 M) male (but not female) WT mice (Boncompagni et al, 2012). However, EDL muscles from both 8 M old male and female GS mice exhibited increases in TA incidence, number/fiber, and size (Fig. EV3A–C), suggesting no sex variation. Moreover, while TAs in EDL muscle of GS mice were not observed at 4 months of age, they were significantly observed with higher incidence and number/fiber at both 8 M and 12 M of age (Fig. EV3D–F), demonstrating an age dependence of TA formation in EDL muscle of GS mice.

**Figure EV3:**

Sex- and age-dependent analyses of tubular aggregates in EDL muscle from 8 M WT and GS mice. (**A**–**C**) Average ( ± SEM) percentage of fibers with TAs (**A**), number of TAs/fiber (**B**), and TA size (**C**) in EDL muscles from 8 M old male and female WT and GS mice. For male group, *n* = 6 and 4 for WT and GS group, respectively. For female mice, *n* = 4 for WT mice and *n* = 4 GS mice. Significance was calculated using 2-way ANOVA followed by Holm–Sidak’s multiple comparisons post hoc test. The exact *P* value is labeled in the figure when adjusted *P* < 0.05. (**D-F**) Average ( ± SEM) percentage of fibers with TAs (**D**), number of TAs/fiber (**E**), and TA size (**F**) in EDL muscles from 4 M, 8 M, and 12 M old WT and GS mice. For 4 M and 12 M mice, *n* = 4 for WT mice and *n* = 4 GS mice. For 8 M old mice, *n* = 8 and 7 for WT and GS mice, respectively. Significance was calculated using 2-way ANOVA followed by Holm–Sidak’s multiple comparisons post hoc test. The exact *P* value is labeled in the figure when adjusted *P* < 0.05.

### GS mice exhibit muscle weakness and exercise intolerance

**Figure 3:**
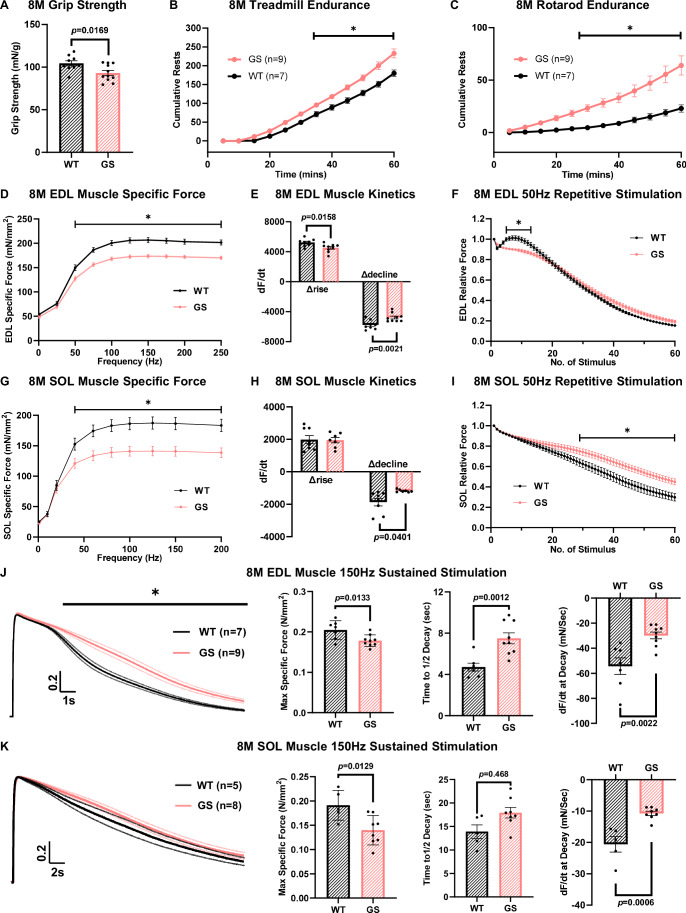
Increased muscle weakness and exercise intolerance of 8 M old GS mice. (**A**) In vivo behavioral studies of average ( ± SEM) normalized grip strength in 8 M old WT (*n* = 9) and GS mice (*n* = 11). Significance was calculated using Student’s *t* test. The exact *P* value is labeled in the figure when *P* < 0.05. (**B**, **C**) In vivo behavioral studies of treadmill endurance (B) and rotarod endurance (**C**) tests in 8 M old WT (*n* = 7) and GS (*n* = 9) mice where averaged ( ± SEM) cumulative rest/fall were plotted. Significance was calculated using multiple *t* test. * indicates adjusted *P* < 0.05 (adjusted *P* = 0.0316, 0.0088, 0.0006, 0.0001, <0.0001, <0.0001 in sequential order for (**B**) and adjusted *P* = 0.0114, 0.0025, 0.0005, <0.0001, <0.0001, <0.0001 in sequential order for **C**). (**D–F**) Average ( ± SEM) specific force-frequency curves (**D**), maximum rates of force production (Δrise) and relaxation (Δdecline) during stimulation at 150 Hz (**E**), and peak specific force production during repetitive, high frequency (50 Hz, 500 ms, every 2.5 s) stimulation (**F**) in EDL muscles from 8 M old WT (*n* = 9) and GS (*n* = 9) mice. Significance was calculated using either multiple *t* test (for line graphs) or two-way ANOVA followed by Holm–Sidak’s multiple comparisons post hoc test (for the bar graph). For the bar graph, the exact *P* value is labeled in the figure when *P* < 0.05. For the line graphs, * indicates adjusted *P* < 0.05 (for **D**, all adjusted *P* < 0.0001, except at 50 Hz, the adjusted *P* = 0.0015; for (**F**), adjusted *P* = 0.0216, 0.0028, 0.0008, 0.0012, 0.0033, 0.0149 in sequential order, starting from the 5th stimulation.). (**G**–**I**) Average ( ± SEM) specific force-frequency curves (**G**), maximum rates of force production (Δrise) and relaxation (Δdecline) during stimulation at 150 Hz (**H**), and peak specific force production during repetitive, high frequency (50 Hz, 500 ms, every 2.5 s) stimulation (**I**) in SOL muscles from 8 M old WT (*n* = 8) and GS (*n* = 7) mice. Significance was calculated using either multiple *t* test (for line graphs) or two-way ANOVA followed by Holm–Sidak’s multiple comparisons post hoc test (for the bar graph). For the bar graph, the exact *P* value is labeled in the figure when *P* < 0.05. For the line graphs, * indicates adjusted *P* < 0.05 (for **G**, adjusted *P* = 0.0279, 0.0031, 0.0014, 0.0013, 0.0012, 0.0012, 0.0012 in sequential order; for (**I**), adjusted *P* = 0.0465–0.0089, starting from the 36th stimulation.). (**J**, **K**) Normalized average ( ± SEM) specific force traces elicited during sustained 150 Hz stimulation (left), peak specific force (middle left), time to 50% relaxation (middle right), and maximum rate of contractile decay (right) of EDL (**J**) and SOL (**K**) muscles from 8 M old WT and GS mice. For EDL muscle, *n* = 7 and 9 for WT and GS groups, respectively. For SOL muscle, *n* = 5 and 8 for WT and GS groups, respectively. Significance was calculated using either multiple *t* test (for line graphs) or Student’s *t* test (for bar graphs). For the bar graphs, the exact *P* value is labeled in the figure when *P* < 0.05. For the line graphs, * indicates adjusted *P* < 0.05. Source data: available online for (**A**–**K**).

We next assessed in vivo muscle strength and exercise tolerability of GS mice in a series of behavioral studies. Grip strength normalized to body mass was significantly reduced in 8 M old GS mice compared to age-matched WT mice (104.4 ± 3.0 mN/g and 93.1 ± 3.0 mN/g for WT and GS mice, respectively) (Fig. 3A). Mice were also assessed for exercise tolerance using both treadmill and rotarod endurance tests. Compared to their WT counterparts, 8 M old GS mice rested significantly more often during a 1-hour treadmill endurance task (Fig. 3B) and fell more frequently during the 1-hour rotarod endurance task (Fig. 3C). Specifically, by the end of these tasks, GS mice rested on the treadmill 23% more often than WT mice and fell from the rotarod nearly 3× more than WT mice (64.0 ± 9.1 falls vs 22.9 ± 3.5 falls for GS and WT mice, respectively).

We also assessed ex vivo muscle contractile function of EDL (Fig. 3D–F) and SOL (Fig. 3G–I) muscles from 8 M old WT and GS mice. Both muscles showed significant reductions in specific force production (Fig. 3D,G) under high stimulation frequencies (≥50 Hz/40 Hz for EDL/SOL muscles, respectively), as well as a modest reduction in the maximum rate of force relaxation (-dF/dt) during tetanic stimulation (150 Hz) (Fig. 3E,H, right). In addition, EDL muscles of GS mice also exhibited a significant decrease in the maximum rate of force generation during tetanic stimulation (dF/dt) (Fig. 3E, left). EDL and SOL muscles were also stimulated with 60 trains of repetitive 50 Hz stimulations (500 ms, every 2.5 s, 0.2 duty cycle). Similar to previous studies (Michelucci et al, 2019; Michelucci et al, 2020), EDL muscles from 8 M WT mice exhibited a modest increase in specific force for several stimulus trains after the second stimulus, which correlates with increased SOCE activity, followed by a steady, time-dependent decline in force production (Fig. 3F, black trace). However, this enhancement of force production during repetitive stimulation was significantly reduced in EDL muscles isolated from GS mice (that lack SOCE activity at this age) (Fig. 3F, pink trace). For SOL muscles from GS mice, peak specific force during the second half of the repetitive stimulation protocol was significantly increased compared to that of SOL muscle from age-matched WT mice (Fig. 3I), suggesting that SOL muscles from GS mice are even more resistant to exercise-induced fatigue. These findings suggest that compensatory changes in SOL muscles of GS mice limit exercise intolerance, which could partially explain the absence of TAs in SOL muscles.

Of note, the overall rate of force decline after ~20 repetitive stimuli was somewhat reduced in both EDL and SOL muscles from GS mice (Fig. 3F,I). Thus, we also evaluated the rate of force decay during a prolonged tetanic (150 Hz) stimulation (15 s for EDL muscle and 30 s for SOL muscle). Compared to age-matched WT mice, both EDL and SOL muscles from 8 M old GS mice exhibited significantly reduced peak specific forces, prolonged time for 50% force decay, and decreased maximum rate of force decay (Fig. 3J,K). A similar reduction in the rate of force decay during sustained, tetanic stimulation was also reported for* tibialis anterior* muscles from *Stim1*^*R304W/+*^ TAM mice (Silva-Rojas et al, 2019).

### Postnatal abolishment of constitutive Ca^2+^ entry and reduced SOCE activity

Prior studies using myotubes derived from TAM patients, as well as transfected HEK293 cells, demonstrated that the G98S TAM mutation in human ORAI1 produces a GoF effect that results in STIM1-independent ORAI1 channel opening, such that ORAI1 channels are activated even in the absence of store depletion. As a result, the G98S mutation in ORAI1 leads to constitutive Ca^2+^ entry and enhanced SOCE activity (Bohm et al, 2017; Endo et al, 2015; Zhang et al, 2011).

**Figure 4:**
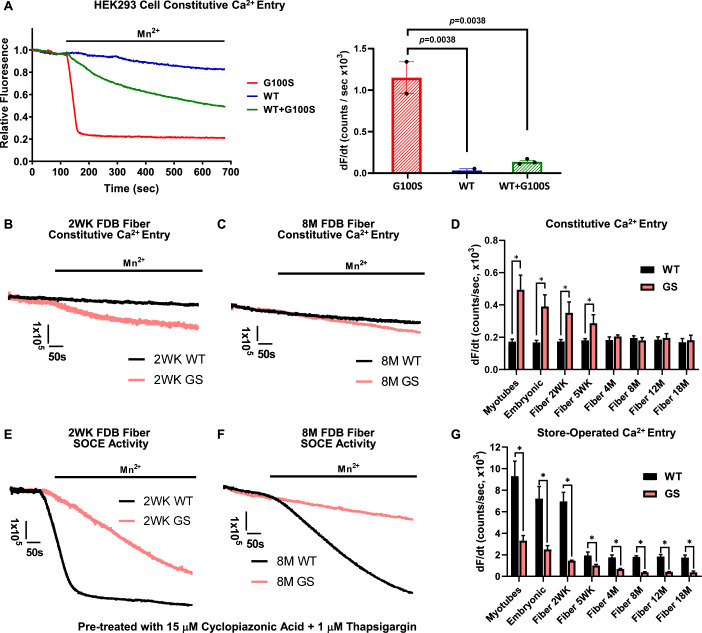
Constitutive and store-operated Ca^2+^ entry. (**A**) Representative Mn^2+^ quench of fura-2 fluorescence traces (left) in the absence of store depletion in naive HEK293 cells transfected with cDNA for WT mORAI1  (WT, blue, *n* = 2), G100S mORAI1 *Orai1* (G100S, red, *n* = 2), or a 1:1 ratio of WT and G100S mORAI1 (WT + G100S, green, *n* = 2). Average ( ± SEM) maximum rate of Mn^2+^ quench (right) in the absence of store depletion for HEK293 cells expression WT (blue), G100S (red), or a 1:1 ratio of WT and G100S (green) mORAI1. Significance was calculated using 1-way ANOVA followed by Holm–Sidak’s multiple comparisons post hoc test. The exact *P* value is labeled in the figure when adjusted *P* < 0.05. (**B**, **C**) Representative traces of Mn^2+^ quench of fura-2 fluorescence in the absence of store depletion (constitutive Ca^2+^ entry) in FDB fibers isolated from 2 WK (**B**) and 8 M (**C**) old WT (black) and GS (pink) mice. (**D**) Average ( ± SEM) maximum rate of Mn^2+^ quench in the absence of store depletion (constitutive Ca^2+^ entry) in myotubes and myofibers from WT (black) and GS (pink) mice. *n* = 3, 3, 4, 5, 6, 6, 6, 5 for WT mice and *n* = 4, 4, 4, 5, 6, 6, 6, 5 for GS mice at the given ages in sequential order. Significance was calculated using two-way ANOVA followed by Holm–Sidak’s multiple comparisons post hoc test. * indicates adjusted *P* < 0.05 where all adjusted *P* < 0.0001 except ‘Fiber 5 WK’ group whose *P* = 0.0001. (**E**, **F**) Representative traces of Mn^2+^ quench of fura-2 fluorescence following store depletion (SOCE) in FDB fibers isolated from 2 WK (**E**) and 8 M (**F**) old WT (black) and GS (pink) mice. (**G**) Average (± SEM) maximum rate of Mn^2+^ quench following store depletion (SOCE) in myotubes and myofibers from WT (black) and GS (pink) mice. *n* = 3, 3, 4, 5, 6, 6, 6, 5 for WT mice and *n* = 4, 4, 4, 5, 6, 6, 6, 5 for GS mice at the given ages in sequential order. Significance was calculated using two-way ANOVA followed by Holm–Sidak’s multiple comparisons post hoc test. * indicates adjusted *P* < 0.05 where all adjusted *P* < 0.0001 except ‘Fiber 5 WK’ group whose *P* = 0.0012. Source data: available online for (**A**–**G**).

To quantify the effect of the G100S mutation in mouse ORAI1 on constitutive Ca^2+^ entry through ORAI1 channels, we measured the maximum rate of Mn^2+^ quench of fura-2 fluorescence upon the addition of 0.5 mM Mn^2+^ to HEK293 cells transfected with cDNA encoding either WT mouse *Orai1*  (mORAI1), G100S mORAI1, or WT and G100S mORAI1 transfected at 1:1 ratio. Consistent with what was reported previously for human G98S *ORAI1*, HEK293 cells expressing G100S mORAI1 alone exhibited an immediate and remarkedly high rate of Mn^2+^ quench even in the absence of prior Ca^2+^ store depletion (Fig. 4A). When HEK293 cells were co-transfected with both WT and G100S mORAI1 at a 1:1 ratio, the maximum rate of Mn^2+^ quench was intermediate to when WT mORAI1 or G100S mORAI1 were expressed alone (Fig. 4A), consistent with a dominant GoF effect of the G100S mutation on heteromeric ORAI1 channels. In agreement with this GS GoF effect on constitutive Ca^2+^ entry, GS myotubes, embryonic myofibers and FDB fibers isolated from 2 to 5 week (WK) old GS mice all exhibited significantly elevated Mn^2+^ quench rate in the absence of prior Ca^2+^ store depletion (Fig. 4B,D). However, unexpectedly, we found that this increase in constitutive Ca^2+^ entry was absent in FDB fibers isolated from GS mice at all ages after muscle development (4, 8, 12, and 18 M) (Fig. 4C,D).

We also quantified the magnitude of store-operated Ca^2+^ entry (SOCE) in WT and GS mice across all ages (myotubes to 18 M of age) using a similar Mn^2+^ fura-2 quench approach, except that in this case, fibers were pretreated with a cocktail of SERCA pump inhibitors that fully depleted SR Ca^2+^ stores. Compared to that of myotubes/fibers from age-matched WT mice, maximal rate of Mn^2+^ quench following SR Ca^2+^ store depletion was significantly reduced in myotubes/fibers from GS mice across all developmental and adult ages (Fig. 4E–G). While the maximal Mn^2+^ quench rate following store depletion was ~1.8 × 10^3^ counts/s in FDB fibers from WT mice at all ages after 5 WK, this rate in FDB fibers from GS mice decreased steadily from 1.0 ± 0.10 × 10^3^ counts/s at 5 WK of age to only 0.4 ± 0.12 × 10^3^ counts/s at 18 M of age (Fig. 4G).

### Reduced ORAI1 expression level in GS mice

**Figure 5:**
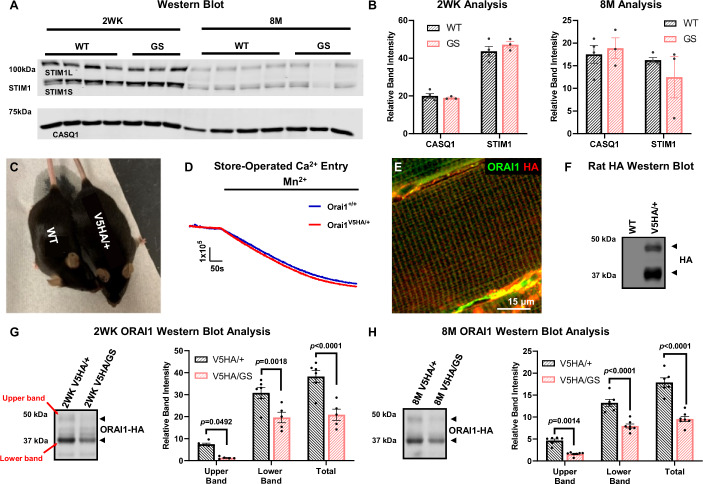
CASQ, STIM1, and ORAI1 protein expression. (**A**) Representative western blots of STIM1L and STIM1S (upper blot) and CASQ1 (lower blot) in *tibialis anterior* muscles from 2 WK (left) and 8 M old (right) WT (*n* = 4) and GS (*n* = 3) mice. (**B**) Average ( ± SEM) CASQ1 and STIM1 expression in *tibialis anterior* muscles from 2 WK (left) and 8 M (right) old WT (*n* = 4) and GS (*n* = 3) mice. STIM1 expression was measured as the sum of STIM1S and STIM1L. Protein expression levels were normalized to Ponceau S. (**C**–**E**) Knock-in mice with a V5-3xHA epitope added to the extreme ORAI1 C-terminus *(Orai1*^*V5HA/+*^ or V5HA mice) exhibit similar physical appearance (**C**), SOCE activity (**D**), and ORAI1 subcellular localization (**E**) as age-matched WT mice. (**F**) Representative western blot of *quadricep* muscle lysates from WT and V5HA/+ mice probed with rat HA antibody. (**G**, **H**) Representative western blots (left) and average ( ± SEM) bar graphs (right) for ORAI1 expression probed with rat HA antibody in *tibialis anterio*r muscles from 2 WK (**G**) and 8 M (**H**) old V5HA/+ and V5HA/GS mice. For 2 WK condition, *n* = 6 and 5 for WT and GS group, respectively. For 8 M condition, *n* = 6 and 7 for WT and GS group, respectively. ORAI1 expression level was measured as the sum of upper and lower bands. Protein expression levels were normalized to Ponceau S. Significance was calculated using two-way ANOVA followed by Holm–Sidak’s multiple comparisons post hoc test. The exact *P* value is labeled in the figure when adjusted *P* < 0.05. Source data: available online for (**A**, **B**, **D**–**H**).

We next examined whether the unexpected reduction in SOCE activity of GS muscle fibers is resulted from reduced expression of proteins that control SR Ca^2+^ store content (calsequestrin-1, CASQ1) and SOCE activation (STIM1 and ORAI1). Western blot analyses of STIM1 and CASQ1 showed no significant difference between genotypes at either 2 WK or 8 M of age (Fig. 5A,B).

**Figure EV4:**
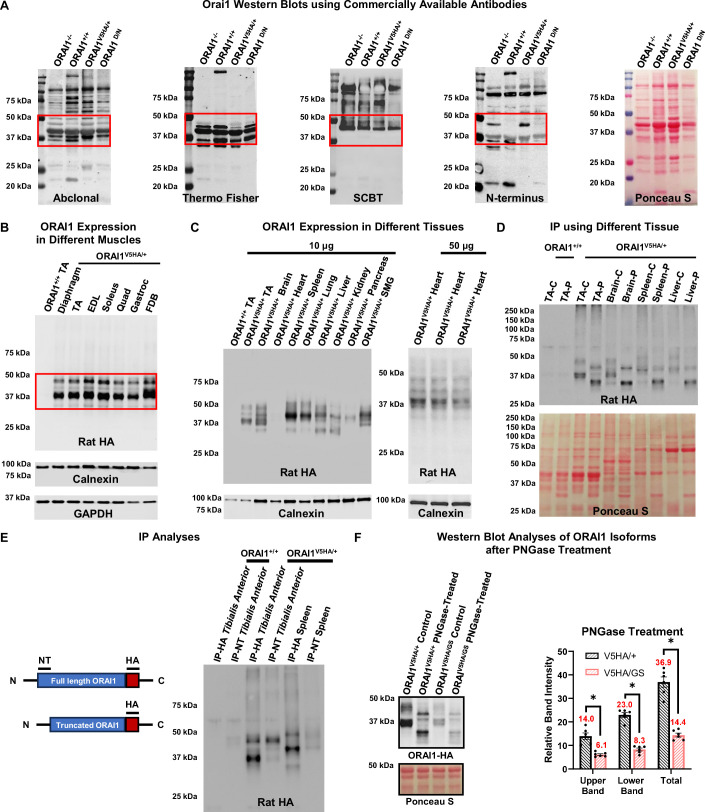
Western blots analyses using commercially available ORAI1 and HA antibodies in various tissues. (**A**) Representative western blots of muscle lysates from WT mice (ORAI1^+/+^), constitutive muscle-specific ORAI1 KO mice (ORAI1^-/-^), heterozygous V5HA/+ mice (ORAI1^V5HA/+^), and muscle-specific dominant negative ORAI1 transgenic mice (ORAI1^D/N^) probed with commercially available ORAI1 antibodies from Abclonal, ThermoFisher, Santa Cruz Biotechnology (SCBT), and a rabbit polyclonal antibody raised against the first 20 amino acids of the ORAI1 N-terminus. The final gel (right) is the Ponceau S stained gel used for protein loading. No specific ORAI1 band at the expected molecular weight (and absent in the ORAI1 KO lane) was observed for any of these antibodies. (**B**) Representative western blot of endogenous ORAI1 expression probed with rat HA primary antibody across multiple different skeletal muscles (diaphragm, *tibialis anterior* (TA), EDL, SOL, *quadriceps* (Quad), *gastrocnemius* (Gastroc), and FDB) obtained from V5HA/+ mice. The first lane is a *tibialis anterior*(TA) muscle lysate from a WT (*Orai1*^*+/+*^) mouse used as a negative control. Calnexin and GAPDH loading control blots are shown below the rat HA blot. Two different Orai1-V5HA-tagged isoforms are observed in each muscle. (**C**) Representative western blot of endogenous ORAI1 expression probed with rat HA primary antibody across multiple different organs (*tibialis anterior* (TA), brain, heart, spleen, lung, liver, kidney, pancreas, and submandibular gland (SMG)) obtained from V5HA/+ mice. 10 µg (left) or 50 µg (right) lysate was loaded in each lane. Calnexin loading control blots are shown below the rat HA blots. A *tibialis anterior* muscle lysate from a control WT mouse (*Orai1*^*+/+*^) was used as a negative control. Note: Low level ORAI1 protein expression in the heart is best observed when loading 50 µg of lysate. Two distinct Orai1-V5HA-tagged isoforms are observed, though at somewhat different levels of glycosylation. (**D**) Representative rat HA western blot (upper) of skeletal muscle (*tibialis anterior* (TA)), brain, spleen, and liver lysates from *Orai1*^*V5HA/+*^ mice without (C) and after treatment with PNGase (P). A skeletal muscle (*tibialis anterior* (TA)) lysate from a control WT mouse (*ORAI1*^*+/+*^) was used as a negative control. The lower blot shows protein loading using Ponceau S stain. (**E**) Schematic of full-length and N-terminal truncated V5-3xHA-tagged ORAI1 isoforms (left) and a representative western blot of *tibialis anterior* (TA) immunoprecipitation (IP) using either a rat HA antibody (left lane pairs) or the rabbit polyclonal antibody raised against the first 20 amino acids of the mouse ORAI1 N-terminus (right lane pairs) and then blotted with rat HA primary antibody. While both ORAI1 isoforms are readily pulled-down with the rat HA antibody, the larger full-length ORAI1 isoform is preferentially pulled-down by the N-terminal antibody that recognizes the full-length isoform. These results are consistent with the smaller isoform reflecting an alternative N-terminal translation initiation as reported previously (Desai et al, 2015; Fukushima et al, 2012). (**F**) Representative HA western blot (left) of *tibialis anterior* muscle in the absence (control) and after treatment with PNGase (PNGase-treated) of skeletal muscle (*tibialis anterior*) from either a control V5HA/+ mouse or a V5HA/GS mouse. The lower blot shows total protein loading using Ponceau S stain. Average ( ± SEM) bar plot of relative HA-reactive upper, lower, and total (upper+lower) band intensity after PNGase treatment for muscle lysates from V5HA/+ (*black*, *n* = 6) and V5HA/GS mice (*pink*, *n* = 6) (right). Protein expression levels were normalized to Ponceau S. Mean relative band intensity for each condition is noted in red above each bar. Significance was calculated using 2-way ANOVA followed by Holm–Sidak’s multiple comparisons post hoc test. * indicates adjusted *P* < 0.05 with all *P* < 0.0001.

To quantify ORAI1 protein expression, 3 commercially available antibodies claimed to be specific for ORAI1 and a rabbit polyclonal antibody targeting the first 20 amino acids of the ORAI1 N-terminus were tested, but none resulted in a clear band for ORAI1 at the expected molecular weight using skeletal muscle lysates (Fig. EV4A). To overcome this challenge, we generated a knock-in mouse in which a V5-3xHA epitope was added to the extreme C-terminus of one ORAI1 allele, hereafter referred to as heterozygous *Orai1*^*V5HA/+*^ mice (or V5HA/+ mice). Compared to WT mice, V5HA/+ mice exhibited a similar physical appearance, body mass, and SOCE activity (Fig. 5C,D). Immunohistochemistry of V5HA/ + EDL muscle fibers using a HA antibody and an ORAI1 antibody that was previously shown to be specific for ORAI1 in immunocytochemical (but not western blot) studies (Boncompagni et al, 2018) revealed clear co-localization (Fig. 5E). Together, these results demonstrate that the V5-3xHA epitope on the ORAI1 C-terminus does not alter ORAI1 function or subcellular localization. Western blot analysis using the same rat-HA antibody showed two prominent ORAI1-V5-3xHA bands between 35 and 50 kDa in *tibialis anterio*r muscle lysates from V5HA/+ mice, but not in lysates from WT mice (Fig. 5F). Similar HA-reactive bands were also observed in other diaphragm and limb (including *tibialis anterior*, EDL, SOL, *quadricep*, *gastrocnemiu*s, and FDB) muscle lysates (Fig. EV4B), as well as multiple other tissues including brain, heart, spleen, lung, liver, kidney, pancreas, and submandibular gland (Fig. EV4C), thus validating the use of V5HA/+ mice for western blot quantification of endogenous ORAI1 isoforms in multiple tissues. Interestingly, co-IP studies using an N-terminal ORAI1 antibody revealed that the larger ORAI1 band in western blots of muscle spleen likely represents the full-length ORAI1 protein (Fig. EV4E), while the lower band likely represents a smaller isoform that was reported previously to result from an alternate translation initiation site (Desai et al, 2015; Fukushima et al, 2012). Both the short and long ORAI1 isoforms exhibit significant glycosylation (Fig. EV4D,E).

We used V5HA/+ mice to quantify WT ORAI1 protein expression in WT and GS mice by crossing these mice with GS mice to generate V5-3xHA-tagged WT (*Orai1*^*V5HA/+*^) and GS (*Orai1*^*V5HA/GS*^) mice. Assuming similar transcription/translation of *Orai1 * from WT and GS alleles, HA western blots of *tibialis anterior* muscle lysates from *Orai1*^*V5HA/+*^ (V5HA/+) and *Orai1*^*V5HA/GS*^ (V5HA/GS) mice were used to directly quantify WT ORAI1 protein level (actually, ORAI1-V5-3xHA), and by extrapolation, GS ORAI1 protein expression. Due to the combination of alternative ORAI1 translation and differential levels of glycosylation, ORAI1 HA blots included multiple bands ranging from 30 kDa to 50 kDa (see Fig. EV4B). We quantified both the major upper and lower bands, likely representing the full length and N-terminal truncated ORAI1 proteins (Desai et al, 2015; Fukushima et al, 2012), respectively. Total HA reactivity was also measured as the sum of the two bands (Fig. 5G, H, left). Compared to that of age-matched control V5HA/+ mice, all three measures (total, upper, and lower bands) were significantly reduced in skeletal muscle lysates from both 2 WK old (Fig. 5G, right) and 8 M old (Fig. 5H, right) V5HA/GS mice. In addition, PNGase treatment of *tibialis anterior* muscle lysates from 8 M old V5HA/+ and V5HA/GS mice revealed a similar fractional reduction (~60%) of both the long and short ORAI1 isoforms (Fig. EV4F).

To determine if the reduction in total ORAI1 protein expression observed in skeletal muscle of GS mice was due to a reduction in *Orai1* transcript level, we quantified *Orai1* mRNA in *tibialis anterior* muscle of WT and GS mice using RT-qPCR. These studies found no significant difference in *Orai1* transcript levels (normalized to either *Gapdh* or *Rpl7l1*) in skeletal muscle of either 2 WK or 8 M old WT and GS mice (Appendix Fig. S2A,B).

In addition to reduced ORAI1 expression, another possible reason for the observed reduction in SOCE activity is ORAI1 mis-localization (e.g. sequestration into TAs). Hence, ORAI1 subcellular distribution in relation to both STIM1 (located throughout the I band) and RYR1 (located at the T-tubule-SR junction) were measured. Immunocytochemical analyses of EDL muscle bundles from 8 M old V5HA/+ and V5HA/GS mice revealed that ORAI1 localization in myofibers from GS mice exhibits a normal distribution in double transverse rows (i.e. in transverse tubules) that nicely co-localizes with RYR1 at the T-tubule-SR junction and is flanked by STIM1 (Appendix Fig. S3). These studies indicate that ORAI1 localization is unaltered in muscle fibers of GS mice.

### Altered Ca^2+^ handling in GS mice

**Figure EV5:**
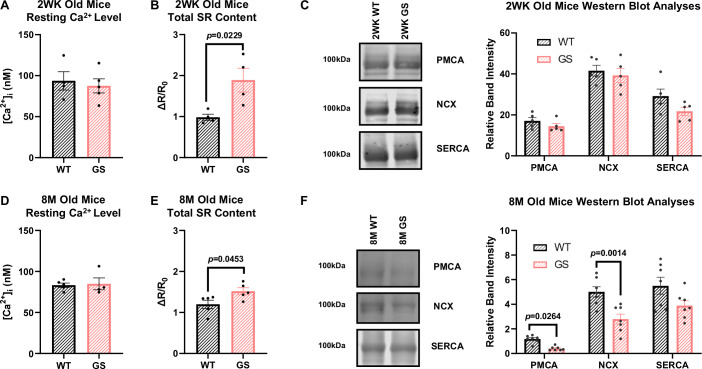
GS mice show altered Ca^2+^ handling ability at both 2 WK and 8 M of age. (**A**, **D**) No significant alterations in resting Ca^2+^ level were observed in FDB fibers from GS mice at both 2 WK and 8 M of age. For 2 WK old mice, *n* = 4 and 5 for WT and GS mice, respectively. For 8 M old mice, *n* = 5 and 4 for WT and GS mice, respectively. (**B**, **E**) SR Ca^2+^ storage was significantly increased in FDB fibers from GS mice at both 2 WK and 8 M of age. For 2 WK old mice, *n* = 4 WT mice and *n* = 4 GS mice. For 8 M old mice, *n* = 5 WT mice and *n* = 5 GS mice. Significance was calculated using Student’s *t* test. The exact *P* value is labeled in the figure when *P* < 0.05. (**C**) Expression of PMCA, NCX and SERCA were not significantly altered in *tibialis anterior* muscle lysates from 2 WK old GS mice. Protein expression levels were normalized to Ponceau S. *n* = 5 for all experiment groups. (**F**) Expression of PMCA and NCX, but not SERCA, were significantly decreased in *tibialis anterior* muscle lysates from 8 M old GS mice. Protein expression levels were normalized to Ponceau S. *n* = 5 for all experiment groups. Significance was calculated using 2-way ANOVA followed by Holm–Sidak’s multiple comparisons post hoc test. The exact *P* value is labeled in the figure when adjusted *P* < 0.05.

Despite the increase in constitutive Ca^2+^ entry observed in GS mice in early development (i.e. myotubes up to 5 weeks of age, Fig. 4D), the resting Ca^2+^ concentration in FDB fibers from 2 WK old GS mice was not significantly different from that of age-matched WT mice (Fig. EV5A). To explain this, we measured the expression of proteins involved in cytoplasmic Ca^2+^ efflux (PMCA, NCX and SERCA) and total releasable Ca^2+^ store content in muscle from 2 WK old WT and GS mice. While no change in expression of Ca^2+^ efflux proteins was observed, total Ca^2+^ store content was significantly increased (Fig. EV5B,C). Similarly, at 8 M of age, the resting Ca^2+^ concentration in FDB fibers from GS mice was unaltered (Fig. EV5D), while total Ca^2+^ store content was significantly elevated (Fig. EV5E). However, expression of proteins involved in Ca^2+^ efflux (i.e. PMCA and NCX expression) was significantly reduced (Fig. EV5F). Ca^2+^ sequestered in TAs of muscle fibers from 8 M old GS mice could contribute to the increase in total releasable Ca^2+^ store content observed at this age, consistent with that suggested previously (Bohm et al, 2017; Bohm et al, 2013; Endo et al, 2015; Garibaldi et al, 2017; Harris et al, 2017; Walter et al, 2015).

In addition to SR, mitochondria can act as a minor intracellular Ca^2+^ storage compartment. However, experiments measuring activity-dependent mitochondrial Ca^2+^ uptake and efflux revealed no significant difference in FDB fibers from 8 M old WT and GS mice (Appendix Fig. S4).

### Changes in the skeletal muscle proteome

**Figure 6:**
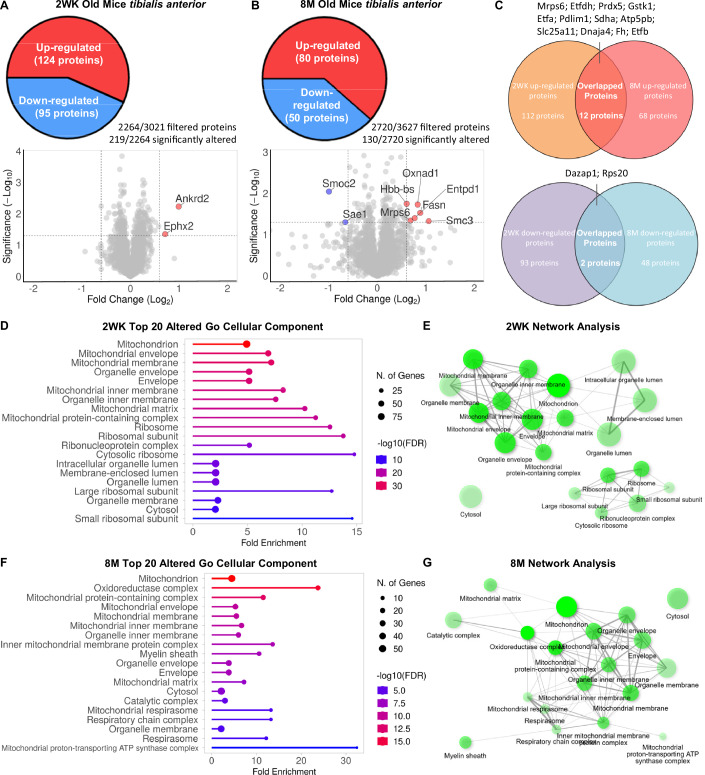
Proteome analyses of *tibialis anterior* muscle from 2 WK and 8 M old WT and GS mice. (**A**, **B**) Pie charts (upper) and volcano plots (lower) of normalized abundance data depicting the proportion of significantly up (red) and down (blue) regulated proteins in *tibialis anterio*r muscles of 2 WK (**A**) and 8 M (**B**) old WT and GS mice. Data were collected from 2 WT and 4 GS 2 WK old mice and 3 WT and 4 GS 8 M old mice. (**C**) Venn analysis of proteins that were significantly upregulated (upper panel) and downregulated (lower panel) in *tibialis anterior* muscles of 2 WK and 8 M old WT and GS mice. (**D**–**G**) Top 20 altered GO Cellular Component pathways (**D**, **F**) and associated network analyses (**E**, **G**) from proteomic analyses of *tibialis anterior* muscles from 2 WK and 8 M old WT and GS mice. Data were collected from 2 WT and 4 GS 2 WK old mice and 3 WT and 4 GS 8 M old mice.

We used quantitative mass spectrometry to compare the skeletal muscle proteome of *tibialis anterior* muscles from 2 WK and 8 M old WT and GS mice. From 3021 proteins identified in *tibialis anterior* muscles of 2 WK old mice, 219 proteins were significantly different in GS mice (124 upregulated and 95 downregulated proteins) (Fig. 6A, upper panel). Among these, only Ankrd2 and Ephx2, both upregulated, exhibited a log2 fold change >0.6 (Fig. 6A, lower panel). A similar analysis conducted of *tibialis anterior* muscles from 8 M WT and GS mice revealed a total of 130 significantly altered proteins (80 upregulated and 50 downregulated) (Fig. 6B, upper panel). Volcano plot analysis indicated that 2 out of the 50 downregulated proteins and 6 of the 80 upregulated proteins exhibited a log2 fold change greater >0.6 (Fig. 6B, lower panel). We also examined the degree of overlap between the significantly upregulated and downregulated proteins between the two ages (Fig. 6C). Interestingly, among the 12 common upregulated proteins, 8 were mitochondrial-related proteins (Slc25a11, Mrps6, Etfdh, Etfa, Etfb, Sdha, Atp5pd, Fh) involved in either mitochondrial respiratory chain function or mitochondrial lipid oxidation. Modifications in mitochondrial-related pathways in muscle of GS mice at both ages was further supported by Go Cellular Component and network analyses (Fig. 6D–G). In fact, ≥8 out of the top 10 altered Go Cellular Component terms were related to mitochondria at both 2 WK (Fig. 6D) and 8 M (Fig. 6F) of age. A unique proteome difference observed at these two ages was the specific involvement of ribosomal-related pathways (6 of the top 20 terms) at 2 WK of age but not at 8 M of age. Network analyses of the top 20 Go Cellular Component pathways revealed two largely separate network clusters at 2 WK (mitochondrial and ribosomal networks, Fig. 6E) with primarily a single major mitochondrial network observed at 8 M of age (Fig. 6G). For both ages, the respective network clusters were tightly interconnected and not connected to an alternate cluster. Taken together, these findings suggest a strong phenotype of altered mitochondrial pathways, suggesting potential alterations in mitochondrial structure and function in skeletal muscle of GS TAM mice.

### Altered mitochondrial morphology and function in muscle of GS mice

**Figure 7:**
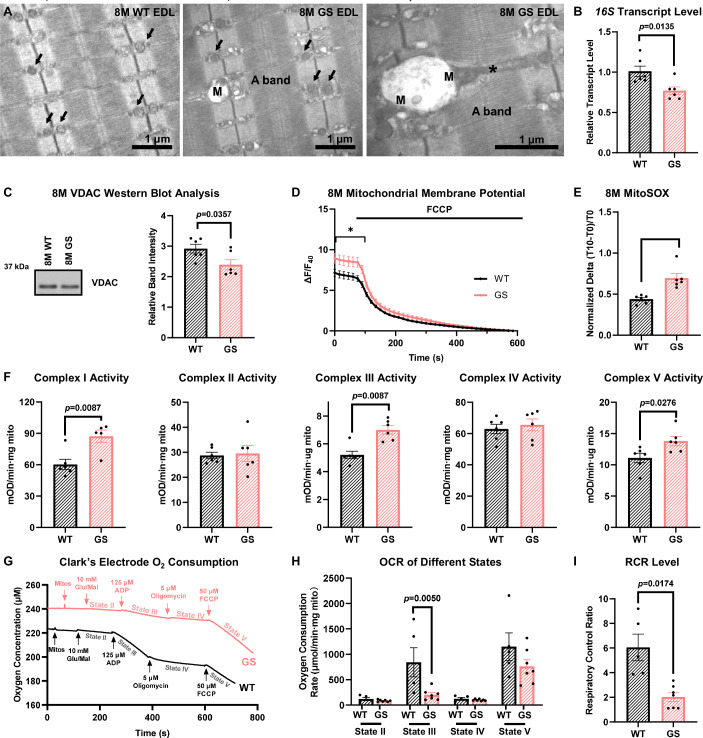
Morphological and functional assessment of mitochondrial damage in 8 M GS mice. (**A**) Representative EM images of longitudinal sections of EDL muscles from 8 M old WT and GS mice. Arrows: mitochondria at I band; M: severely altered mitochondria; *: mitochondrion at the A band. (**B**) Average ( ± SEM) *16S rRNA* transcript level in 8 M WT (*n* = 6) and GS (*n* = 6) *tibialis anterior* muscles using RT-qPCR. *16S* is a mitochondria specific ribosomal RNA. Significance was calculated using Student’s *t* test. The exact *P* value is labeled in the figure when *P* < 0.05. (**C**) Representative western blots (left) and average ( ± SEM) bar graphs (right) for VDAC expression in *tibialis anterior* muscles from 8 M old WT (*n* = 6) and GS (*n* = 6) mice. Protein expression levels were normalized to Ponceau S. Significance was calculated using Student’s *t* test. The exact *P* value is labeled in the figure when *P* < 0.05. (**D**) Normalized average ( ± SEM) TMRE fluorescence (normalized to the final 40th measurement, ΔF/F_40_) of single FDB fibers from 8 M old WT (*n* = 6) and GS (*n* = 6) mice before and during application of 1 µM FCCP (black bar). Significance was calculated using Student’s *t* test. * indicates adjusted *P* < 0.05 (adjusted *P* = 0.000035, 0.000035, 0.00004, 0.000034, 0.000028, 0.000003, 0.000018 in sequential order). (**E**) Normalized average ( ± SEM) delta MitoSOX fluorescence over 10 min using FDB fibers from 8 M WT and GS mice. MitoSOX is a mitochondrial specific superoxide indicator, used to indicate mitochondrial oxidative stress level. Significance was calculated using Mann–Whitney test. The exact *P* value is labeled in the figure when *P* < 0.05. (**F**) Average ( ± SEM) enzyme activity of proteins involved in electron transport chain (Complex I–V) using purified mitochondria from skeletal muscle of 8 M old WT and GS mice. For Complex I activity, *n* = 5 and 6 WT and GS mice, respectively. For complex II–V, *n* = 6 for both WT and GS mice. Significance was calculated using either Mann–Whitney test (Complex I and III activity) or Student’s *t* test (Complex V activity). The exact *P* value is labeled in the figure when *P* < 0.05. (**G**) Representative Clark’s electrode oxygen consumption traces using purified mitochondria from 8 M WT (black) and GS (pink) skeletal muscle. (**H**, **I**) Average ( ± SEM) oxygen consumption rate (OCR) at each state (**H**) and average ( ± SEM) respiratory control ratio (RCR – State III/State IV) **(I)** in purified mitochondria from 8 M WT (*n* = 5) and GS (*n* = 7) skeletal mice. Significance was calculated using either two-way ANOVA followed by Holm–Sidak’s multiple comparisons post hoc test (OCR measurements) or Welch’s *t* test (RCR level). The exact (adjusted) *P* value is labeled in the figure when *P* < 0.05. Source data: available online for (**A**–**I**).

To validate the proteomic findings, we quantified mitochondrial morphology in EDL muscles from 8 M old WT and GS mice using EM analyses (Fig. 7A). These analyses revealed in EDL muscles from GS mice: (i) the number of severely altered mitochondria/100 mm^2^ (defined as disrupted external membranes, vacuolation of internal membranes, and/or myelin figure formation) was significantly increased, (ii) even morphologically normal mitochondria were significantly larger in size, indicating mitochondrial swelling (Favaro et al, 2019), and (iii) the number of mitochondria at the A band was reduced (Table 1). Quantitative measurements of mitochondrial *16S rRNA* and VDAC expression revealed a modest, but significant, reduction in mitochondrial content in skeletal muscle of 8 M old GS mice (Fig. 7B,C).

**Table 1:** Quantitative EM analysis of mitochondria in EDL muscles from 8 M WT (*n* = 5) and GS (*n* = 7) mice.

	No. of mito/100 μm^2^	No. of mito at A band/100 μm^2^ (%)	No. of severely altered mito/100 μm^2^ (%)	Average size of mito (× 10^−3^ nm^2^)
8 M WT	51.0 ± 1.9	1.23 ± 0.22 (2.4)	1.00 ± 0.14 (1.9)	32.7 ± 0.7
8 M GS	53.0 ± 2.2	0.70 ± 0.15 (1.3)*	4.92 ± 0.44 (9.2)*	46.2 ± 1.1*

^*^P < 0.05.

The mitochondrial membrane potential (ΔΨ_M_) is an important indicator of mitochondrial health. Using TMRE, a fluorescent indicator of ΔΨ_M_, we found FDB fibers from GS mice exhibited a significant increase in basal TMRE fluorescence and addition of 1 μM FCCP resulted in a time-dependent and complete dissipation of TMRE fluorescence (Fig. 7D). As a result, both the basal and FCCP-sensitive TMRE responses were significantly increased in FDB fibers from GS mice, consistent with a more negative ΔΨ_M_. Since the rate of mitochondrial reactive oxygen species (ROS) production is strongly dependent on ΔΨ_M_ (Starkov and Fiskum, 2003), we used a mitochondrial ROS sensor, MitoSOX Red, to directly assess the rate of mitochondrial ROS production in acutely dissociated FDB fibers from 8 M old WT and GS mice. These studies found that the rate of increase in MitoSOX fluorescence was significantly increased in FDB fibers from 8 M GS mice (Fig. 7E), thus confirming increased mitochondrial oxidative stress.

There are several possible explanations for the observed increase in ΔΨ_M_ and oxidative stress, including altered function of proteins involved in the mitochondrial electron transport chain and disrupted oxidative phosphorylation. To investigate the first possibility, we measured the activity of complexes I-V in the mitochondrial electron transport chain using mitochondria purified from hindlimb skeletal muscle. Compared to age-matched WT mice, the activity of Complex I, III and V were significantly increased in mitochondria purified from 8 M GS mice (Fig. 7F). Next, a Clark-type electrode was used to quantify mitochondrial oxygen consumption rate (OCR) in mitochondria purified from hindlimb skeletal muscle of 8 M old WT and GS mice (Fig. 7G). Maximal ADP-stimulated OCR (State III) was significantly reduced in mitochondria isolated from GS mice, while OCRs of all other respiratory states (States II, IV, and V) were not significantly different (Fig. 7H). As a result, the respiratory control ratio (RCR), calculated as the ratio of State III OCR/State IV OCR (similar results were also obtained for State III/State II), was significantly reduced in mitochondria purified from skeletal muscle of GS mice (Fig. 7I).

## Discussion

### Background

TAM results from GoF mutations in ORAI1 and STIM1 leading to over activation of SOCE, a fundamental mechanism refilling ER/SR Ca^2+^ stores (Lacruz and Feske, 2015; Morin et al, 2020). Four different preclinical animal models of TAM due to GoF mutation in STIM1 have been developed, involving mutations in the STIM1 coiled-coil region (*Stim1*^*R304W/+*^ mice) (Gamage et al, 2018; Silva-Rojas et al, 2019) and STIM1 EF hand domains (*Stim1*^*I115F/+*^ and *Stim1*^*D84G/+*^ mice) (Cordero-Sanchez et al, 2020; Grosse et al, 2007). Although these STIM1 mouse models exhibit several clinical hallmarks for TAM, including abnormal bleeding phenotypes and muscle weakness, none exhibit TAs, a key histological manifestation on TAM in humans. On the other hand, while six different GoF mutations in ORAI1 are known to result in TAM (Bohm et al, 2017; Endo et al, 2015; Garibaldi et al, 2017; Nesin et al, 2014), no TAM models based on ORAI1 GoF TAM mutations have been reported. Thus, we generated a mouse model of TAM based on a glycine-to-serine point mutation in the ORAI1 pore (G98S in human or G100S in mouse), which was identified in at least three different families with an early-onset form of TAM (Bohm et al, 2017; Endo et al, 2015). Individuals with the G98S mutation present with childhood disease onset, muscle weakness, cramps, stiffness, hypocalcemia, and muscle histological signatures including TAs, fiber size variability, type I fiber predominance, internal nuclei, and fibrosis (Bohm and Laporte, 2018; Michelucci et al, 2018; Morin et al, 2020). G98 forms a gating hinge at a rigid section of the channel pore that permits channel opening upon activation (Zhang et al, 2011). The G98S mutation causes this gate to be constitutively open independent of STIM1, thus resulting in a GoF impact on SOCE (Bohm et al, 2017; Garibaldi et al, 2017).

### Breeding of GS mice

Though GS mice are fertile and born at a normal Mendelian ratio, homozygous inheritance of the mutation was found to be embryonic lethal. Among the 5 inbreeds of heterozygous mice, no *Orai1*^*G100S/G100S*^ embryos were obtained. In fact, even when sacrificing pregnant females at 14.5–16.5 days post-conception, no evidence of resorption was observed. This observation indicates a critical role of ORAI1 in early pregnancy that was not previously appreciated. Reasons for the embryonic lethality could be inefficient fertilization, impaired cell polarization, defects in blastocyst development or inadequate egg implantation. However, since the average number of embryos when interbreeding heterozygous animals was 7 embryos/litter, which is even greater than that when breeding heterozygous with WT mice, it is unlikely that the observed embryonic lethality occurs after fertilization as a reduction in litter size was not observed.

### Phenotype of GS mice

GS mice phenocopy multiple key aspects observed in TAM patients due to the G98S mutation in ORAI1. Firstly, significant muscle weakness and exercise intolerance are observed in GS mice based on both behavioral tests (grip strength, rotarod, treadmill) and ex vivo muscle contraction measurements. These results are consistent with impaired endurance, running, and sprinting deficits reported in humans with TAM (Bohm et al, 2017; Bohm and Laporte, 2018; Endo et al, 2015; Morin et al, 2020). In addition, similar to that reported for TAM patients with this mutation, GS mice also exhibit a significant hypocalcemia and increase in serum CK levels. However, skeletal muscle from GS mice showed no evidence of major muscle damage or necrosis since significant changes in fiber size/type, central nucleation and/or fibrosis were not detected. Thus, GS mice likely exhibit only a relatively mild degree of muscle damage that is manifested in terms of a relatively moderate increase in serum CK level.

The extent of hematological dysfunction in mouse models of TAM varies depending on the underlying mutation. In this regard, bleeding disorders and/or anemia observed clinically are recapitulated in multiple STIM1 mouse models of TAM (Cordero-Sanchez et al, 2020; Grosse et al, 2007; Silva-Rojas et al, 2019). On the other hand, anemia was only reported in 1 out of 6 TAM individuals resulting from the ORAI1 G98S mutation, while thrombocytopenia has not been reported in any *Orai1*^*G98S/+*^ patients (Bohm et al, 2017; Endo et al, 2015). Consistent with this, we found no evidence of changes in platelets, red blood cells, or bleeding/clotting function in GS mice.

Most importantly, we found that multiple fast-twitch muscles from 8 M GS mice (FDB, EDL and *tibialis anterior* muscles) exhibit a robust presence of honeycomb-like structures of TAs that stain pink in Gomori trichrome. The age-dependent formation of TAs in skeletal muscle of GS mice make this an ideal model to study the mechanisms that underlie TA formation/stability, protein present in TAs, and the role of these structures in the disease. Coincidentally, beginning at 8 M of age, the same time when TAs are readily observed, GS mice begin to become smaller than age-matched WT mice.

However, it is important to also note that some aspects of TAM in patients with the G98S ORAI1 mutation were not observed in 8 M old GS mice. Specifically, individuals with TAM due to the G98S ORAI1 mutation exhibit significant fiber size variability, type I fiber predominance, internal nuclei and fibrosis on muscle biopsies, none of which were routinely observed in muscle of 8 M old GS mice. The reasons for these differences are unclear but could involve differences between disease presentation in mice versus humans and/or some of these changes may occur in GS mice at more advanced ages. Future studies designed to identify later-onset phenotypes as GS mice age (e.g. from 12 to 24 months) are needed to address this issue.

### Presence of TAs and muscle disease

The relative importance of TAs in the clinical myopathy experienced by TAM patients is largely unknown. In fact, TAs are not only observed in TAM, but also occur in a variety of other inherited diseases such as phosphoglycerate mutase deficiency (Oh et al, 2006), congenital myasthenic syndromes (Belaya et al, 2012; Cossins et al, 2013; Guergueltcheva et al, 2012) and periodic paralysis (Sternberg et al, 2001). One common feature often associated with TAs is myopathy, in which patients exhibit myalgia, muscle cramps and stiffness. Besides being found in disease models, TAs are also observed during aging in inbred male mice where they also preferentially assemble in fast-twitch fibers (Boncompagni et al, 2012; Chevessier et al, 2005; Leoty, 1984). However, although TAs are associated with several myopathies, their relative contribution to disease progression and muscle weakness, even in TAM, remains unclear.

### G98S mutation and channel function

Structural studies carried out by *Zhang* et al demonstrate that glycine 98 in ORAI1 (corresponding to glycine 100 in mice) is located at the pore forming region of ORAI1 and once mutated, leads to constitutive, STIM1-independent activation (Zhang et al, 2011). This finding is consistent with prior in vitro studies where ORAI1-G98S was transiently expressed in HEK293 cells and in myotubes derived from patient muscle biopsies (Bohm et al, 2017; Endo et al, 2015). Similarly, we found constitutive Ca^2+^ entry in HEK293 cells transfected with mORAI1-G100S and myotubes derived from GS mice. Together, these results confirm the GoF effect of the G98S (and G100S) mutation in the ORAI1 pore region. Interestingly, we also found that in HEK293 cells, co-expression of WT mORAI1 together with G100S mORAI1 markedly reduced (~80%), but did not eliminate, constitutive Ca^2+^ entry. This indicates that incorporation of WT ORAI1 subunits markedly limits the degree of constitutive Ca^2+^ entry resulting from the G100S mutation, which likely explains the embryonic lethal phenotype of homozygous *Orai1*^*G100S/G100S*^ mice and survival with a slowly progressive myopathy of GS mice.

### Compensatory response of muscle fibers from GS mice to reduce Orai1 expression

We found that constitutive Ca^2+^ entry in muscle fibers from GS mice was unexpectedly abolished after 5 WK of age (Fig. 4D). While increased basal Ca^2+^ entry is observed in myotubes derived from both GS mice and human TAM individuals caused by the ORAI1 G100S/G98S mutation, as well as immune cells from G98S patients and following expression in HEK293 cells (Bohm et al, 2017; Endo et al, 2015; Lian et al, 2018) (see also Fig. 4A), constitutive and store-operated Ca^2+^ entry have not previously been measured in adult muscle fibers from TAM patients or mouse models. Given the damaging effects that sustained Ca^2+^ entry would have on skeletal muscle (e.g. protease activation, necrosis, myofiber damage, regeneration) and the absence of a dystrophic phenotype in skeletal muscle in both TAM patients and GS mice (Fig. 1F), it seems likely the observed postnatal ablation of constitutive Ca^2+^ entry reflects an important protective adaptation of skeletal muscle. Indeed, inhibition of constitutive Ca^2+^ entry in adult skeletal muscle would limit activation of proteases such as calpains and matrix metalloproteinases (MMPs) that lead to degradations of cellular components (Murphy et al, 2006; Ren et al, 2019).

A remaining question involves the mechanism responsible for the observed postnatal reduction and elimination of constitutive Ca^2+^ entry. Our studies suggest that this is at least in part due to a reduction in overall ORAI1 protein expression (Figs. 5H and EV4F). Interestingly, a similar protective reduction of STIM1 protein expression was reported in skeletal muscle of homozygous *Stim1*^*R304W/R304W*^ mice (Gamage et al, 2018). Since *Orai1* transcript levels were unaltered in skeletal muscle of GS mice, the reduction in ORAI1 protein in GS mice likely involves an increase in the rate of ORAI1 protein degradation and/or altered ORAI1 post-translational modification. For example, if GS subunits exhibit misfolding and an increased rate of degradation, fully mature hexameric ORAI1 channels may exhibit a greater proportion of WT subunits, and thus, both reduced overall ORAI1 expression (and thus reduced SOCE) and constitutive Ca^2+^ entry. As WT ORAI1 channels suppress constitutive Ca^2+^ resulting from incorporation of GS subunits, a higher WT:GS subunit ratio would be expected to further limit constitutive Ca^2+^ entry. Future studies that compare WT and GS subunit degradation rate and WT:GS subunit ratio in skeletal muscle of GS mice (and muscle biopsies from G100S TAM patients) are needed to assess this explanation for the observed reduction in constitutive Ca^2+^ entry more rigorously. Additional potential mechanisms for the observed postnatal elimination of constitutive Ca^2+^ entry in muscle fibers from GS mice include a inhibitory post-translational modification (Johnson et al, 2022) or recruitment of an inhibitory protein to the ORAI1 complex.

### Ca^2+^ store content

In addition to age-dependent changes in constitutive and store-operated Ca^2+^ entry, total releasable Ca^2+^ store content was significantly increased in FDB fibers at both 2 WK and 8 M of age (Appendix Fig. S3A,D), though the reasons for these changes in Ca^2+^ store content are likely different. At 2 WK of age, the increase in Ca^2+^ store content likely reflects a steady-state effect of increased sequestration of Ca^2+^ into the SR due to the constitutive Ca^2+^ entry observed at this age. On the other hand, at 8 M of age when constitutive Ca^2+^ entry is absent, the increase in Ca^2+^ store content could result from either a reduction in net Ca^2+^ efflux due to decreased PMCA and NCX expression (Appendix Fig. S3F) and/or sequestration of Ca^2+^ in TAs of SR origin, which are present at this age (Bohm et al, 2017; Bohm et al, 2013; Endo et al, 2015; Garibaldi et al, 2017; Harris et al, 2017).

### Impact of SOCE dysfunction on mitochondria

There are two major populations of mitochondria in adult fast-twitch skeletal muscle: (1) a population located within the subsarcolemmal space and (2) a larger population found within the intermyofibrillar region, almost exclusively present within the I band between triads and Z-lines (Bleck et al, 2018; Glancy et al, 2015; Hoppeler et al, 1973). Given that STIM1-ORAI1 coupling and SOCE is similarly located within the triad and I band region of the sarcomere, alterations in constitutive and store-operated Ca^2+^ entry could impact mitochondrial structure and function (Boncompagni et al, 2018). Consistent with this, prior studies using both patient samples and mouse models have demonstrated the importance of SOCE activity in regulating mitochondrial function in skeletal muscle. For example, myotubes derived from mice with STIM1 loss-of-function mutation (R429C) exhibit ultra-structural defects in mitochondria, including “onion-shaped” cristae, and a significant decrease in ATP levels (Choi et al, 2019). Moreover, mitochondria are swollen in skeletal muscle-specific STIM1 knockout mice (Li et al, 2012; Stiber et al, 2008). Muscle cells derived from a TAM patient resulting from a L96V STIM1 variant exhibit altered morphology and geometry of the mitochondrial network (Conte et al, 2021). Together, these findings indicate a crucial role for SOCE activity in regulating mitochondrial structure and function. In turn, mitochondrial activity can also regulate SOCE function (e.g., the mitochondrial permeability transition pore negatively regulates SOCE activity) (Ben-Kasus Nissim et al, 2017; Haworth and Hunter, 1979). SOCE activity can then impact multiple downstream metabolic pathways including glycolysis, ATP production and mitochondrial oxidation (Henke et al, 2012; Nan et al, 2021; Rossi et al, 2019; Vaeth et al, 2017; Wilson et al, 2022).

Although we found that activity-dependent mitochondrial Ca^2+^ uptake and efflux were unaltered in muscle fibers from 8 M old GS mice (Appendix Fig. S3), substantial alterations in multiple mitochondrial-related pathways were identified in proteomic studies of *tibialis anterior* muscle from both 2 WK old and 8 M old GS mice (Fig. 6), consistent with an intimate interplay between SOCE activity and mitochondrial function. The fact that mitochondrial pathways were altered at both ages is interesting since an increase in constitutive Ca^2+^ entry was only observed at the younger age, while SOCE was abolished only at older ages (Fig. 4).

### Mitochondrial damage and dysfunction

Morphological and functional alterations of mitochondria were reported previously in multiple mouse models with a broad range of skeletal muscle disorders, including Duchenne muscular dystrophy and cachexia (Carson et al, 2016; Padrao et al, 2013; Pant et al, 2015). Our results extend these observations to a mouse model of TAM due to a GoF G100S mutation in ORAI1. Specifically, EM analyses of EDL muscle from 8 M old GS mice revealed a significant increase in mitochondrial size, increase in the number of severely damaged mitochondria/μm^2^, and decrease in the number of mitochondria at the A band. Interestingly, while EM analyses found no change in the number of mitochondria/100 μm^2^ in EDL muscle, a modest reduction in both mitochondrial transcript and VDAC protein was observed in *tibialis anterior* muscle. This apparent discrepancy could be due to several factors including differences in the muscles and approaches used, as well as the possibility that a selective modest reduction in subsarcolemmal mitochondria would not be represented in our EM analyses that only evaluated internal sarcomeric regions of the myofiber (i.e., only intermyofibrillar mitochondria were analyzed). Alternatively, the damaged mitochondria identified by EM might be under-represented in the RT-qPCR and VDAC measurements.

In line with the mitochondrial damage and structural alterations, mitochondrial respiratory function as assessed from Clark’s electrode measurements of oxygen consumption revealed a > threefold reduction in RCR from purified mitochondria isolated from skeletal muscle of 8 M old GS mice. OCR measurements at different states revealed that this reduction was due exclusively to a marked reduction in State III (ADP-stimulated) respiration. Additionally, mitochondria isolated from GS skeletal muscle exhibited significantly increased ΔΨ_m_ and mitochondrial ROS production, consistent with a potential role for increased ROS and oxidative stress damage in muscle of GS mice. The observed increase in complex I and III activities (primary sites of mitochondrial ROS production) in mitochondria isolated from GS (potentially a compensation for reduced RCR activity) would be expected to increase mitochondrial ROS production. Importantly, since ROS generation from complex I is strongly (non-linearly) dependent on ΔΨ_M_ (Starkov and Fiskum, 2003), these results are consistent with the observed increase in mitochondrial ROS generation originating largely from complex I in skeletal muscle of GS mice.

### Closing remarks

Results from this study demonstrate that GS mice phenocopy multiple key aspects of TAM in humans including exhibiting an age-dependent myopathy characterized by muscle weakness, exercise intolerance, hypocalcemia, and elevated CK levels. Unlike prior STIM1-based mouse models of TAM, GS mice also exhibit a robust presence of TAs in multiple fast-twitch muscles and lack a measurable bleeding phenotype, characteristics also observed in TAM patients due to the analogous ORAI1 mutation (G98S). Unexpectedly, we also found that muscle fibers from GS mice exhibit a marked loss-of-function in SOCE activity across all ages, due at least in part to a reduction in ORAI1 protein levels. Moreover, constitutive Ca^2+^ entry due to the G100S pore GoF mutation observed at early developmental stages was abolished during postnatal development, likely reflecting a skeletal muscle adaptation designed to limit Ca^2+^ induced muscle damage and necrosis. Additionally, we demonstrated significant mitochondrial damage and reduced respiratory function in skeletal muscle from GS mice. This not only suggests an important role of mitochondria in TAM, but also indicates an intimate interaction between SOCE activity and mitochondrial function in skeletal muscle.

Several intriguing questions remain unanswered. For example, while a reduction in both maximal muscle-specific force production and rate of relaxation were observed in both fast-twitch (EDL) and slow twitch (SOL) muscles, TAs were only found in fast-twitch muscles of GS mice. Thus, TAs are apparently not required for the functional deficits observed in skeletal muscle of GS mice, as was concluded previously for *Stim*^*R304W/+*^ mice (Silva-Rojas et al, 2019). In addition, while this study focused primarily on the muscle phenotype of GS mice, ORAI1 is expressed and plays important roles in many other cell types (e.g. immune cells, platelets, neurons, and cancer cells) (Prakriya and Lewis, 2015). Thus, it will be important for future studies to use GS mice to assess the impact of the G100S TAM mutation on SOCE activity and the physiological function of these non-muscle systems.

## Methods

**Table:** Reagents and tools table

Reagent/resource	Reference or source	Identifier or catalog number
**Experimental models**		
HEK-293 *(H. sapiens)*	ATCC	CRL-1573
Myoblast primary cell (*M. musculus)*	This study, isolated from newborn pups or embryos at embryonic day 14	
C57BL6/J *(M. musculus)*	The Jackson Laboratory	000664
*Orai1* ^*G100S/+*^ *(M. musculus)*	This study	N/A
*Orai1* ^*V5HA/+*^ *(M. musculus)*	This study	N/A
*Orai1* ^*V5HA/GS*^ *(M. musculus)*	This study	N/A
**Recombinant DNA**		
pYC3.60-C1	Addgene	# 67899
mt-YC3.6	This study	N/A
**Antibodies**		
Rabbit anti-laminin	Sigma-Aldrich	L9393
Mouse anti-MHC I	DSHB	BA-D5
Mouse anti-MHC IIa	DSHB	SC-71
Mouse anti-MHC IIb	DSHB	BF-F3
Mouse anti-ORAI1	Santa Cruz Biotechnology	sc-377281
Mouse anti-ORAI1	Santa Cruz Biotechnology	sc-377281
Rabbit anti-ORAI1	AbClonal	A7412
Rabbit anti-ORAI1	ThermoFisher	28411-1-AP
Rabbit anti-ORAI1 (N-terminus)	Pocono Rabbit Farm Lab, purified with sequence ‘CHPEPAPPPSHSNPELPVSGG’	
Rat anti-HA	Sigma-Aldrich	11867423001
Rabbit anti-HA	Cell Signaling	#3724
Rabbit anti-STIM1	Sigma-Aldrich	S6197
Mouse anti-CASQ	ThermoFisher	MA3-913
Mouse anti-SERCA1/2/3	Santa Cruz Biotechnology	sc-271669
Mouse anti-PMCA	ThermoFisher	MA3-914
Mouse anti-NCX	Swant	R3F1
Rabbit anti-VDAC	Sigma-Aldrich	SAB5701340
Goat anti-mouse IgG1, Alexa Fluor™ 555	ThermoFisher	A-21127
Goat anti-mouse IgG2b, Alexa Fluor™ 350	ThermoFisher	A-21140
Goat anti-mouse IgM, Alexa Fluor™ 488	ThermoFisher	A-21042
Goat anti-rabbit IgG, Alexa Fluor™ 647	ThermoFisher	A-21244
Goat anti-rabbit IgG, Alexa Flour™ 488	ThermoFisher	A-11008
Goat anti-mouse IgG, Alexa Flour™ 594	ThermoFisher	A-11032
Anti-mouse IgG-HRP	Kindle Bioscience	R1005
Anti-rabbit IgG-HRP	Kindle Bioscience	R1006
Anti-rat IgG-HRP	Abcam	ab97057
Goat anti-mouse IgG DyLight 800	ThermoFisher	SA5-10176
Goat anti-rabbit IgG DyLight 800	ThermoFisher	SA5-10036
**Oligonucleotides and other sequence-based reagents**		
PCR primers	This study	Table 3
**Chemicals, enzymes and other reagents**		
Dulbecco’s Modified Eagle Medium	ThermoFisher	11995065
Fetal Bovine Serum	ThermoFisher	A5670701
Lipofectamine-2000	ThermoFisher	11668019
Triton X-100 Detergent	Bio-Rad Laboratories	1610407
Trizol	ThermoFisher	15596026
PNGase F	New England Biolab	P0704
Ponceau S	Sigma-Aldrich	P7170
ECL Substrate	Kindle Bioscience	R1002
Pierce™ Protein A/G Magnetic Beads	ThermoFisher	88802
RNeasy Mini Kits	Qiagen	74104
DNase I	ThermoFisher	EN0521
dNTPs	ThermoFisher	Y02256
oligo(dT)	ThermoFisher	18418020
SYBR Green FastMix	Quantabio	95072
SuperScript™ III Reverse Transcriptase	ThermoFisher	18080093
Fura-2, AM	ThermoFisher	2549271
Fura-FF, AM	AAT Bioquest	21027
MitoSOX™ Mitochondrial Superoxide Indicators	ThermoFisher	M36008
Tissue-Tek™ O.C.T. Compound	Sakura Finetek USA	4583
Fluoromount-G™ Mounting Medium	Southern Biotech	0100-20
Superfrost™ Plus Microscope Slides	ThermoFisher	12-550-15
i-STAT CG8+ cartridge	Abbot Point of Care Inc.	N/A
BD Microtainer^®^ Blood Collection Tubes (Lithium Heparin)	BD Bioscience	365985
BD Microtainer^®^ Blood Collection Tubes (EDTA)	BD Bioscience	365974
Micro Blood Collecting Tubes	ThermoFisher	02-668-25
Nitrocellulose Membrane	Bio-Rad Laboratories	1620115
S-Trap^™^ Micro Columns	Protifi	N/A
RC DC™ Protein Assay Kit	Bio-Rad Laboratories	5000122
Pierce™ BCA Protein Assay Kits	ThermoFisher	23225
Creatine Kinase Activity Assay Kit	Sigma-Aldrich	MAK116
Complex I Activity Microplate Assay Kit	Abcam	ab109721
Complex II Activity Microplate Assay Kit	Abcam	ab109908
Complex III Activity Microplate Assay Kit	Abcam	ab287844
Complex IV Activity Microplate Assay Kit	Abcam	ab109911
MitoCheck® Complex V Activity Assay Kit	Cayman Chemical	701000
**Software**		
GraphPad	GraphPad Software Inc.	N/A
DMC/DMA-HT	Aurora Scientific	N/A
Image Studio	LI-COR Biosciences	N/A
Clampfit	Molecular Devices	N/A
SightX-viewer	Jeol Ltd	N/A
ShinyGO 0.80	South Dakota State University	N/A
Oxytrace+	Hansatech	N/A
SMASH	10.1186/2044-5040-4-21	
ImageJ	https://imagej.net/	
UniProt	UP000000589_10090	
DIA-NN (version 1.8.1)	https://github.com/vdemichev/DIA-NN	
VolcaNoseR	https://huygens.science.uva.nl/VolcaNoseR/	
**Other**		
i-STAT	Abbot Point of Care Inc.	N/A
HemaTrue Veterinary Hematology Analyzer	Heska	N/A
Exer 3/6 Treadmill	Columbus Instruments	N/A
Rotamex-5	Columbus Instruments	N/A
ASI Muscle Contraction System	Aurora Scientific	N/A
StepOne Plus Real-Time PCR machine	Applied Biosystems	N/A
Kwik Quant Digital Imager	Kindle Bioscience	N/A
LI-COR System	LI-COR Biosciences	N/A
Oxytherm+ Clark-type oxygen electrode	Hansatech	N/A
DiATOME® Diamond Knives	Diatome	N/A
Leica Ultracut R	Leica Microsystems	N/A
Leica CM1800 Cryostat	Leica Microsystems	N/A
Speed Vac	Labconco	N/A
EASY-nLC™ 1200 System	ThermoFisher	N/A
Fusion Lumos Tribrid Mass Spectrometer	ThermoFisher	N/A
Megaview III Digital Camera	Olympus Soft Imaging Solutions	N/A
Keyence BZ-X800 Epifluorescence Microscope	Keyence	N/A
Epifluorescence Microscope with Photomultiplier Detection System	Chroma Technologies	N/A
D-Eclipse C1 Plus Confocal Microscope	Nikon	N/A
FP 505 Morgagni Series 268D Electron Microscope	FEI Company	N/A
JEM-1400 Flash Transmission Electron Microscope	Jeol Ltd	N/A
DeltaRam Illumination System	Photon Technology International	N/A

### Methods and protocols

#### Generation of *Orai1*^*G100S/+*^ and *Orai1*^*V5HA/+*^ knock-in mice

All animal procedures were conducted in accordance with institutional guidelines approved by the University Committee on Animal Recourses, University of Rochester Medical Center. Mice were housed under standard conditions with ad libitum access to water and proper chow with regulated ambient temperature and a standard 12:12 light/dark cycle.

Mice with knock-in of a glycine to serine mutation in ORAI1 at amino acid 100 (*Orai1*^*G100S/+*^ or GS mice) were generated by the Mouse Genome Editing Resource Facility at the University of Rochester Medical Center using CRISPR/Cas9 gene editing. Founder mice were generated on a congenic C57Bl/6 J background with genomic editing targeting exon 1 of the *Orai1* gene. To generate the mutant allele, a single-stranded DNA (ssDNA) donor template with sequence of 5’-GCTCTGCTCAGCTCCTTCGCGATGGTGAGCTCGGGCCTCCCTGCCCTCCTC-3’ was used to introduce the desired p.G100S mutation and silent changes at the protospacer adjacent motif (PAM) sequence in order to prevent re-cutting of the repaired allele. A specific guide RNA (sgRNA) with spacer sequence 5’-CGAGCTCACCATGGCGAAGCCGG-3’ was used for successful CRISPR/Cas9 insertions (Fig. EV1A). Cas9 mRNA, ssDNA, and sgRNA were microinjected into mouse embryos. Successful introduction of the G100S mutation was verified using Sanger sequencing (Fig. EV1B). Germline transmission was established from multiple founders, followed by subsequent outcrossing of the resulting F1 mice with congenic wild-type (WT) C57Bl/6 J mice to correct for potential off-target editing (Fig. EV1C). All experiments were performed on mice from the F7 generation onwards.

*Orai1*^*V5HA/+*^ mice were generated using a similar CRISPR/Cas9 approach with sgRNA targeting the end of *Orai1* exon 2 and a dsDNA template encoding the addition of a V5-3xHA epitope added to the extreme C-terminus of the ORAI1 protein (Table 2). Successful insertion of the V5-3xHA tag was verified using Sanger sequencing. Founder mice were outbred with congenic wild-type (WT) C57Bl/6 J mice to correct for potential off-target editing. All experiments were performed on mice from the F7 generation onwards. *Orai1*^*V5HA/+*^ mice were breaded with GS mice to produce V5-3xHA-tagged WT (*Orai1*^*V5HA/+*^) and GS (*Orai1*^*V5HA/GS*^) littermates.

**Table 2:** Generation of *Orai1*^*V5HA/+*^ mice using CRISPR/Cas9 technique.

sgRNA	5’-GGTGAGGACTTAGGCATAGTGGG-3’
dsDNA template	gctagcCGAGTTTGCCCGCTTGCAGGACCAGCTGGACCACAGAGGGGACCATTCTCTAACACCGGGtACtCACTAcGCtACGCGTGGAAAACCAATACCAAACCCACTACTAGGACTAGACAGCACATCGCGTTACCCATACGATGTTCCTGACTATGCGGGCTATCCCTATGACGTCCCGGACTATGCAGGATCCTATCCATATGACGTTCCAGATTACGCTTAGTAAggccctttgaggccttggccttatgcccttctccatgaccttgtcctggcatgtatgtgcgcacgctagc

#### Whole blood analysis

Mice were anesthetized by intraperitoneal injection of 100 mg/kg ketamine, 10 mg/kg xylazine and 3 mg/kg acepromazine. Blood samples were collected by cardiac puncture and put in a lithium-heparin collection tube (BD Bioscience) to prevent clotting. Whole blood measurements of ionized calcium, Na^+^, K^+^, glucose, and pH were performed using an i-STAT portable blood analyzer (Abbot Point of Care Inc.) where roughly 95 μL blood samples were added to each i-STAT CG8+ cartridge (Abbot Point of Care Inc.).

#### Serum CK level

After being anesthetized as described above, roughly 150 µL of blood was collected from the submandibular vein and put in a EDTA coated collection tube (BD Bioscience) to prevent clotting. Serum CK levels were assessed using a commercially available kit (Sigma-Aldrich) according to the manufacturer’s instruction. Mice were then either allowed to recover under technician supervision (when later time points were to be studied) or euthanized by anesthesia overdose and cervical dislocation (when terminal experiments were planned).

#### Tail bleeding test

After being anaesthetized as described above, the distal ~8 mm segment of their tail was amputated with a scalpel. The tail was then immediately immersed in a 50 mL Falcon tube containing pre-warmed isotonic saline in a water bath of 37 °C. The time needed for the tail to stop bleeding was recorded for 20 min. If bleeding continued beyond 20 min, then the experiment was terminated, and bleeding was stopped using Kwik-Stop Styptic Powder.

#### Complete blood count blood test

For complete blood count (CBC) values, ~50 μL of blood was obtained retro-orbitally from anesthetized mice using a heparinized 147 mm glass blood collection tube (Thermofisher) and analyzed using a HemaTrue Veterinary Hematology Analyzer (Heska).

#### Electron microscopy

EDL, FDB, and *tibialis anterior* muscles were processed as previously described (Michelucci et al, 2022). In brief, intact muscles were fixed at room temperature with 3.5% glutaraldehyde in 0.1 M sodium cacodylate buffer (NaCaCO) (pH 7.2) and stored at 4 °C until use. Fixed muscles were then post-fixed in 2% OsO_4_ for 1–2 h, rinsed with 0.1 M NaCaCO buffer, en-block stained with saturated uranyl acetate replacement, and embedded for electron microscopy in epoxy resin (Epon 812). Ultrathin sections were cut using a Leica Ultracut R microtome (Leica Microsystems) with a Diatome diamond knife (Diatome) and double-stained with uranyl acetate replacement and lead citrate. Sections were viewed on a FP 505 Morgagni Series 268D electron microscope (FEI Company), equipped with a Megaview III digital camera (Olympus Soft Imaging Solutions) and Soft Imaging System at 60 kV or with a 120 kV JEM-1400 Flash Transmission Electron Microscope (Jeol Ltd) equipped with CMOS camera Matataki and SightX-viewer software (Jeol Ltd). A computer‐assisted morphometric analysis of EM images taken from random, but not overlapping, fields were used to identify in transverse sections: (1) the number of fibers with TAs; (2) the cross‐sectional area (CSA) of each TA; and (3) the number of TAs in each fiber. Moreover, internal myofiber areas in longitudinal sections of EDL muscle from 8 M WT and GS mice were evaluated for: (1) the total number of mitochondria: (2) mitochondria positioning with respect to the A band of the sarcomere; (3) mitochondrial size (×10^−3^ nm^2^); and (4) the frequency of altered mitochondria. Mitochondria were classified as significantly altered when: (a) the external membrane was disrupted, (b) internal cristae were severely vacuolated, and/or (c) contained myelin figures.

#### Cryopreservation

Freshly isolated EDL, SOL, and *tibialis anterior* muscles were incubated in 30% sucrose solution for 8–24 h at 4 °C and then embedded with optimal cutting temperature compound (OCT) (Sakura Finetek USA). Embedded muscles were then flash frozen in isopentane at −55 °C and stored at −80 °C until use. For staining, 6–10 μm cross sections of flash frozen muscles were cut and mounted onto Superfrost Plus slides (ThermoFisher) using a Leica CM1800 cryostat (Leica Microsystems) at −20 °C.

#### Hematoxylin and eosin (H&E) and Gomori trichrome staining

6 μm EDL muscle cross sections were stained with Mayer’s H&E and Gomori trichrome. Images were captured using a Keyence BZ-X800 epifluorescence microscope (Keyence) at 40× (0.6-Air Plano Apochromat). Quantifications of the number of centrally nucleated fibers was performed manually by counting 300 fibers/condition from the H&E images.

#### Immunofluorescence

For fiber typing, mounted EDL and SOL cross sections (10 μm) were blocked for 30 min in blocking buffer (0.2% Triton X-100, 10% goat serum in phosphate-buffered saline [PBS] solution) and then incubated overnight at 4 °C with primary antibodies, followed by corresponding secondary antibodies for 1 h at room temperature the following day. The primary antibodies used were as follows: rabbit anti-laminin (1:500, Sigma-Aldrich), mouse anti-MHC1 (1:40, DSHB), mouse anti-MHC IIa (1:40, DSHB) and mouse anti-MHC IIb (1:40, DSHB). The secondary antibodies used were as follows: goat anti-mouse IgG1 555 (1:1000, ThermoFisher), goat anti-mouse IgM 488 (1:1500, ThermoFisher), goat anti-mouse IgG2b 350 (1:1500, ThermoFisher) and goat anti-rabbit IgG 647 (1:1500, ThermoFisher). Muscle sections were mounted with Fluoromount-G mounting media (Southern Biotech) and dried in the dark at room temperature. CSA and fiber type distribution were analyzed using SMASH software (Smith and Barton, 2014).

For ORAI1 localization, fresh EDL muscles from WT and *Orai1*^*V5HA/+*^ mice were incubated with 2% paraformaldehyde (PFA)/PBS solution for 2 h. Separated EDL bundles were then blocked with 10% normal goat serum and 2% X-Triton (Bio-Rad Laboratories) in PBS for another 2 h before incubating overnight at 4 °C with rabbit anti-HA (1:1000, Cell Signaling) and mouse anti-ORAI1 (1:1000, Santa Cruz Biotechnology) antibody. Bound antibodies were then detected by goat anti-rabbit IgG 488 (1:1500, ThermoFisher) and goat anti-mouse IgG 594 (1:1500, ThermoFisher) after 1.5 h incubation at room temperature. Muscle bundles were mounted with Fluoromount-G mounting media (Southern Biotech) and dried in the dark at room temperature. Images were taken using Nikon D-Eclipse C1 confocal microscope (Nikon) equipped with a 40×, NA 1.3 oil-immersion objective. Co-localization analysis was performed using ImageJ (NIH).

#### Grip strength

Mice were gently grabbed by their tail and allowed to grasp the metal grid-lined force transducer with their forelimbs. As soon as they grasped the grid, they were pulled gently away from the grid by their tail until the grip was lost. The peak force of each measurement was recorded (in mN). Grip strength of each mouse was measured in triplicate with a 10 min resting period between measurements. The average value from the three trials was normalized to their body mass to obtain normalized grip strength values (mN/g).

#### Treadmill endurance

Mice first underwent a 3-day acclimation training session at 5 m/min and 15° inclination to treadmill running (Columbus Instruments). On the following (fourth) day, mice were placed on the treadmill with an initial 5 min warmup phase at 5 m/min, 15°, followed by an incremental increase in treadmill speed of 1 m/min every minute until the treadmill reached a maximum speed of 20 m/min. This speed was then kept for the rest of the experiment. The entire treadmill endurance protocol lasted 60 min and covered a total of 1 km in distance. To ensure continuous running, when a mouse stopped, a brief burst of air lasting less than 1 s was delivered using a Whoosh Duster™ onto the backside of the mouse and was then counted as a 3 s rest. The cumulative number of rests during the protocol was recorded and binned for every 5 min.

#### Rotarod endurance

A 3-day acclimation training session for 20 min at 15 revolution per minute (rpm) on a Rotamex-5 (Columbus Instruments) was performed prior to the experimental session, as described previously (Wei-Lapierre et al, 2013). On the following (fourth) day, the trained mice were placed on the rotarod at an initial speed of 14 rpm with an increment of 1 rpm every 5 min until it reached a maximum speed of 23 rpm. Once the maximum speed was obtained, it was maintained until the 60 min protocol was completed. After each fall, the mouse was promptly placed back on the rotarod. During the experiment, the cumulative number of falls during the 60 min protocol were recorded and then binned for every 5 min.

#### Ex vivo muscle contractility

Mice were anesthetized as described above. Ex vivo assessment of muscle-specific force production and maintenance for intact EDL and SOL muscle was determined using an ASI muscle contraction system (Aurora Scientific) equipped with a 300C-LR dual mode force transducer and a 701 C stimulator. Muscles were continuously perfused with 37 °C ex vivo Ringer solution (137 mM NaCl, 5 mM KCl, 1.2 mM NaH_2_PO_4_, 1 mM MgSO_4_, 2 mM CaCl_2_, 10 mM glucose, and 24 mM NaHCO_3_, pH 7.4) and the optimal muscle length was determined using a series of 1 Hz stimulations. Stimulus output was set at 120% of the required voltage to elicit maximal muscle force. Tested muscles were first equilibrated using three tetani (500 ms, 150 Hz) with 1 min intervals to achieve its optimal performance. A standard force frequency protocol (from 1 to 250 Hz for EDL or 1 to 200 Hz for SOL muscles) was used to determine the frequency dependence of muscle force production. After a 5 min rest, muscles were stimulated with either a repetitive moderate-frequency protocol (60 consecutive, 500 ms duration, 50 Hz stimulus trains delivered every 2.5 s) or a sustained high-frequency protocol (150 Hz stimulus for 15 s or 30 s for EDL and SOL respectively). Muscle force was recorded and analyzed using DMC/DMA-HT software (Aurora Scientific). Physiologic CSA and specific force were calculated for each muscle, as described previously (Hakim et al, 2011).

#### Transfection of *Orai1* cDNA into HEK293 cells

HEK293 cells obtained from ATCC were maintained in Dulbecco’s Modified Eagle Medium (DMEM, ThermoFisher) supplemented with 10% fetal bovine serum (ThermoFisher), 2 mM glutamine, 30 units/mL penicillin and 30 μg/mL streptomycin. WT and/or mutant mouse *Orai1* cDNA constructs were transfected using Lipofectamine-2000 (ThermoFisher) according to manufacturer’s instruction.

#### Myotube cultures and fiber isolation

Myotubes differentiated from muscle stem cells isolated from limbs of newborn pups, muscle fibers from ribcages of embryos at embryonic day 14, and FDB fibers isolated from mice across multiple ages (from 2 weeks to 18 months) were obtained from WT and GS mice. For myofiber isolation, muscles were digested with 0.1% collagenase A for 30–75 min at 37 °C in Ringer’s solution (145 mM NaCl, 5 mM KCl, 2 mM CaCl_2_, 1 mM MgCl_2_, and 10 mM HEPES, pH 7.4), triturated using fine bore glass pipettes to mechanically dissociate fibers, and then plated onto glass coverslips for at least 20 min prior to single fiber experiments. Only fibers with healthy morphology, clear striations, and no signs of swelling or damage were used.

#### Mn^2+^ quench measurements

Transfected HEK293 cells, myotubes, embryonic myofibers, and acutely dissociated FDB fibers were loaded with 5 μM fura-2 AM (ThermoFisher) in a Ca^2+^-free Ringer’s solution (145 mM NaCl, 5 mM KCl, 3 mM MgCl_2_, and 0.2 mM EGTA, pH 7.4) for 1 h at 37 °C. A skeletal muscle myosin inhibitor (30 μM *N*-benzyl-*p*-toluene sulfonamide [BTS]) was included along with fura-2 to prevent muscle movement. In experiments measuring maximal SOCE activity, fibers were also incubated with a SERCA pump inhibitor cocktail (1 μM thapsigargin [TG] and 15 μM cyclopiazonic acid [CPA]) in addition to BTS and fura-2. For measurements of both constitutive Ca^2+^ entry (no store depletion) and SOCE (after store depletion), fibers were first rinsed with Ca^2+^-free Ringer’s solution at the end of the incubation period and then excited at 361 nm (isosbestic point of fura-2). Emission was detected at 510 nm using a DeltaRam illumination system (Photon Technology International). After obtaining an initial baseline rate of fura-2 decay, fibers were exposed to Ca^2+^-free Ringer supplemented with 0.5 mM MnCl_2_. The maximum rate of Ca^2+^ entry was calculated as the peak differential of the fura-2 emission trace during Mn^2+^ application (dF/dt in counts/s).

#### Resting intracellular Ca^2+^concentration

Single FDB fibers from 2 WK and 8 M old WT and GS mice were loaded with 4 μM fura-2 AM (ThermoFisher) at room temperature for 30 min followed by a 30-min washout in dye-free Ringer’s solution supplemented with 40 μM BTS. Fibers were alternatively excited at 340 nm and 380 nm (510 nm emission, 30 ms exposure per wavelength) and fura-2 ratio (ΔRatio_340/380_) was measured. Free Ca^2+^ concentration was calculated using an in-situ calibration approach described previously (Grynkiewicz et al, 1985) and the following equation:

$$[{{{{\rm{Ca}}}}}^{2+}]=428* ((\Delta {{{{\rm{Ratio}}}}}_{340/380}/1000)-0.219)/(2.126-(\Delta {{{{\rm{Ratio}}}}}_{340/380}/1000))* 6.39$$

#### Total releasable Ca^2+^ store content

The total releasable Ca^2+^ store content in FDB fibers from 2 WK and 8 M WT and GS mice was determined as previously described (Loy et al, 2011). In brief, fibers were loaded with 4 μM fura-FF AM (AAT Bioquest), a low-affinity ratiometric Ca^2+^ dye, at room temperature for 30 min followed by a 30-min washout in dye-free Ringer’s solution supplemented with 40 μM BTS. Fibers were alternatively excited at 340 nm and 380 nm (510 nm emission) while perfused with Ca^2+^-free Ringer solution for 40 s. After recording the baseline fura-FF ratio (ΔRatio_340/380_), fibers were then perfused with a Ca^2+^ release cocktail (ICE, 10 μM ionomycin, 30 μM cyclopiazonic acid and 100 μM EGTA/0 Ca^2+^) that releases Ca^2+^ from all intracellular Ca^2+^ storage compartments. Ca^2+^-containing Ringer solution was added at the end of the experiment to confirm that fura-FF was not saturated during ICE application. Total releasable Ca^2+^ store content was determined as the difference between baseline and peak ratio divided by baseline ratio (ΔR/R_0_).

#### Western blot analyses

Skeletal muscles and other organs (brain, heart, liver, lung, kidney, pancreas, spleen, submandibular gland [SMG]) from 2 WK and 8 M old WT and GS mice were homogenized in ice-cold RIPA lysis buffer and centrifuged for 30 min at 13,000 rpm at 4 °C. For protein deglycosylation experiments, 20 μg of lysate was treated with 1000U PNGase F (New England Biolab) overnight at 37 °C. Protein concentrations were determined using RC DC Protein Assay (Bio-Rad Laboratories). In total, 10–50 μg of protein were loaded per lane and separated with 10% or 12% SDS-PAGE gels, transferred onto nitrocellulose membranes (Bio-Rad Bioscience), stained with Ponceau S (Sigma-Aldrich) for total protein normalization, and then blotted with different primary antibodies followed by appropriate secondary antibodies. The primary used were rabbit anti-ORAI1 (1:1000, AbClonal), rabbit anti-ORAI1 (1:500, ThermoFisher), mouse anti-ORAI1 (1:500, Santa Cruz), rabbit anti-ORAI1 (1:1000, generated by Pocono Rabbit Farm Lab), rat anti-HA (1:4000, Sigma-Aldrich), rabbit anti-STIM1 (1:3000, Sigma-Aldrich), mouse anti-CASQ1 (1:1000, Thermofisher), mouse anti-SERCA1/2/3 (1:1000, Santa Cruz), mouse anti-PMCA (1:2000, Thermofisher), mouse anti-NCX (1:1000, Swant) or rabbit anti-VDAC (1:5000, Sigma-Aldrich). The secondary antibodies used were HRP-conjugated goat anti-rat (1:10,000, Abcam), anti-rabbit IgG-HRP (1:10,000, Kindle Bioscience), anti-mouse IgG-HRP (1:5000, Kindle Bioscience), goat anti-mouse IgG DyLight 800 (1:10,000, Thermofisher) and goat anti-rabbit IgG DyLight 800 (1:10,000, Thermofisher). Images were taken using either a LI-COR system (for fluorescent images, LI-COR Biosciences) or digitally with multiple exposure times (for HRP images) after incubation with ECL substrate (Kindle Bioscience) on the Kwik Quant digital imager (Kindle Bioscience). Protein expression levels were normalized to Ponceau S total protein staining and analyzed using Image Studio (LI-COR Biosciences) and/or ImageJ (NIH).

#### Immunoprecipitation

Overall, 300 μg of *tibialis anterior* or spleen lysates in RIPA buffer were incubated with 1 μg of either the rabbit anti-HA (Cell Signaling) or an N-terminal rabbit anti-ORAI1 rabbit antibody (generated by Pocono Rabbit Farm Lab) in a total volume of 500 μL. After 2 h of incubation at 4 °C while shaking, 15 μL of magnetic Protein A/G beads (ThermoFisher) were added to the immunoprecipitation and the samples were then incubated overnight at 4 °C with shaking. Following the overnight incubation, beads were separated on a magnetic rack and washed twice with 500 μL RIPA lysis buffer followed by one wash with PBS. Washed beads were resuspended in 60 μL of sample loading buffer and incubated at 37 °C for 15 min. 15 μL of the IP was run per lane on a 12% SDS-PAGE gel. After transfer, the membrane was blotted with rat anti-HA primary antibody (1:4000, Sigma-Aldrich) and HRP-conjugated goat anti-rat secondary antibody (1:10,000, Abcam). Images were taken digitally with multiple exposure times after incubation with ECL substrate (Kindle Bioscience) on the Kwik Quant digital imager (Kindle Bioscience).

#### Quantitative RT-PCR

Total RNA from snap-frozen *tibialis anterior* muscles of 2 WK and 8 M old WT and GS mice was isolated using Trizol reagent (ThermoFisher) according to manufacturer’s protocol and then purified using an RNAse mini kit (Qiagen). RNA (1 µg) was mixed with DNase I (ThermoFisher), Super Script III (ThermoFisher), dNTPs (ThermoFisher), and oligo(dT) (ThermoFisher) as previously described (Maani et al, 2018). RT-qPCR was performed on a StepOne Plus Real-Time PCR machine (Applied Biosystems) using SYBR Green FastMix (Quantabio). Reference genes, *Gapdh* and/or *Rpl7l1*, were used as loading controls. Mitochondrial transcript levels were assessed using the mitochondrial specific *16S rRNA*. *Orai1* primers were designed using regions that were not altered by the edited G100S mutation. All primer sequences are summarized in Table 3. mRNA levels were analyzed using the 2^-∆∆CT^ method as previously described (Schmittgen and Livak, 2008).

**Table 3:** Summary of primer sequences used in RT-qPCR.

*Gapdh*	Forward sequence	5’-AGGAGAGTGTTTCCTCGTCC-3’
	Reverse sequence	5’-TGAGGTCAATGAAGGGGTCG-3’
*Rpl7l1*	Forward sequence	5’-ACGGTGGAGCCTTATGTGAC-3’
	Reverse sequence	5’-TCCGTCAGAGGGACTGTCT-3’
*Orai1*	Forward sequence	5’-GATCGGCCAGAGTTACTCCG-3’
	Reverse sequence	5’-TGGGTAGTCATGGTCTGTGTC-3’
*16S rRNA*	Forward sequence	5’-CCGCAAGGGAAAGATGAAAGAC-3’
	Reverse sequence	5’-TCGTTTGGTTTCGGGGTTTC-3’

#### Generation of mitochondrial-targeted YC3.60

The following sequence that includes two copies of the Cox8 (represented in bold) sequence and one copy of the Cox4 (represented in italic) sequence was generated by PCR in a vector containing the CMV enhancer and the chicken β-actin promoter (2xCox8-Cox4):

**MSVLTPLLLRGLTGSARRLPVPRAK**IHSLGDL**SVLTPLLLRGLTGSARRLPVPRAK**IHSLGDPPVAPAGASMTGGQQMGRDLYDDDDKDP*MLSLRQSIRFFK*RSGI

The cDNA for the YC3.60 Ca^2+^cameleon probe (Addgene plasmid # 67899; pYC3.60-C1) was cloned in-frame to this 2xCox8-Cox4 sequence between the Nhe1 and EcoR1 sites to generate the final mitochondrial-targeted YC3.60 construct (mt-YC3.6).

#### Activity-dependent mitochondrial Ca^2+^ uptake

In all, 8–12 M old WT and GS mice were electroporated with mt-YC3.6 cDNA, as previously described (Ainbinder et al, 2015; DiFranco et al, 2009; Shaw and Feske, 2012). Briefly, in vivo electroporation into mouse hind paws was carried out by first injecting 2 mg/mL hyaluronidase followed by 10 µL of plasmid suspension into each hind paw. Twenty brief electrical pulses (10 V, 20 ms, 1 Hz) were delivered to the hind paws using a pair of needles inserted under the skin. After 10 days, FDB muscles were excised and digested with 1 mg/mL collagenase at 37 °C and individual fibers were mechanically separated with gentle trituration using 3 sequentially smaller gauge glass pipettes. Activity-dependent mitochondrial Ca^2+^ uptake was initiated using a repetitive stimulation paradigm (300 pulses, 50 Hz, 500 ms, every 2.5 s). YC3.6 fluorescence was continuously captured each stimulation using an epifluorescence illumination system with a 40× oil immersion lens. Fibers were excited at 458 nm with emission collected at 406–505 nm (CFP) and 525–535 nm (YFP) using a photomultiplier detection system (Chroma Technologies) and Clampfit software (Molecular Devices). All mitochondrial Ca^2+^ uptake studies were conducted at room temperature.

#### Mass spectrometry

Mass spectrometry of* tibialis anterior* muscles from 2 WK and 8 M old WT and GS mice was performed by the University of Rochester Mass Spectrometry Resource Laboratory. In brief, snap-frozen *tibialis anterior* muscles were homogenized, sonicated, and quantified using a bicinchoninic (BCA) assay (ThermoFisher). Collected supernatants were then denatured, reduced, alkylated, cleaned, and digested using S-Trap micro columns (Protifi). Flowthrough was frozen, dried in a Speed Vac (Labconco), and the remaining precipitates were resuspended in 0.1% Trifluoroacetic (TFA) prior to mass spectrometry analysis. An Easy nLC-1200 HPLC (ThermoFisher) connected to a Fusion Lumos Tribrid mass spectrometer (ThermoFisher) was used for mass spectrometry measurement, while a Nanospray Flex source operating at 2 kV was used to introduce ions.

Raw data was processed using DIA-NN version 1.8.1 in library-free analysis mode (Demichev et al, 2020). Library annotation was conducted using the mouse UniProt ‘one protein sequence per gene’ database (UP000000589_10090) with deep learning-based spectra and RT prediction enabled. Only significantly different (*P* < 0.05) proteins within the top three quartiles of mean peptide abundance were included in the final analyses. Gene Ontology (GO) Cellular Component and network analyses were conducted using ShinyGO 0.80 (South Dakota State University) (Ge et al, 2020; Luo and Brouwer, 2013). The false discovery rate (FDR) cutoff was set at 0.05 and pathways were sorted by -Log10(FDR). Volcano plots were generated using the web-based software VolcaNoseR (Goedhart and Luijsterburg, 2020). Volcano plot significance thresholds were set at -Log10 > 1.3 and the Log2 fold change threshold was set to exclude values between −0.6 and 0.6. Pie charts summarizing significantly upregulated and downregulated proteins were plotted using GraphPad Prism 7 (GraphPad Software Inc.).

#### Mitochondrial membrane potential measurements

FDB fibers from 8 M old WT and GS mice were isolated as described above. Single fibers were incubated with 20 nM tetramethylrhodamine methyl ester (TMRE, mitochondrial membrane potential indicator) for 30 min at room temperature. To monitor TMRE fluorescence, a time series of images was acquired using a Nikon Eclipse C1 Plus confocal microscope (Nikon) equipped with a 40× oil objective and 543 nm laser. Emission was detected at 605 nm and sequential images were taken at 15 s intervals with a total of 40 frames. In all, 60 s after the start of the time series, 1 µM FCCP was perfused onto the fiber while the decay of TMRE fluorescence was continually monitored.

#### Mitochondrial superoxide measurements

Isolated FDB fibers from 8 M old WT and GS mice were treated for 15 min with 5 μM MitoSOX Red (ThermoFisher) in Tyrode’s solution (34.25 mM NaCl, 5 mM KCl, 1.8 mM CaCl_2_, 1 mM MgCl_2_, 400 μM NaH_2_PO_4_, 100 μM EDTA, 5.5 mM glucose and 24 mM NaHCO_3_, pH 7.4), followed by a 15 min incubation with dye-free Tyrode’s solution. MitoSOX Red is a mitochondrial-targeted superoxide indicator in which its fluorescence intensity increases with time as mitochondrial reactive oxygen species accumulate. All MitoSOX experiments were conducted using identical loading, excitation, and emission collection conditions. MitoSOX fluorescence was measured using a Nikon Eclipse C1 Plus confocal microscope (Nikon) with excitation/emission set at 488/605 nm. For each fiber, the fluorescence intensity at time 0 (T_0_) was measured immediately after 10 min of dye-free incubation with a second image taken exactly 10 min later with its fluorescence level recorded as T_10_. The rate of mitochondrial superoxide production was calculated as (T_10_ − T_0_)/T_0_.

#### Purification of mitochondria from skeletal muscle

8 M old WT and GS mice were sacrificed and their EDL, SOL, *gastrocnemius*, and *quadricep* muscles from both limbs were immediately excised and homogenized in extraction buffer (0.32 M sucrose, 10 mM Tris-HCl, 1 mM EDTA). Mitochondria were purified as previously described (Sanchez-Gonzalez and Formentini, 2021). In brief, muscle lysates were spun twice at 700 rpm for 10 min at 4 °C. The supernatant was collected and spun at 10,000 rpm for 10 min at 4 °C. The new supernatant was poured off, and the resulting pellet was resuspended in ~100 μL of respiration buffer (225 mM sucrose, 10 mM KCl, 5 mM MgCl_2_, 10 mM HK_2_PO_4_, 10 mM H_2_KPO_4_, 1 mM EGTA, 10 mM Tris-HCl, 0.05% BSA). For each sample, 5 μL was immediately used for oxygen consumption measurements, while the rest of the resuspended mitochondria were stored at −80 °C for subsequent complex I–V enzyme assays. Protein concentrations of purified mitochondria samples were determined using RC DC Protein Assay (Bio-Rad Laboratories).

#### ETC enzyme functional assays

Purified mitochondria from 8 M old WT and GS mice were used for all electron transport chain enzyme activity assays. Assays for complexes I through V were performed according to manufacturers’ instructions (Abcam, ab109721, ab109908, ab287844, and ab109911; Cayman Chemical, No. 701000).

#### Respiratory control ratio

Oxygen consumption was measured using an Oxytherm+ Clark-type oxygen electrode (Hansatech) with traces recorded/analyzed using the Oxytrace+ software (Hansatech). For each experiment, 500 μL of respiration buffer was added to the chamber. After 30 s of steady oxygen level recording, 5 μL of purified mitochondria was added. After the oxygen reading again stabilized, the following reagents were added in succession: 10 mM glutamate/malate (State II), 125 μM ADP (State III), 5 μM oligomycin (State IV), 5 μM FCCP (State V). The maximal slopes of oxygen consumption during each phase were measured. The respiration control ratio (RCR) was calculated by dividing the slope after the addition of ADP (State III) by the slope after the addition of oligomycin (State IV).

### Statistical analyses

Experiments were performed blinded for behavioral tests (grip strength, rotarod and treadmill test), hematology tests, immunofluorescence staining and proteomics. All results were reported as mean ± SEM. Chi-square test was used to calculate the statistical significance in the deviation from Mendelian birth ratio in the mouse line. For continuous data, Shapiro–Wilk test and *F* test were always performed before statistical analysis to check normality and homoscedasticity. For comparisons between two groups, if data are not normally distributed, a Mann–Whitney test was performed. If data pass the normality test but exhibit different variance, a Welch’s *t* test was carried out. For data that pass both normality and equal variance test, a Student’s *t* test was performed. For experiment with 3 or more groups, statistical significance was determined using one-way analysis of variance (ANOVA), followed by Holm–Sidak’s post hoc test for multiple comparisons. For experiments with 2 variables, two-way ANOVA was used, again followed by Holm–Sidak’s post hoc test for multiple comparisons. GraphPad Prism 7 (GraphPad Software Inc.) was used for all statistical analyses and to graph plots. *P* < 0.05 or FDR < 1% was considered statistically significant. Individual data points in each bar graph always represent biological replicates unless specified in the legend.

## Supplementary information

Appendix

Peer Review File

Source data Fig. 1

Source data Fig. 2

Source data Fig. 3

Source data Fig. 4

Source data Fig. 5

Source data Fig. 7

Expanded View Figures

## Data Availability

The mass spectrometry proteomics were deposited in the ProteomeXchange Consortium via the PRIDE partner repository (Perez-Riverol et al, 2022) with the dataset identifier PXD053472: https://www.ebi.ac.uk/pride/archive/projects/PXD053472/privatereviewdataset. The source data of this paper are collected in the following database record: biostudies:S-SCDT-10_1038-S44318-024-00273-4.
