# Supplementary material for: An Orai1 gain-of-function tubular aggregate myopathy mouse model phenocopies key features of the human disease
Source: EMBO J. 2024 Oct 17;43(23):5941–71. doi: 10.1038/s44318-024-00273-4 (PMC11612304; doi:10.1038/s44318-024-00273-4)
Supplement: Supplementary file 1 — Appendix [file 44318_2024_273_MOESM1_ESM.pdf]

Appendix for

An *Orai1* gain-of-function tubular aggregate myopathy mouse model phenocopies key features of the human disease

Table of Contents

p2: Appendix Figure S1: Whole blood complete blood count analysis of 8M old WT (n=8) and GS (n=6) mice.

p3: Appendix Figure S2: *Orai1* transcript level in GS mice.

p4: Appendix Figure S3: ORAI1 subcellular localization in muscle fibers from 8M old V5HA/+ and V5HA/GS mice.

p5: Appendix Figure S4: Mitochondrial Ca^{2+} uptake/efflux during repetitive high frequency stimulation.

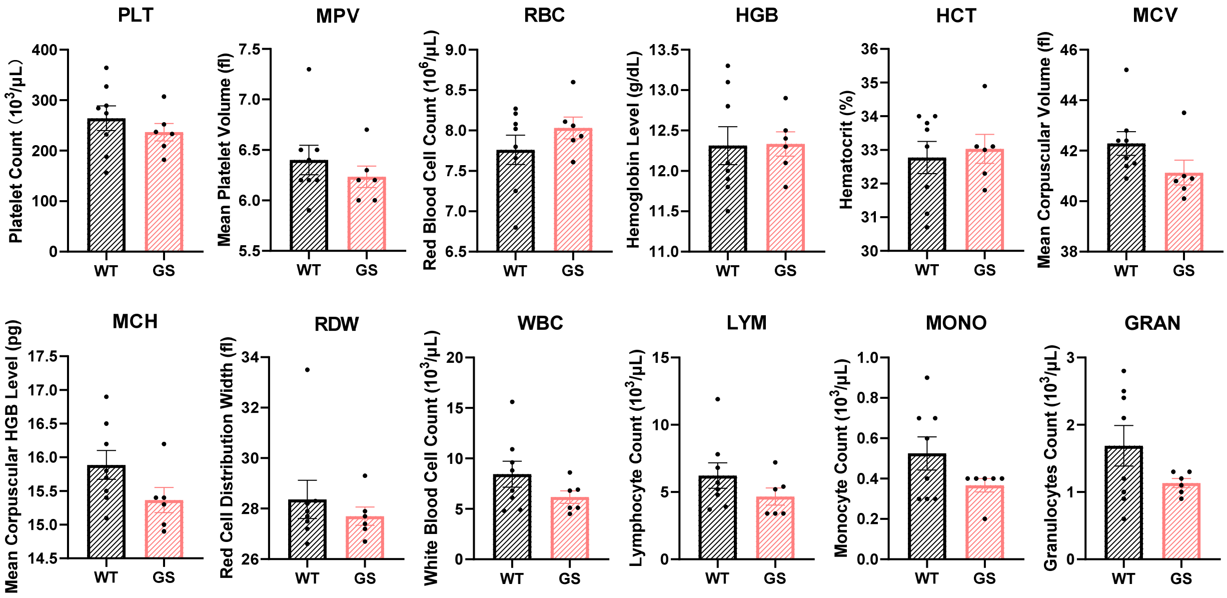

Appendix Figure S1. Whole blood complete blood count analysis of 8M old WT (n=8) and GS (n=6) mice.

PLT: platelet count; MPV: mean platelet volume; RBC: red blood cell; HGB: hemoglobin; HCT: hematocrit; MCV: mean corpuscular volume; MCH: mean corpuscular hemoglobin; RDW: red cell distribution width; WBC: white blood cell; LYM: lymphocyte; MONO: monocyte; GRAN: granulocytes.

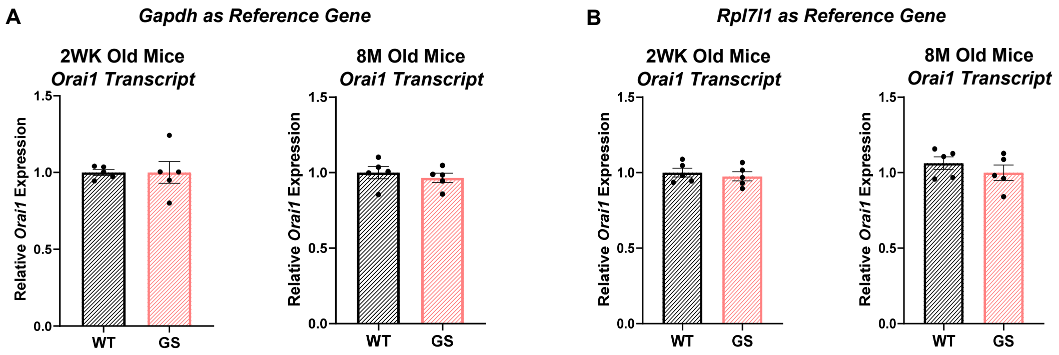

Appendix Figure S2. *Orai1* transcript level in GS mice.

A) *Orai1* transcript level normalized to *Gapdh* as a reference gene in *tibialis anterior* muscle from 2WK old (*left*) and 8M old (*right*) WT and GS mice. n=5 for all groups.

B) *Orai1* transcript level normalized to *Rpl7l1* as a reference gene in *tibialis anterior* muscle from 2WK old (*left*) and 8M old (*right*) WT and GS mice. n=5 for all groups.

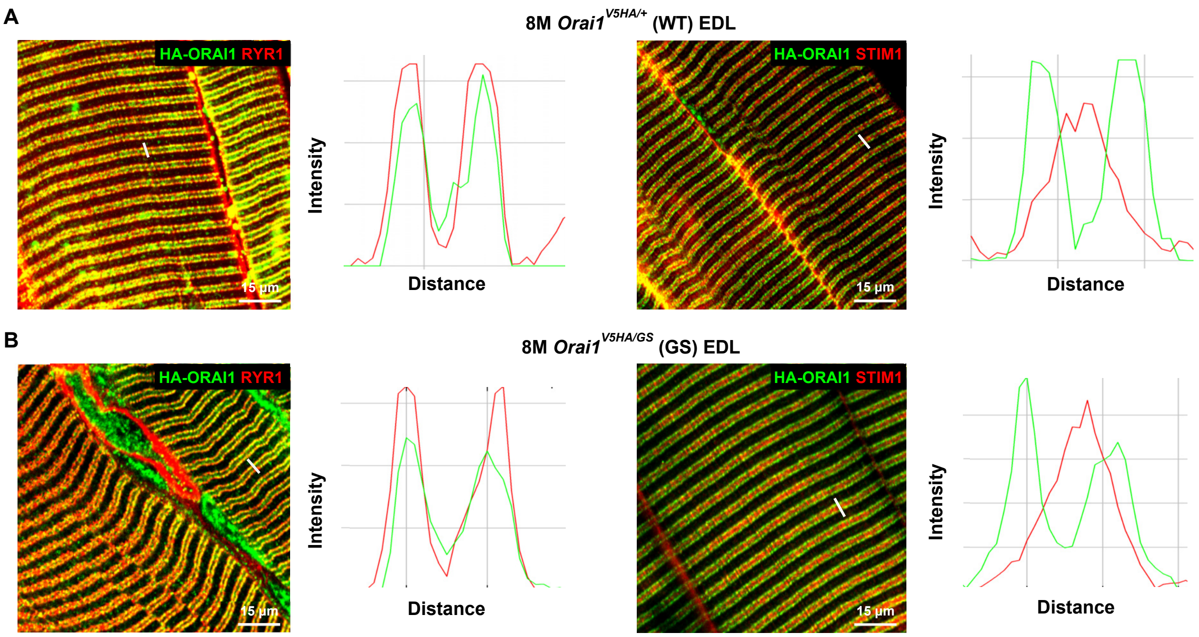

Appendix Figure S3. ORAI1 subcellular localization in muscle fibers from 8M old V5HA/+ and V5HA/GS mice.

A) Representative confocal image of EDL muscle bundle fibers from 8M old WT V5HA/+ mice co-stained with either ORAI1 and RYR1 (*left*) or ORAI1 and STIM1 (*right*) antibodies. Intensity profiles of ORAI1 (green) and either RYR1 (red) or STIM1 (red) across a single sarcomere (noted by white line in the corresponding image) are shown to the right of each image.

B) Representative confocal image of EDL muscle bundle fibers from 8M old V5HA/GS mice co-stained with either ORAI1 and RYR1 (*left*) or ORAI1 and STIM1 (*right*) antibodies. Intensity profiles of ORAI1 (green) and either RYR1 (red) or STIM1 (red) across a single sarcomere (noted by white line in the corresponding image) are shown to the right of each image.

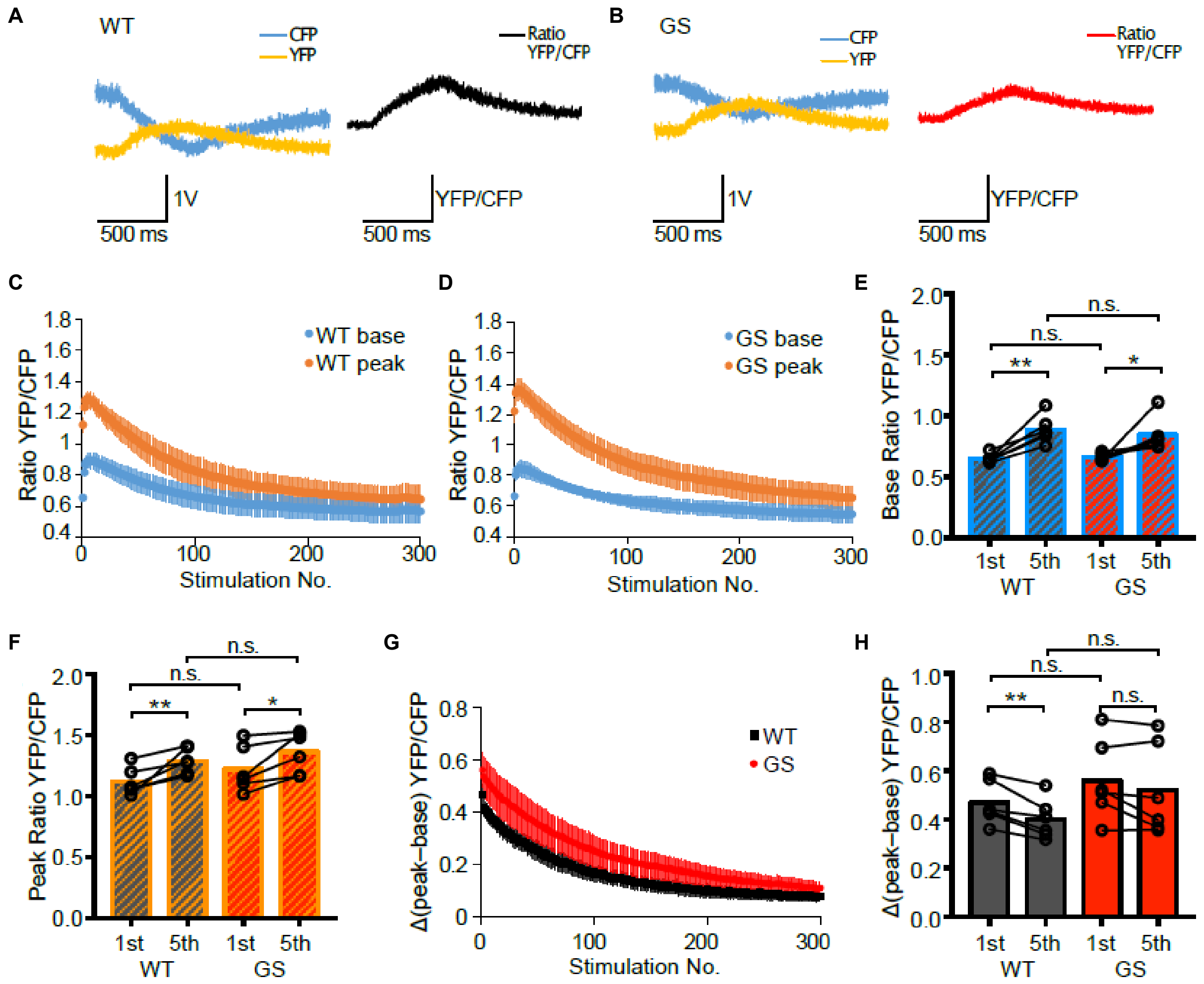

Appendix Figure S4. Mitochondrial Ca^{2+} uptake/efflux during repetitive high frequency stimulation.

A-B) Representative raw mt-YC3.6 fluorescence traces in FDB fibers isolated from 8M old WT (A) or GS (B) mice electroporated with mt-YC3.6. CFP (cyan line) and YFP (yellow line) emission signals were simultaneously recorded and used to calculate YFP/CFP ratio (black, WT or red, GS lines).

C and D) Average (±SEM) mt-YC3.6 ratio responses during repetitive electrical stimulation (50 Hz, 500 ms, every 2.5 sec) in FDB fibers from 8M old WT (C) and GS (D) mice. Baseline ratios just prior to each stimulation are presented in blue and peak ratios during each stimulation are presented in orange.

E and F) Summary histograms of baseline (E) and peak (F) ratio values after the first and fifth stimulation trains. A similar increase in baseline and peak ratios were observed from the first to fifth stimulation for both genotypes. . * $p < 0.05$; ** $p < 0.01$.

G) Summary of the difference in peak and baseline ratios during each stimulus train in FDB fibers from WT (black) and GS (red) mice.

H) Summary histograms of the difference in peak and baseline ratios from the first to fifth stimulation trains. n = 6 for each condition. ** $p < 0.01$.
