# Supplementary figures and images for: An Orai1 gain-of-function tubular aggregate myopathy mouse model phenocopies key features of the human disease
Source: EMBO J. 2024 Oct 17;43(23):5941–71. doi: 10.1038/s44318-024-00273-4 (PMC11612304; doi:10.1038/s44318-024-00273-4)

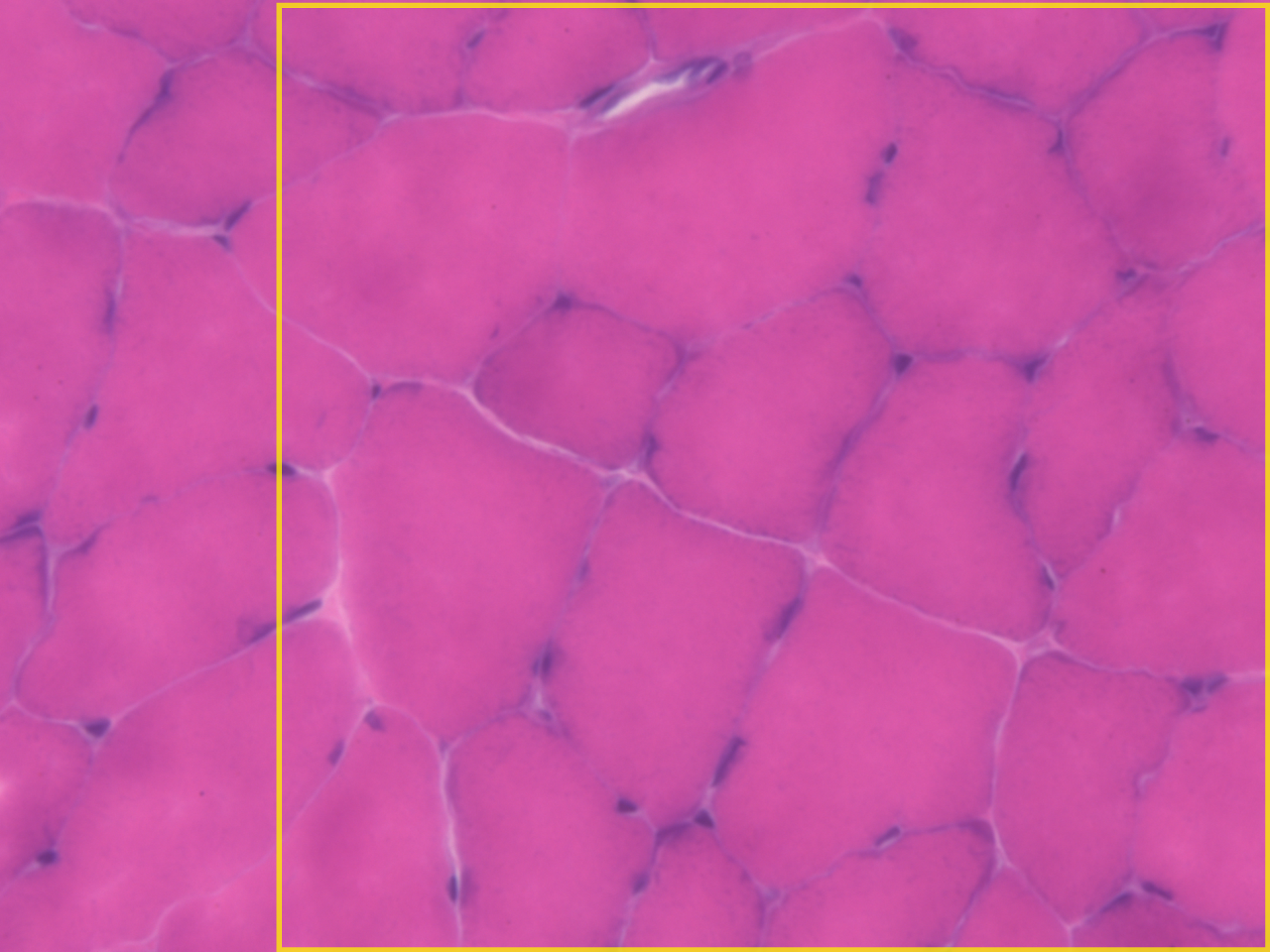

Supplement: Supplementary file 3 — Source data Fig. 1 [file 44318_2024_273_MOESM3_ESM.zip › Figure 1/1F/Raw Representitve image for 8M GS.tif]

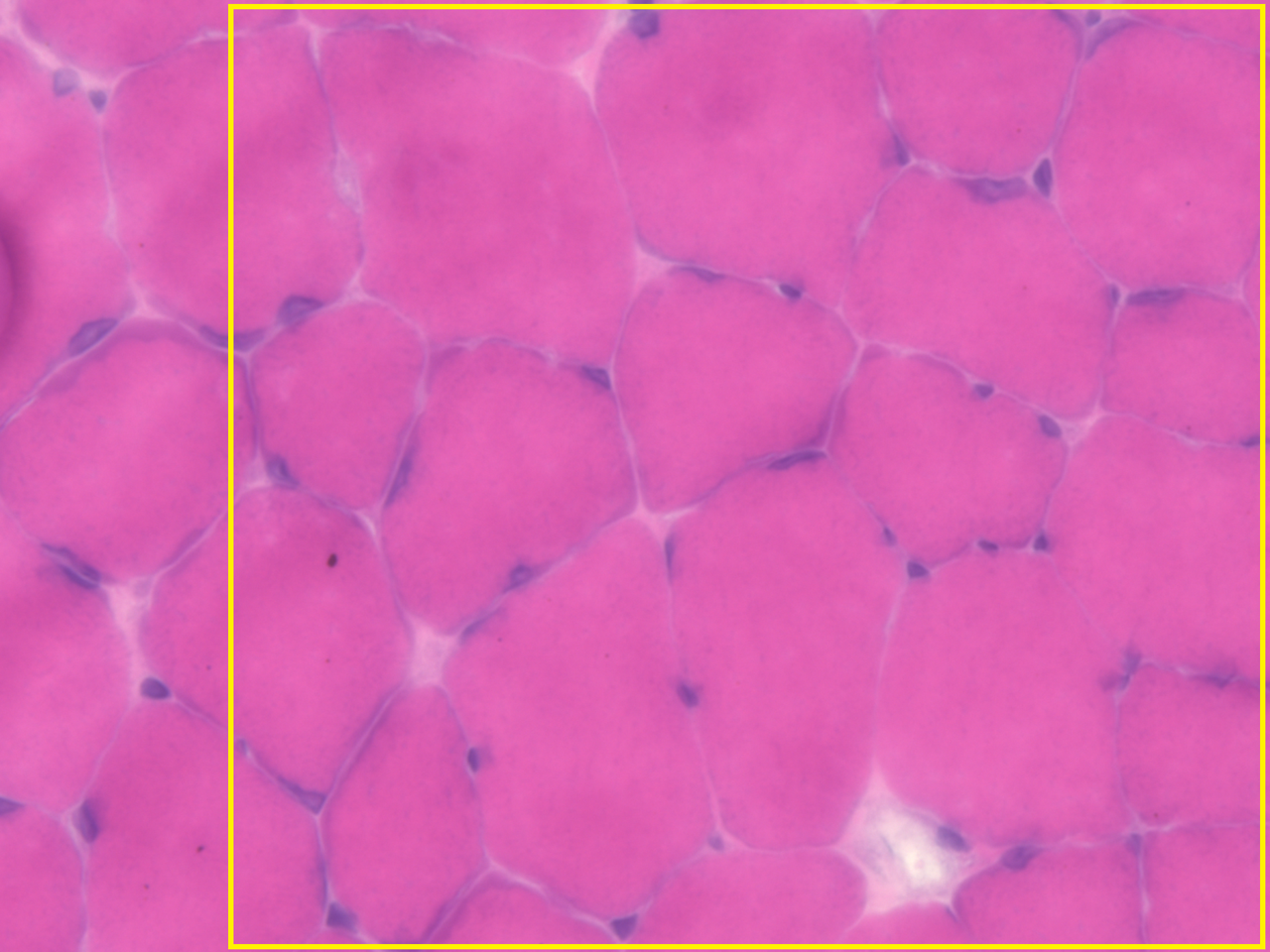

Supplement: Supplementary file 3 — Source data Fig. 1 [file 44318_2024_273_MOESM3_ESM.zip › Figure 1/1F/Raw Representitve image for 8M WT.tif]

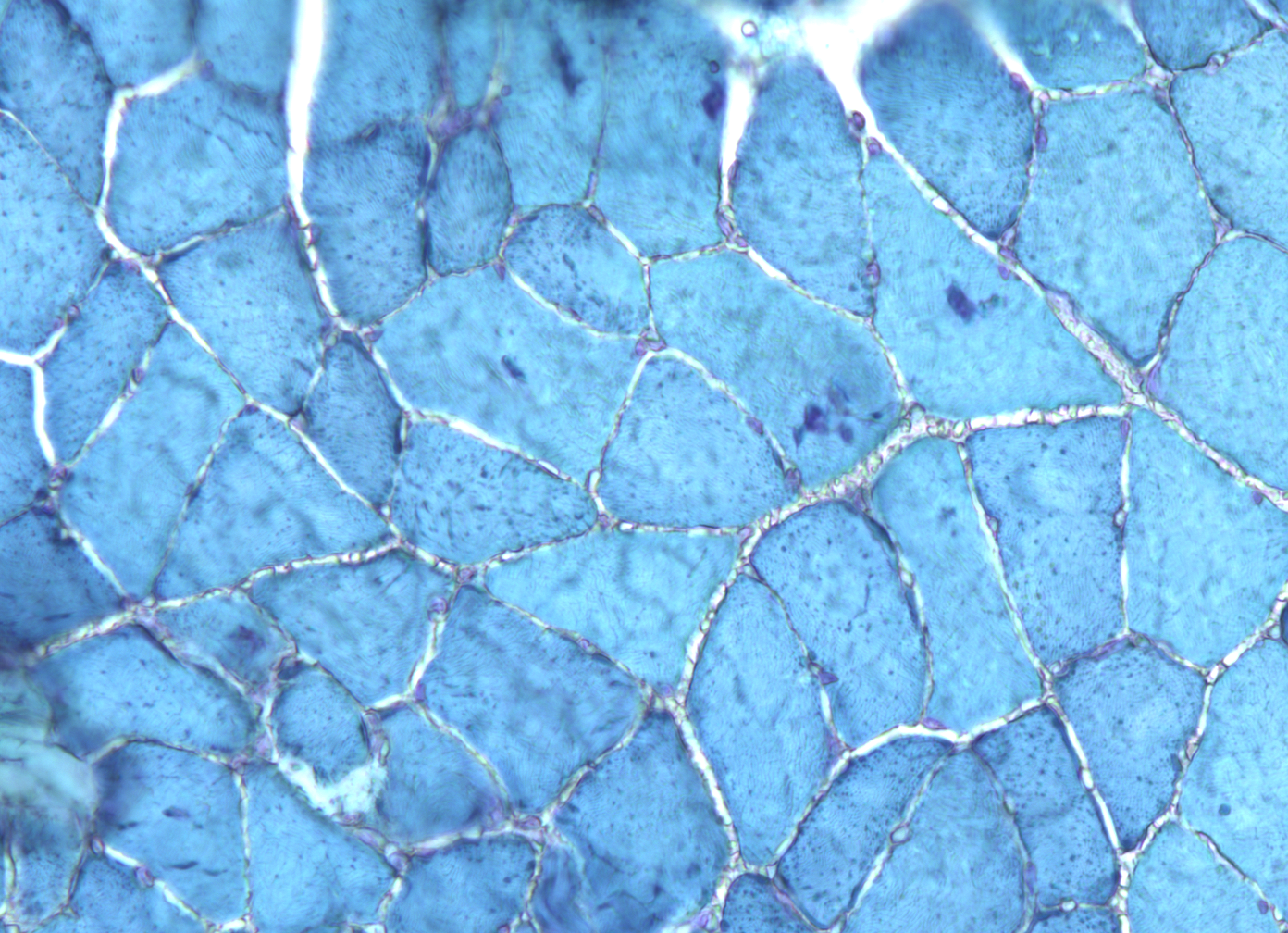

Supplement: Supplementary file 4 — Source data Fig. 2 [file 44318_2024_273_MOESM4_ESM.zip › Figure 2/2A/12M GS GT.tif]

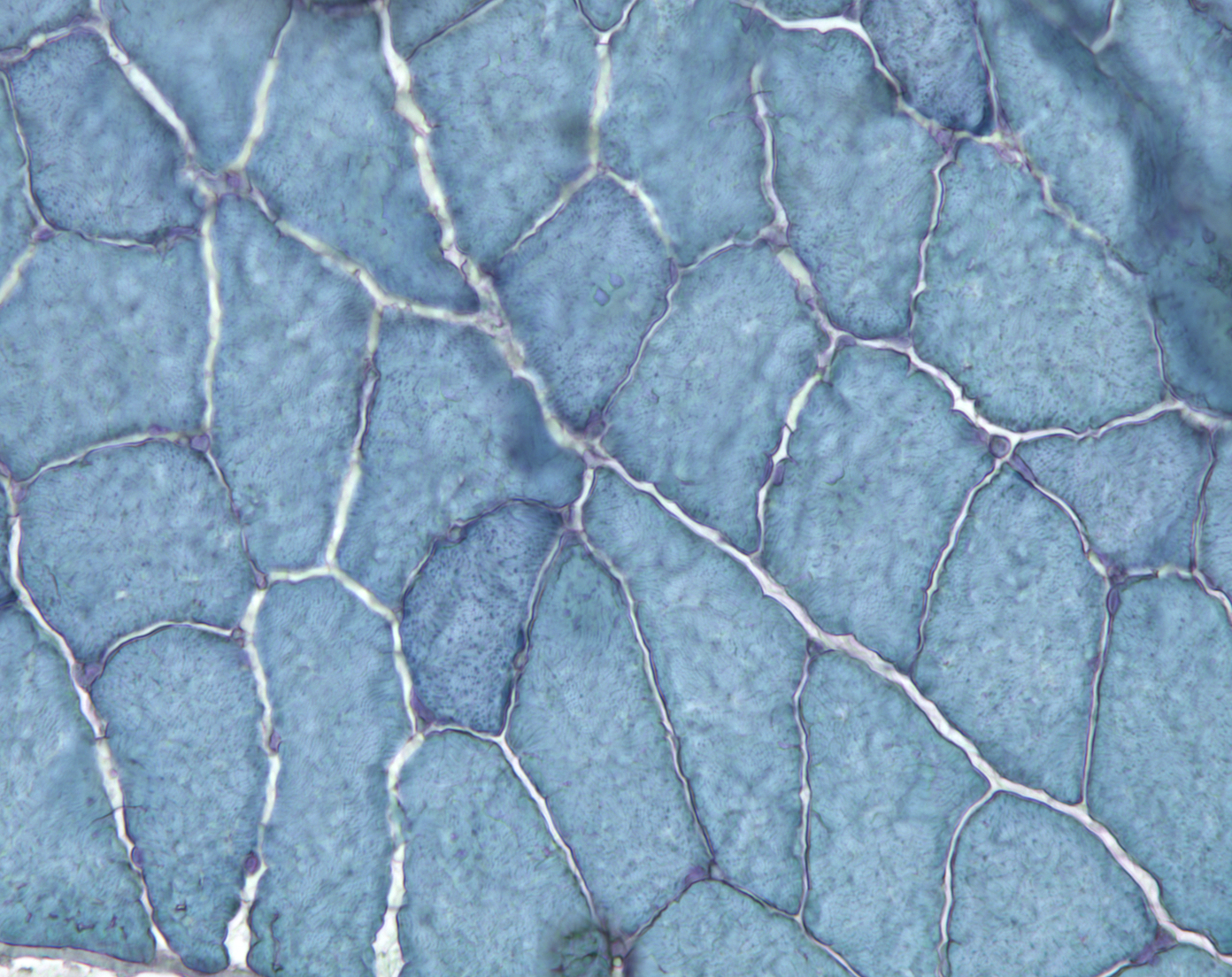

Supplement: Supplementary file 4 — Source data Fig. 2 [file 44318_2024_273_MOESM4_ESM.zip › Figure 2/2A/12M WT GS.tif]

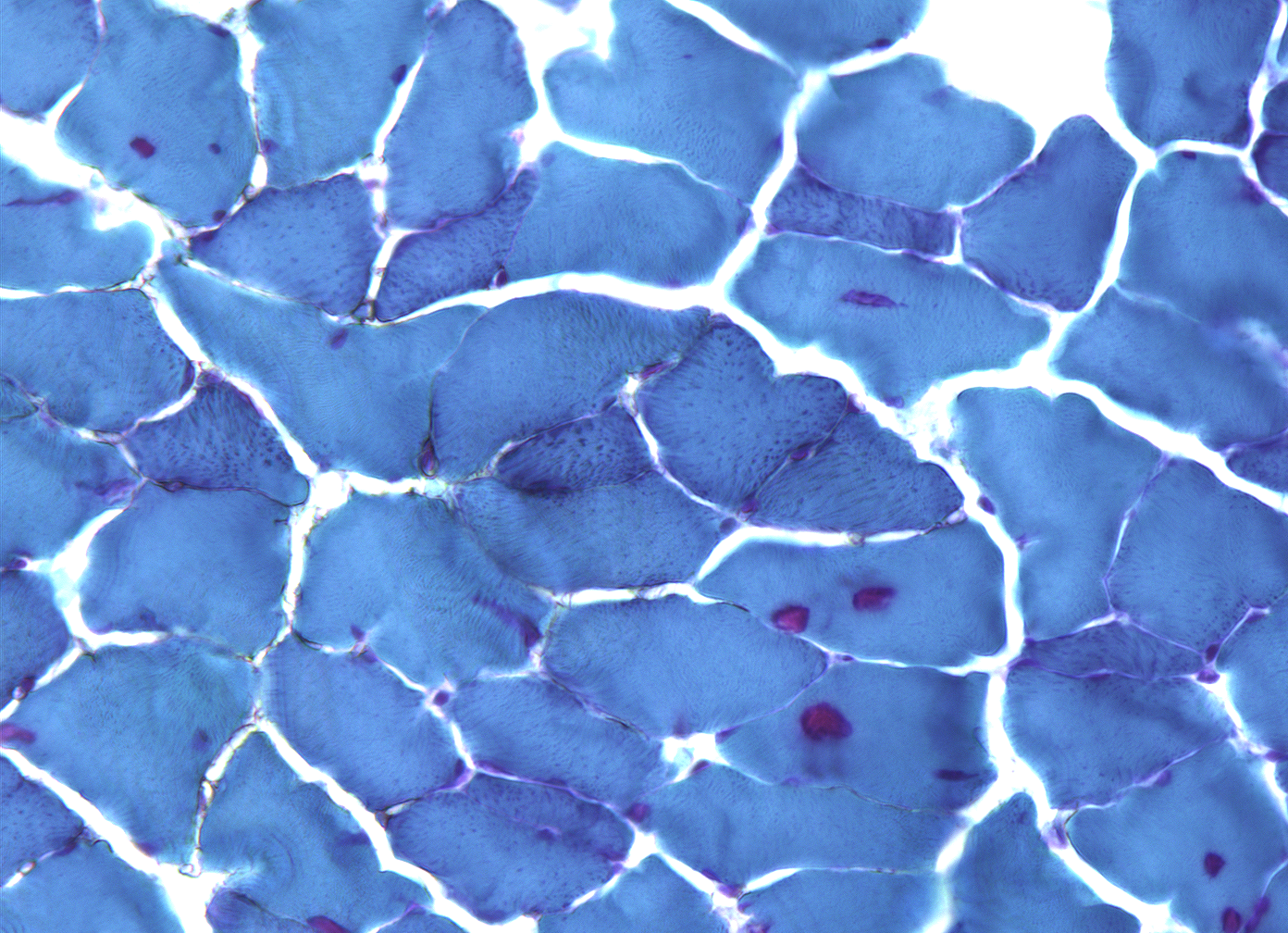

Supplement: Supplementary file 4 — Source data Fig. 2 [file 44318_2024_273_MOESM4_ESM.zip › Figure 2/2B/18M GS GT.tif]

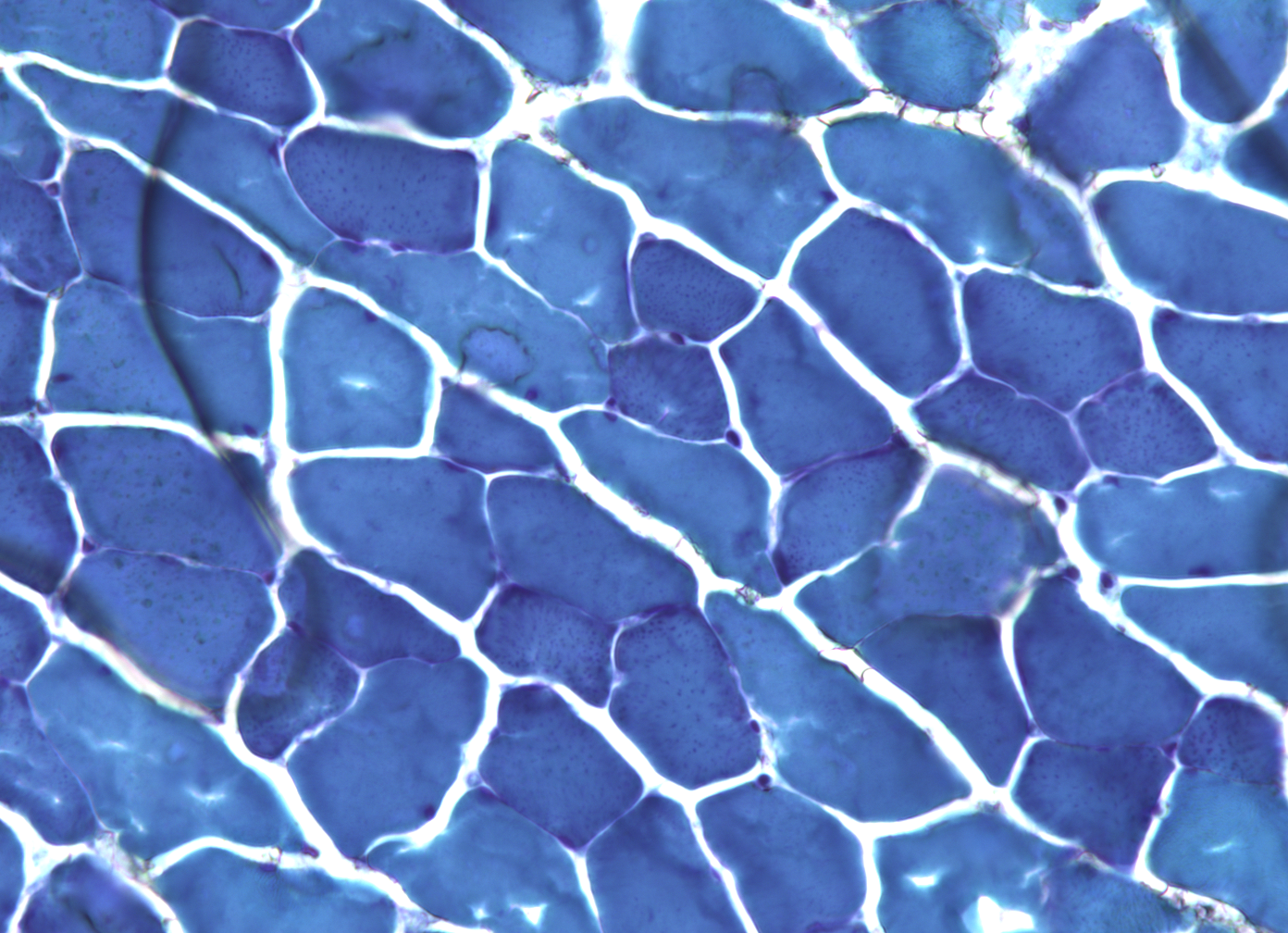

Supplement: Supplementary file 4 — Source data Fig. 2 [file 44318_2024_273_MOESM4_ESM.zip › Figure 2/2B/18M WT GT.tif]

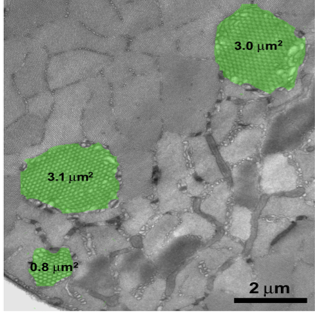

Supplement: Supplementary file 4 — Source data Fig. 2 [file 44318_2024_273_MOESM4_ESM.zip › Figure 2/2C-D/Representative EM image for 8M GS EDL.tif]

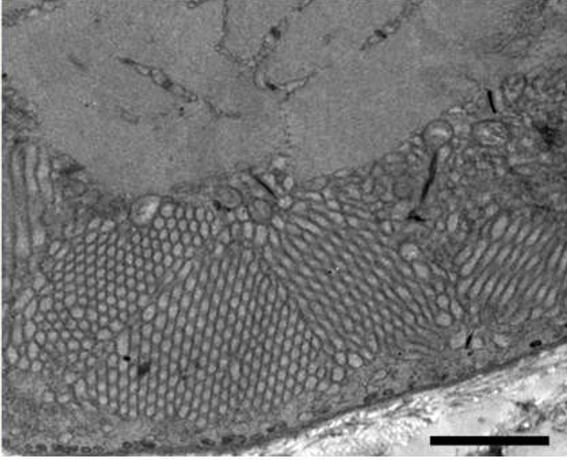

Supplement: Supplementary file 4 — Source data Fig. 2 [file 44318_2024_273_MOESM4_ESM.zip › Figure 2/2C-D/Representative EM image for 8M GS FDB.tif]

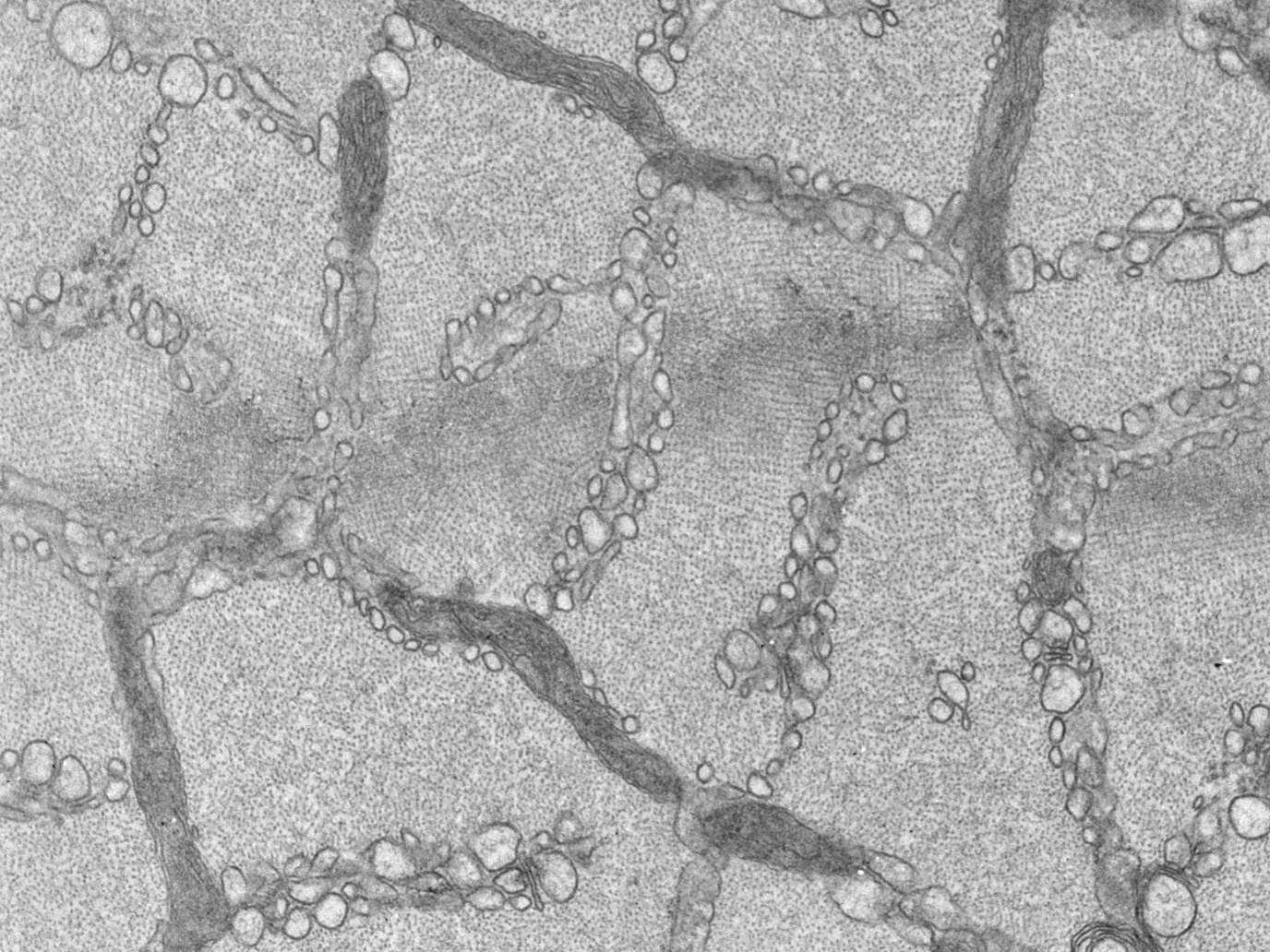

Supplement: Supplementary file 4 — Source data Fig. 2 [file 44318_2024_273_MOESM4_ESM.zip › Figure 2/2C-D/Representative EM image for 8M WT EDL.tif]

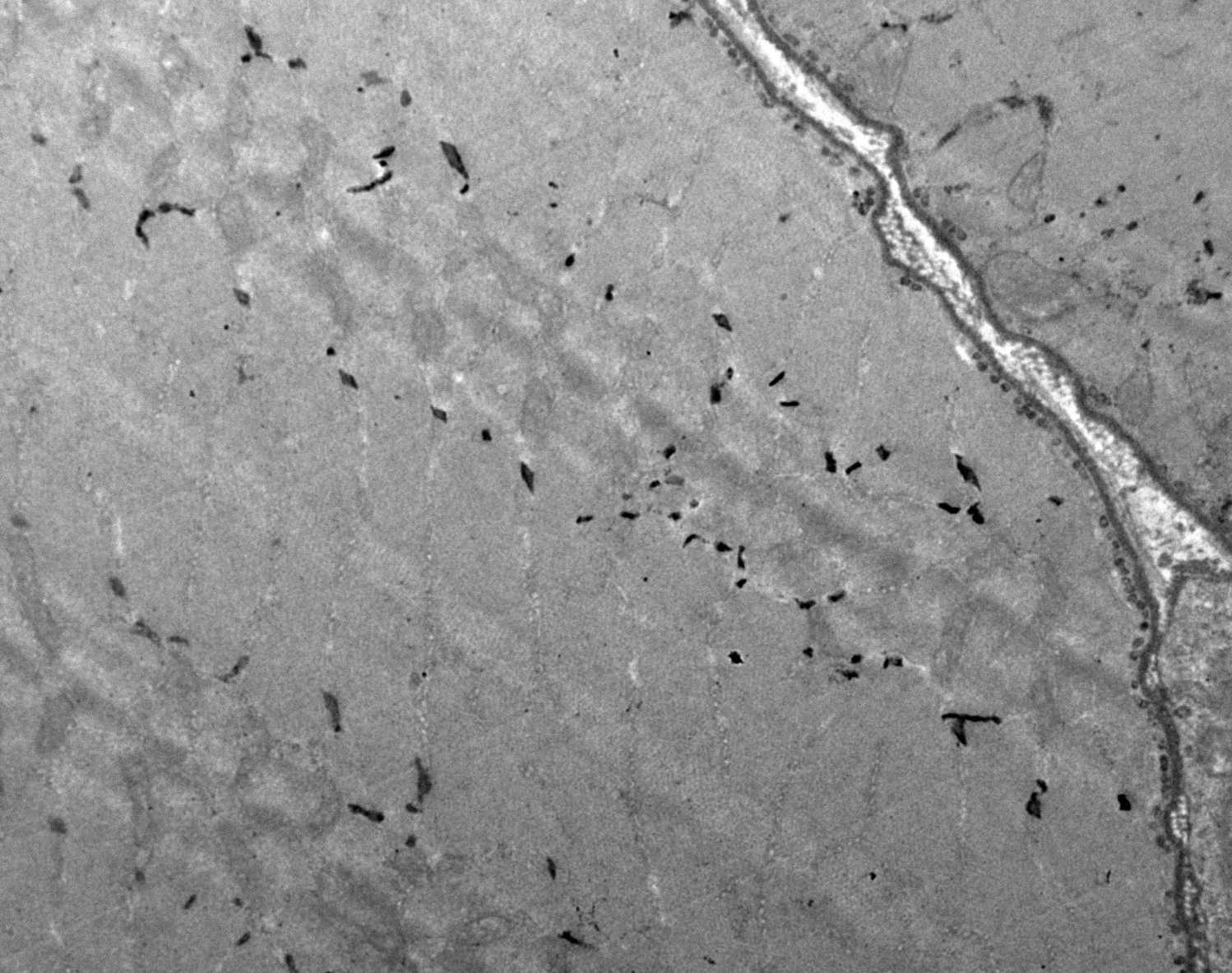

Supplement: Supplementary file 4 — Source data Fig. 2 [file 44318_2024_273_MOESM4_ESM.zip › Figure 2/2C-D/Representative EM image for 8M WT FDB.tif]

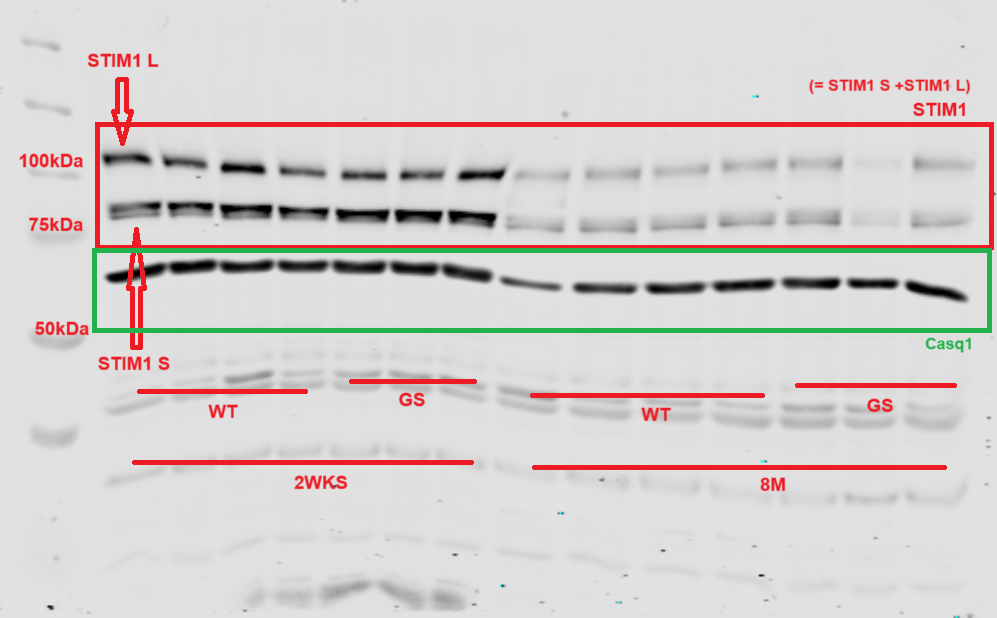

Supplement: Supplementary file 7 — Source data Fig. 5 [file 44318_2024_273_MOESM7_ESM.zip › Figure 5/5A and B/Western Blot for 2wks and 8M Casq1 and STIM1.tif]

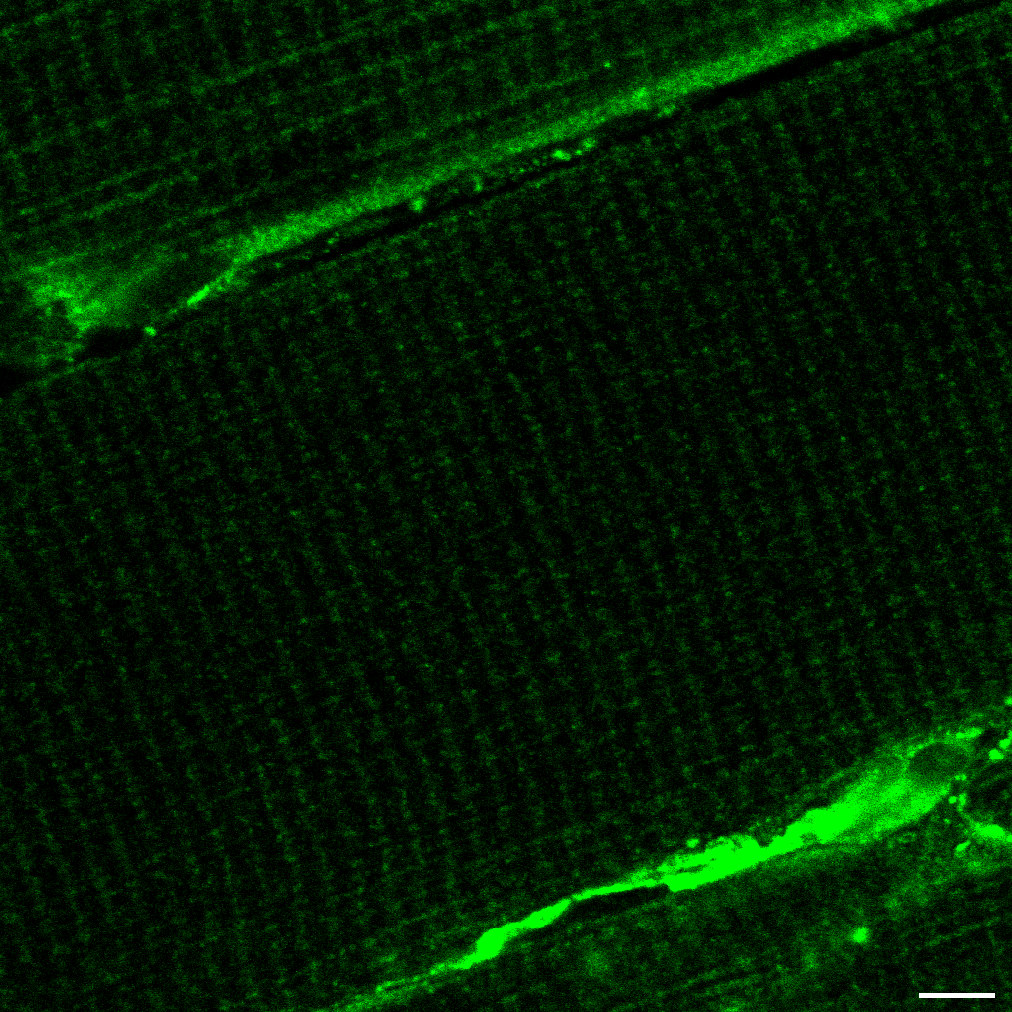

Supplement: Supplementary file 7 — Source data Fig. 5 [file 44318_2024_273_MOESM7_ESM.zip › Figure 5/5E/ICC staining using EDL bundles_ HA antibody.tif]

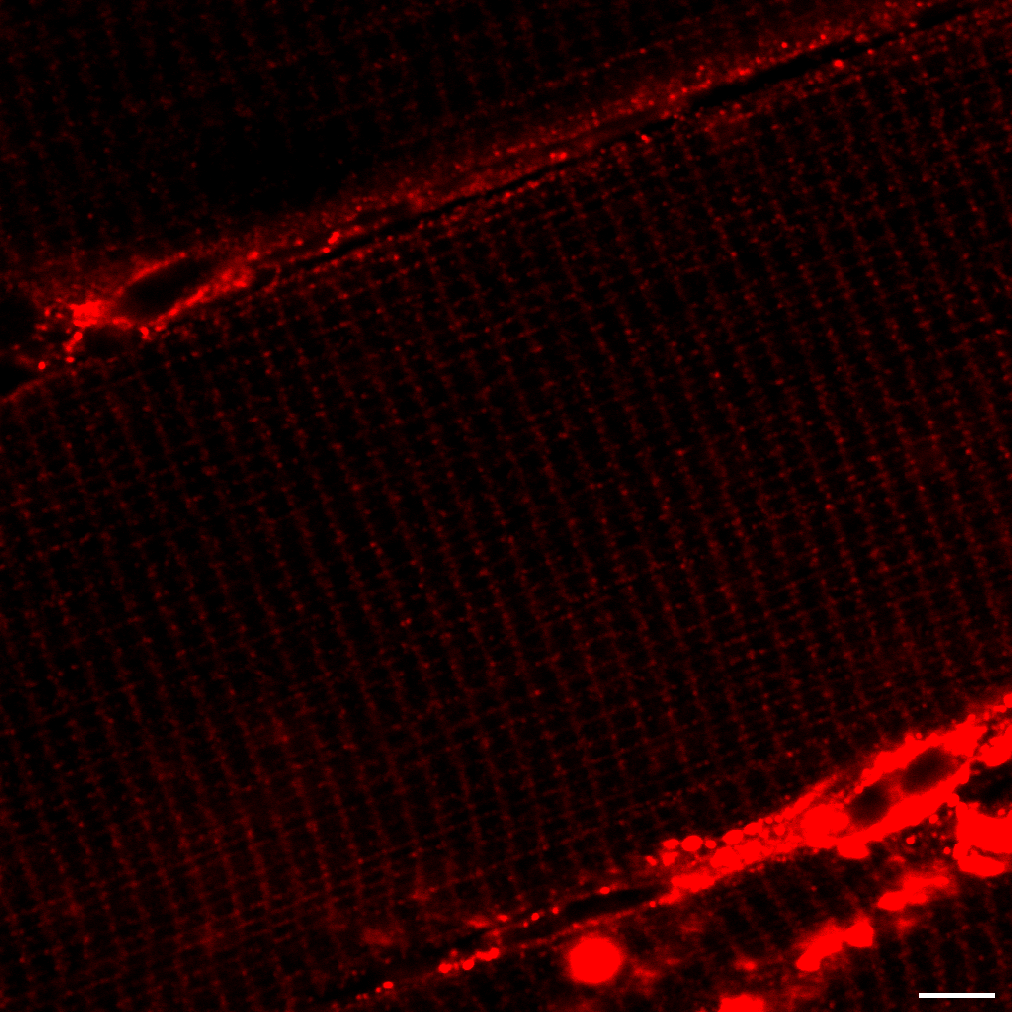

Supplement: Supplementary file 7 — Source data Fig. 5 [file 44318_2024_273_MOESM7_ESM.zip › Figure 5/5E/ICC staining using EDL bundles_ ORAI1 antibody.tif]

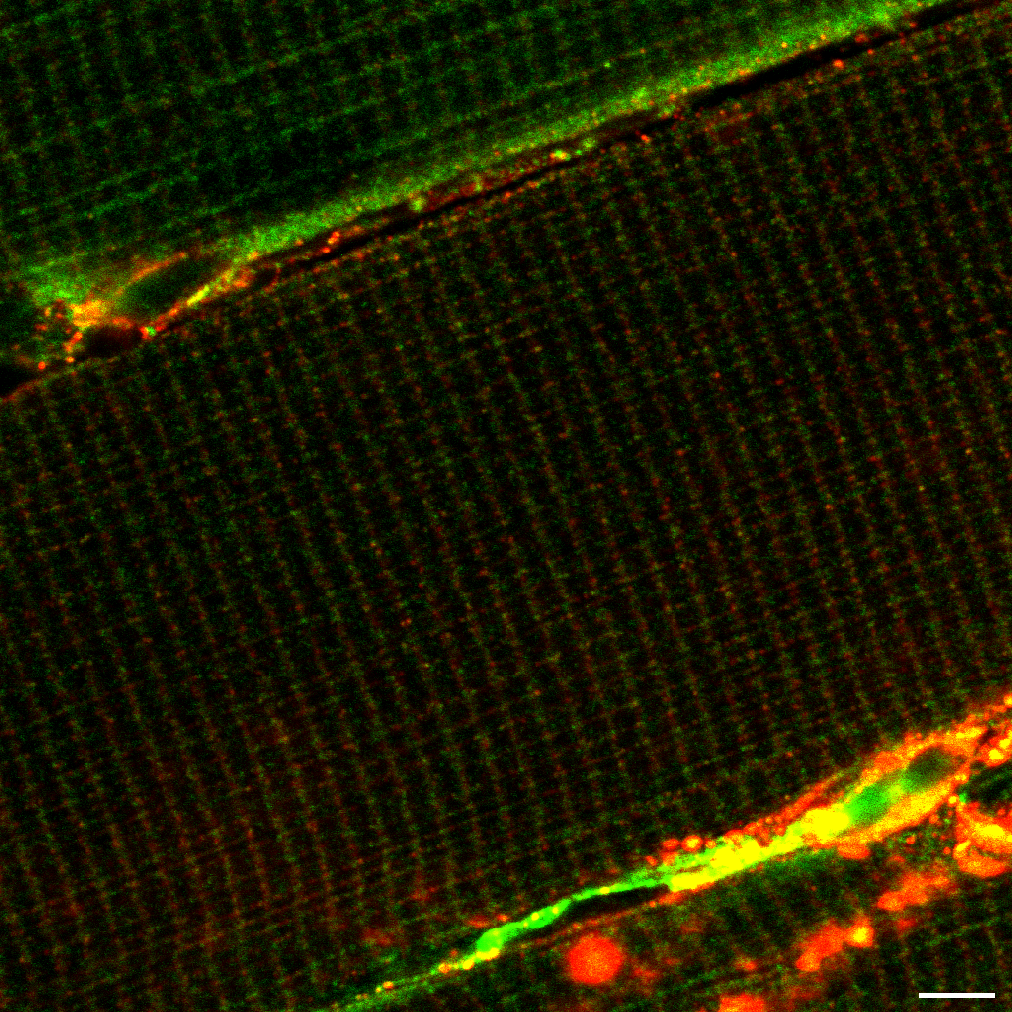

Supplement: Supplementary file 7 — Source data Fig. 5 [file 44318_2024_273_MOESM7_ESM.zip › Figure 5/5E/ICC staining using EDL bundles_merged image.tif]

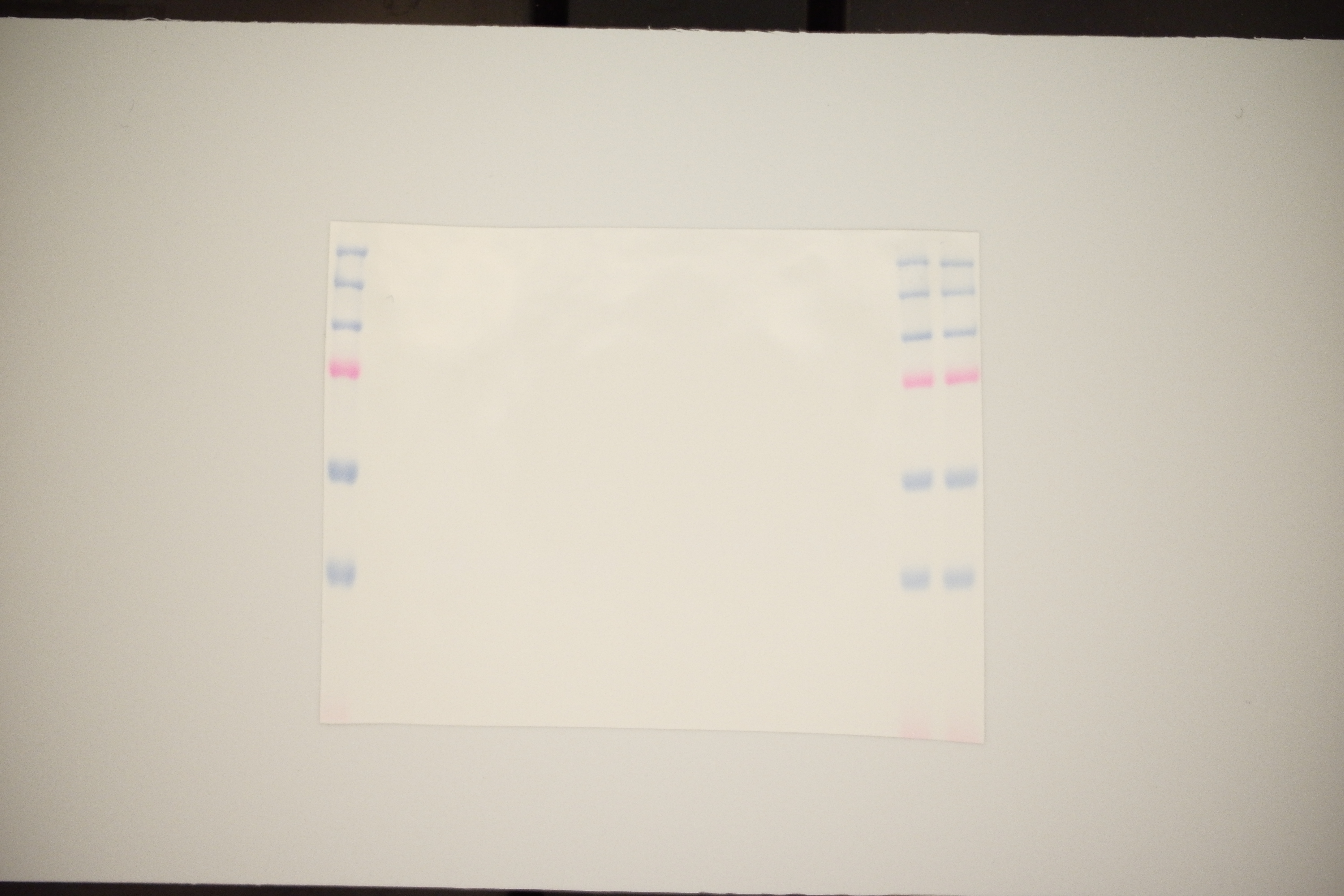

Supplement: Supplementary file 7 — Source data Fig. 5 [file 44318_2024_273_MOESM7_ESM.zip › Figure 5/5F/Western blot marker.tif]

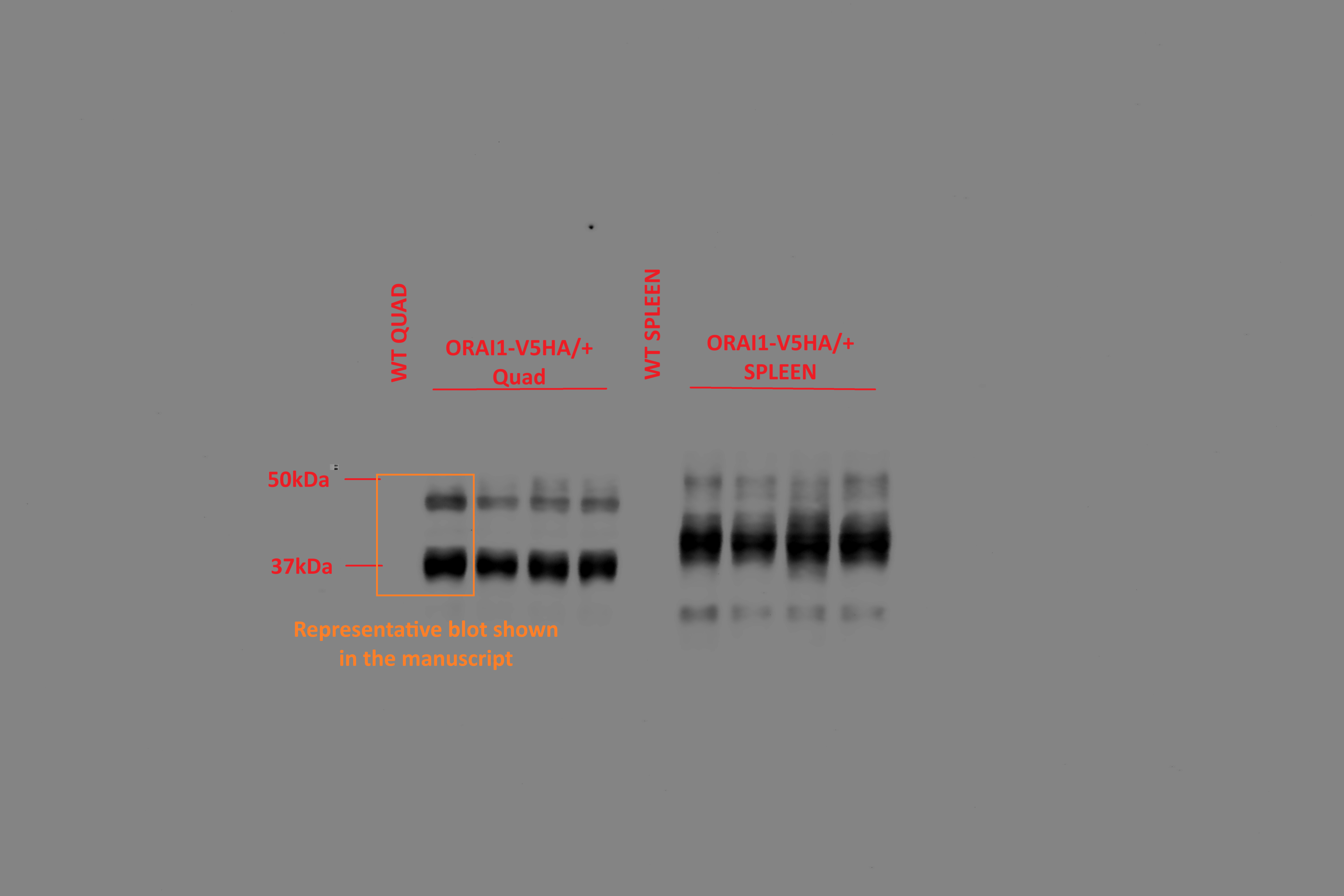

Supplement: Supplementary file 7 — Source data Fig. 5 [file 44318_2024_273_MOESM7_ESM.zip › Figure 5/5F/Western blot using HA antibodies.tif]

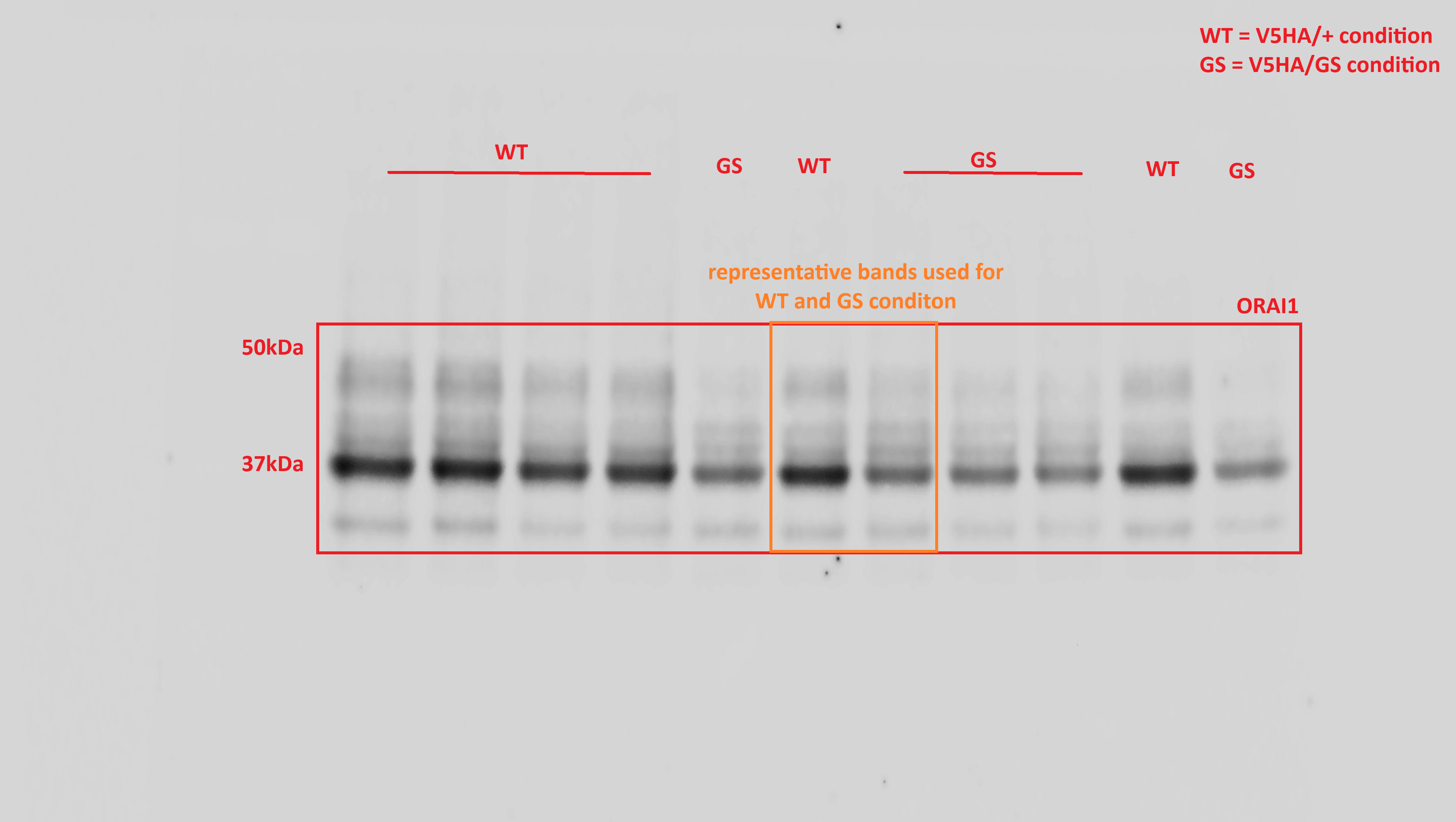

Supplement: Supplementary file 7 — Source data Fig. 5 [file 44318_2024_273_MOESM7_ESM.zip › Figure 5/5G/Western blot for Orai1-HA expression_2wks.tif]

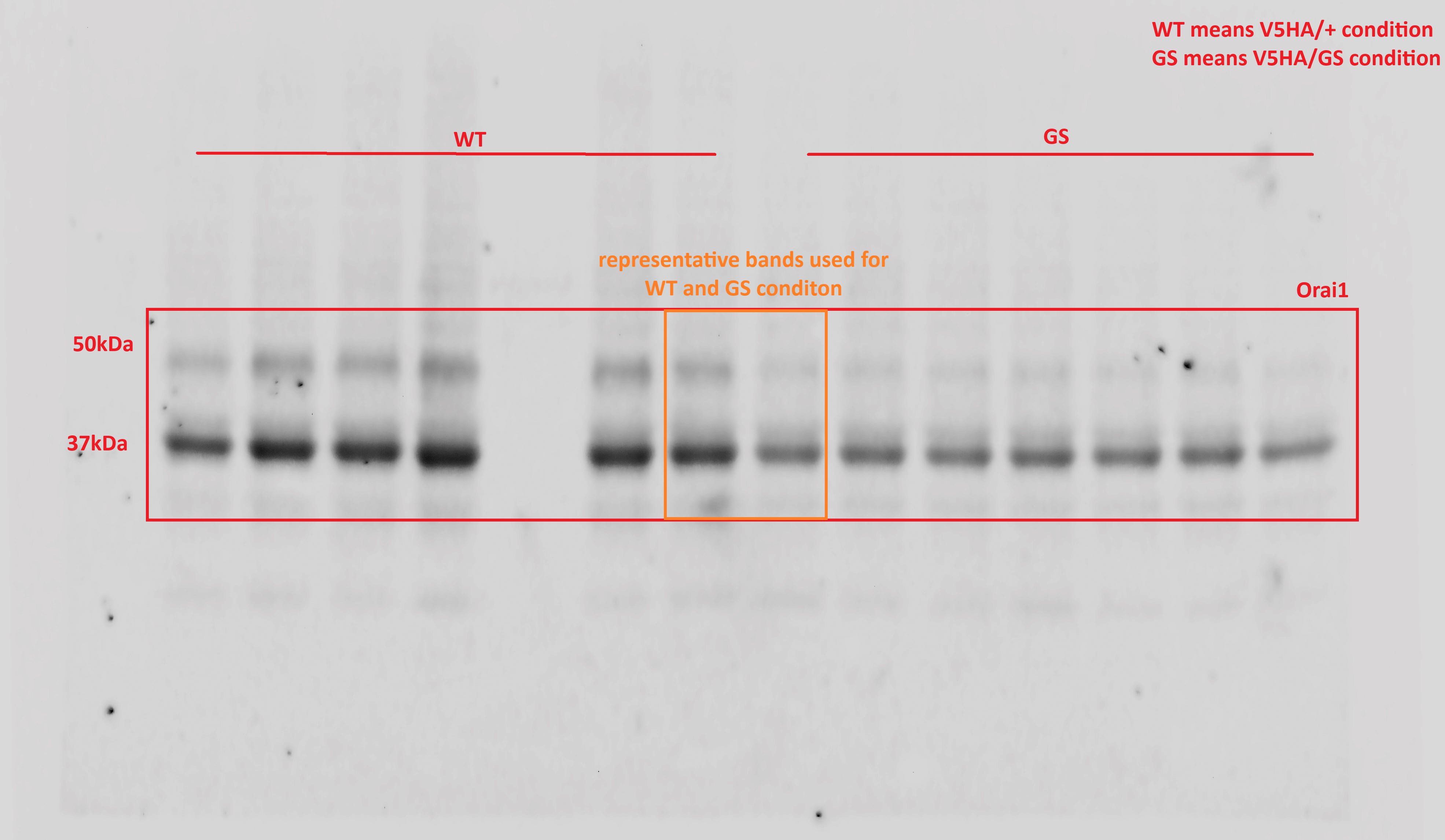

Supplement: Supplementary file 7 — Source data Fig. 5 [file 44318_2024_273_MOESM7_ESM.zip › Figure 5/5H/Western blot for Orai1-HA expression_8M.tif]

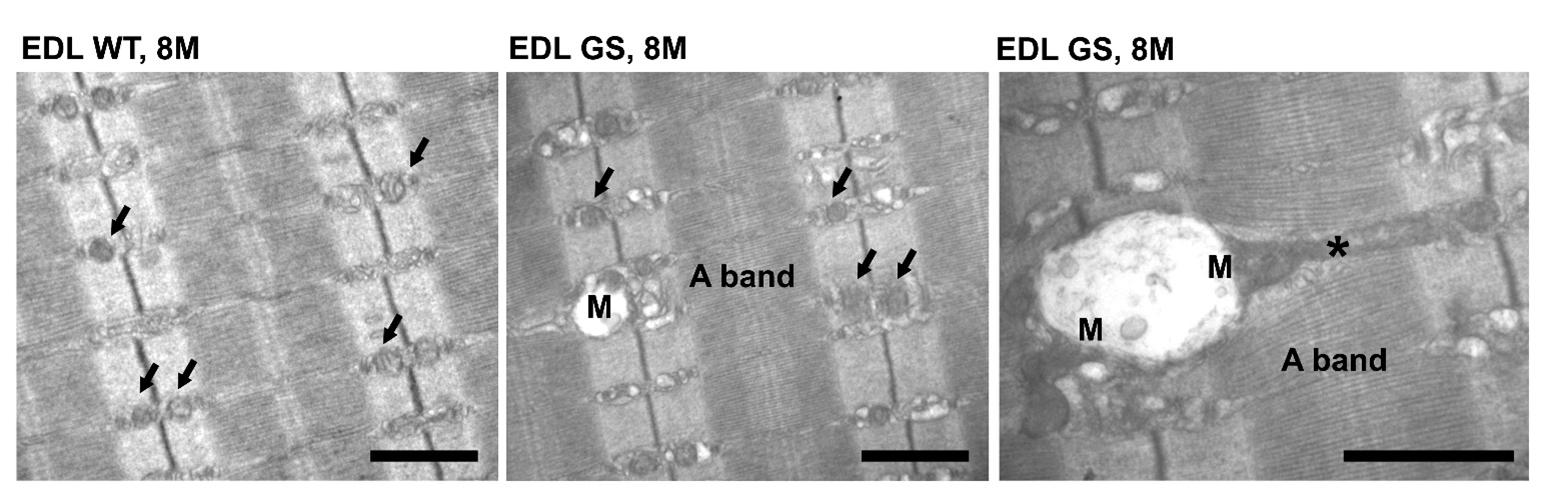

Supplement: Supplementary file 8 — Source data Fig. 7 [file 44318_2024_273_MOESM8_ESM.zip › Figure 7/7A/EM images of WT and GS EDL muscles looking at mitochondria morphology.tif]

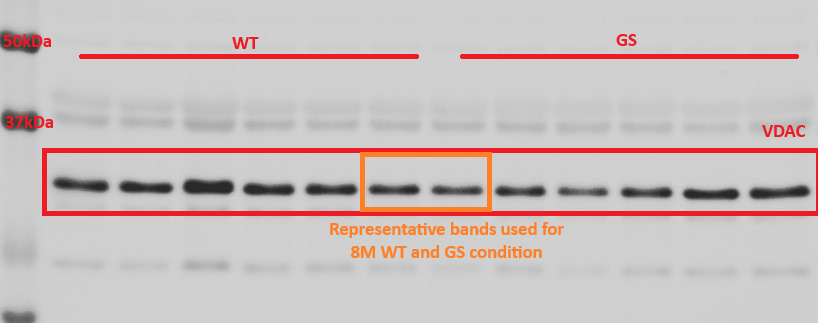

Supplement: Supplementary file 8 — Source data Fig. 7 [file 44318_2024_273_MOESM8_ESM.zip › Figure 7/7C/Western blot for VDAC_8M.tif]
