# Supplementary material for: An Orai1 gain-of-function tubular aggregate myopathy mouse model phenocopies key features of the human disease
Source: EMBO J. 2024 Oct 17;43(23):5941–71. doi: 10.1038/s44318-024-00273-4 (PMC11612304; doi:10.1038/s44318-024-00273-4)
Supplement: Supplementary file 9 — Expanded View Figures [file 44318_2024_273_MOESM9_ESM.pdf]

Expanded View Figures

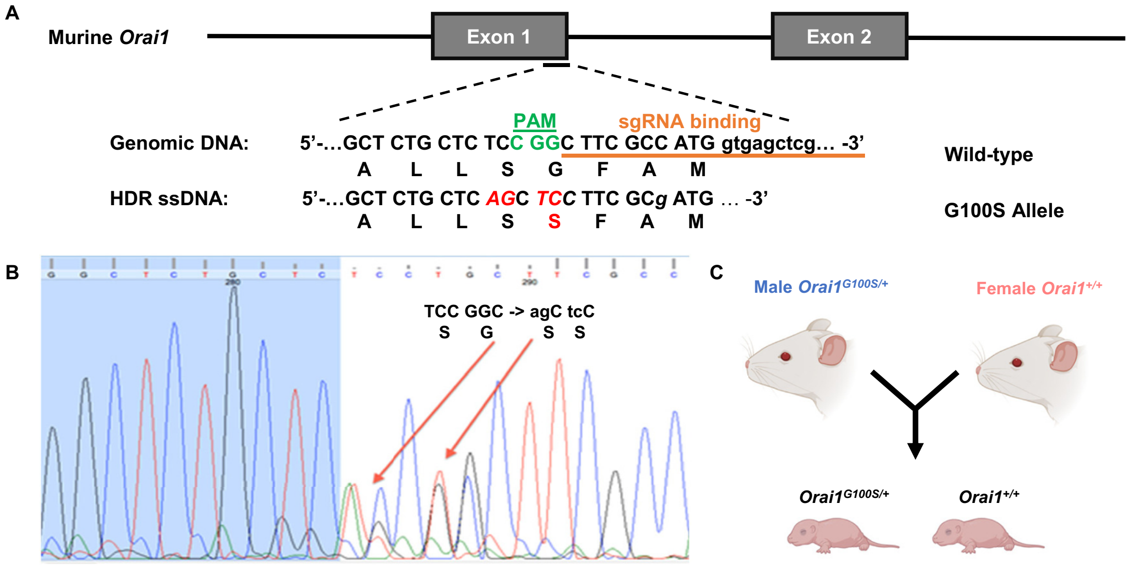

Figure EV1. Generation of GS mouse model.

(**A**) CRISPR-Cas9 strategy used to generate heterozygous GS knock-in mice. The G100S mutation is located at the end of exon 1. A donor template with desired glycine-to-serine mutation and altered PAM region was introduced to mouse embryos. (**B**) Sanger sequencing confirming heterozygous expression of the glycine to serine mutation at ORAI1 amino acid position 100. (**C**) Cartoon depicting the breeding strategy used in this study to generate WT and GS mice.

 © The Author(s)

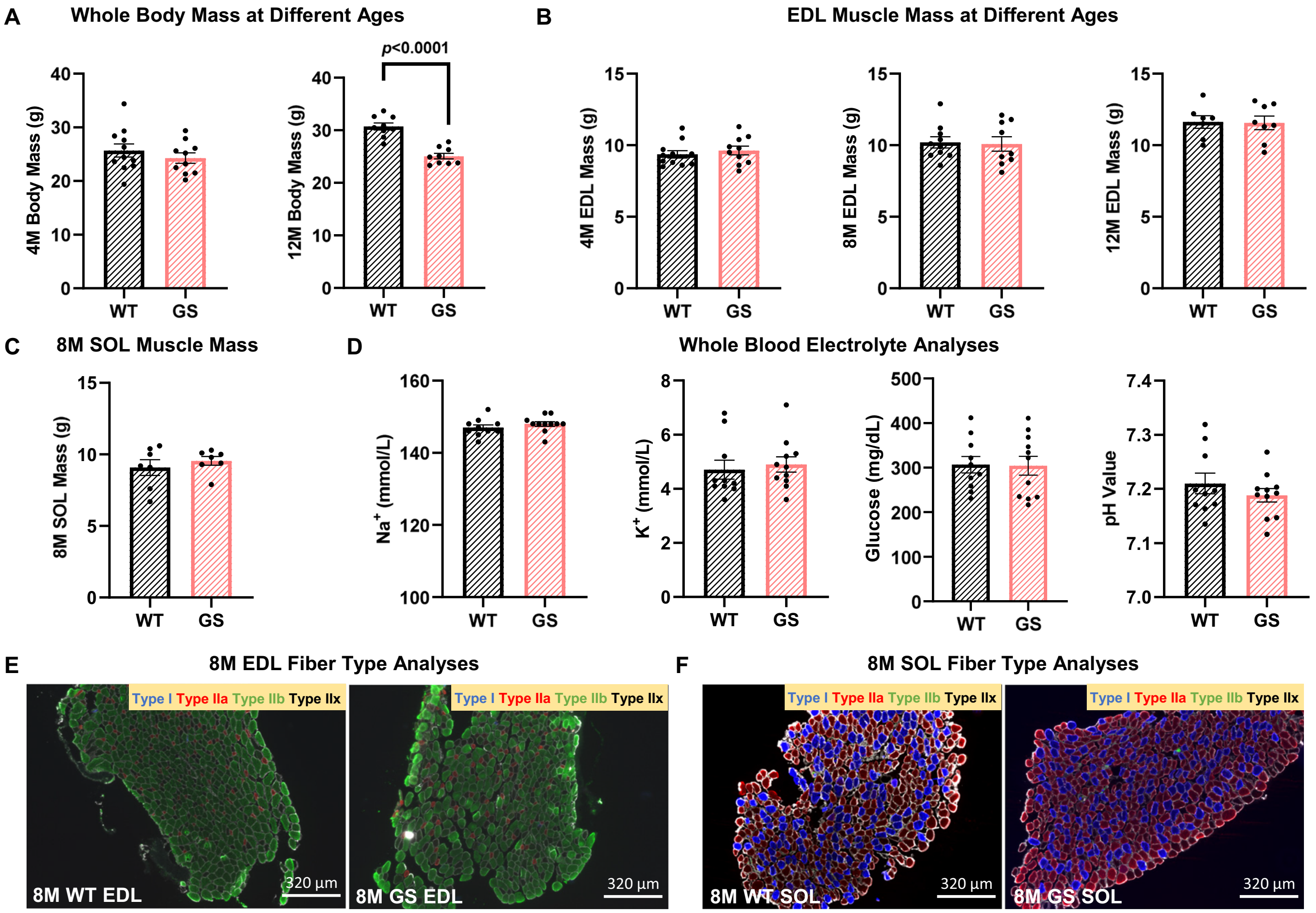

Figure EV2. Additional phenotypic characterization of GS mice.

(**A**) Compared to age-matched WT mice, GS mice showed no significant differences in average body mass at 4 M of age, but significantly reduced body mass at 12 M of age. For 4 M old mice, $n = 11$ and 10 for WT and GS mice, respectively. For 12 M old mice, $n = 9$ for WT mice and $n = 9$ for GS mice. Significance was calculated using Student's t test. The exact P value is labeled in the figure when $P < 0.05$. (**B**) GS mice showed similar EDL muscle mass at 4, 8 and 12 M of age as WT mice. For 4 M old mice, $n = 11$ and 10 for WT and GS mice, respectively. For 8 M old mice, $n = 10$ and 9 for WT and GS mice, respectively. For 12 M old mice, $n = 7$ and 8 for WT and GS mice, respectively. (**C**) WT ($n = 7$) and GS ($n = 7$) mice exhibited similar SOL muscle mass at 8 M of age. (**D**) Whole blood analysis of Na^+, K^+, glucose, and pH from 8 M old WT ($n = 10$) and GS ($n = 11$) mice. (**E**, **F**) Representative fiber type images of EDL (**E**) and SOL (**F**) muscles from 8 M old WT and GS mice.

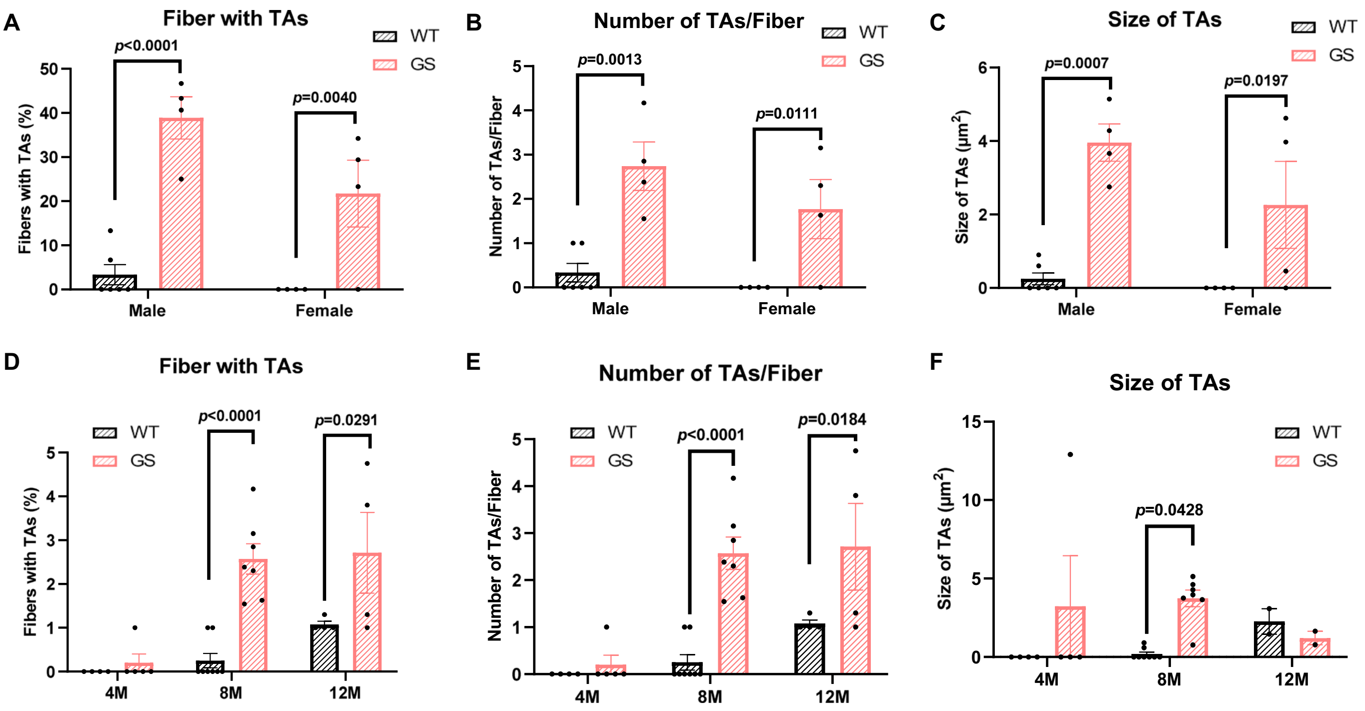

Figure EV3. Sex- and age-dependent analyses of tubular aggregates in EDL muscle from 8 M WT and GS mice.

(**A–C**) Average (± SEM) percentage of fibers with TAs (**A**), number of TAs/fiber (**B**), and TA size (**C**) in EDL muscles from 8 M old male and female WT and GS mice. For male group, $n = 6$ and 4 for WT and GS group, respectively. For female mice, $n = 4$ for WT mice and $n = 4$ GS mice. Significance was calculated using 2-way ANOVA followed by Holm–Sidak's multiple comparisons post hoc test. The exact P value is labeled in the figure when adjusted $P < 0.05$. (**D-F**) Average (± SEM) percentage of fibers with TAs (**D**), number of TAs/fiber (**E**), and TA size (**F**) in EDL muscles from 4 M, 8 M, and 12 M old WT and GS mice. For 4 M and 12 M mice, $n = 4$ for WT mice and $n = 4$ GS mice. For 8 M old mice, $n = 8$ and 7 for WT and GS mice, respectively. Significance was calculated using 2-way ANOVA followed by Holm–Sidak's multiple comparisons post hoc test. The exact P value is labeled in the figure when adjusted $P < 0.05$.

 © The Author(s)

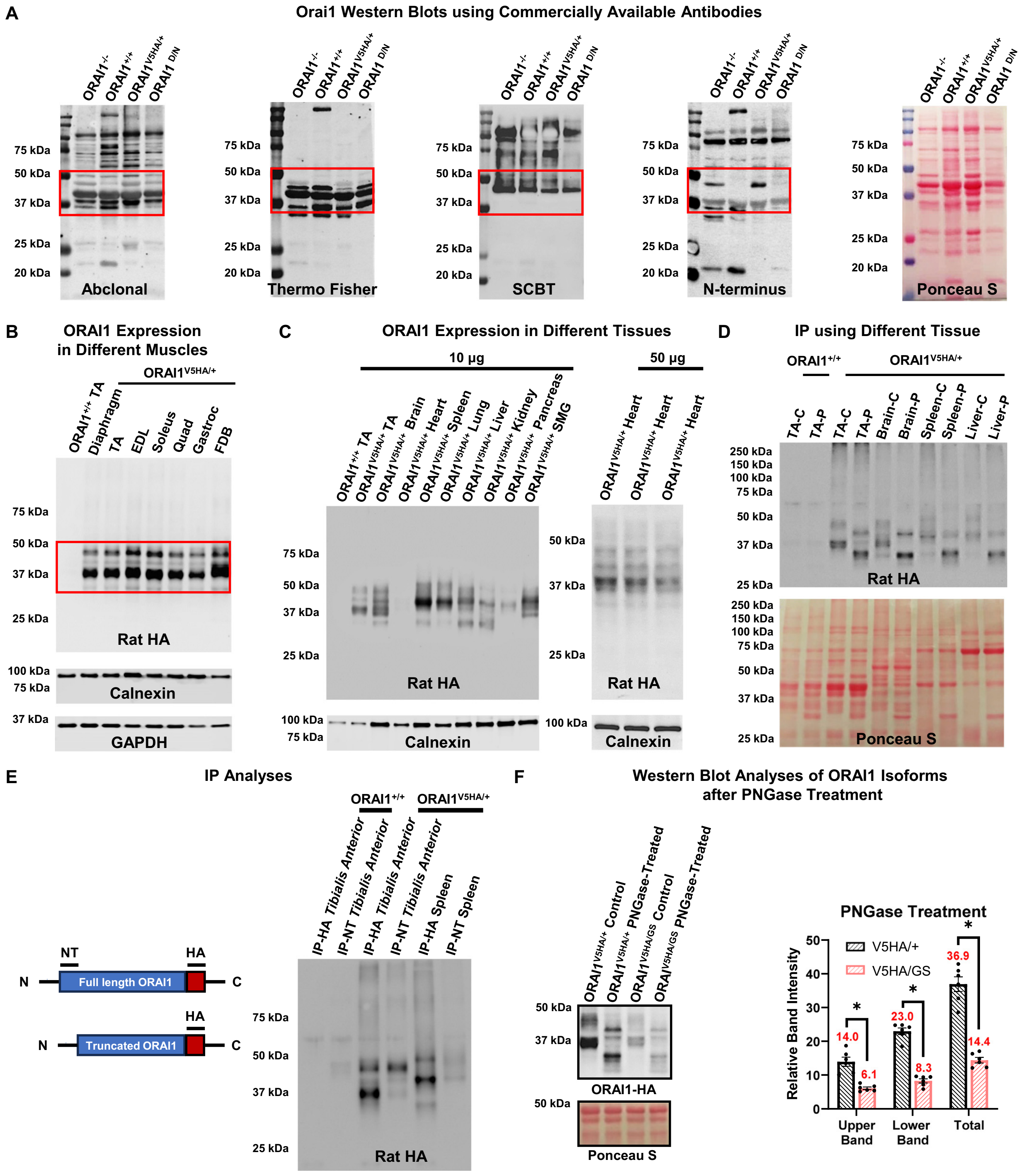
A
Orai1 Western Blots using Commercially Available Antibodies
ORAI1-/-
ORAI1+/+
ORAI1V5HA/+
ORAI1 D/N
75 kDa
50 kDa
37 kDa
25 kDa
20 kDa
Abclonal
Thermo Fisher
SCBT
N-terminus
Ponceau S
B
ORAI1 Expression in Different Muscles
ORAI1V5HA/+
ORAI1+/+ TA
Diaphragm
TA
EDL
Soleus
Quad
Gastroc
FDB
Rat HA
100 kDa
Calnexin
GAPDH
C
ORAI1 Expression in Different Tissues
10 µg
50 µg
ORAI1+/+ TA
ORAI1V5HA/+ TA
ORAI1V5HA/+ Brain
ORAI1V5HA/+ Heart
ORAI1V5HA/+ Spleen
ORAI1V5HA/+ Lung
ORAI1V5HA/+ Liver
ORAI1V5HA/+ Kidney
ORAI1V5HA/+ Pancreas
ORAI1V5HA/+ SMG
ORAI1V5HA/+ Heart
Rat HA
Calnexin
D
IP using Different Tissue
ORAI1+/+
ORAI1V5HA/+
TA-C
TA-P
Brain-C
Brain-P
Spleen-C
Spleen-P
Liver-C
Liver-P
250 kDa
150 kDa
100 kDa
Rat HA
Ponceau S
E
IP Analyses
NT
HA
N
C
Full length ORAI1
Truncated ORAI1
ORAI1+/+
ORAI1V5HA/+
IP-HA Tibialis Anterior
IP-NT Tibialis Anterior
IP-HA Spleen
IP-NT Spleen
Rat HA
F
Western Blot Analyses of ORAI1 Isoforms after PNGase Treatment
ORAI1V5HA/+ Control
ORAI1V5HA/+ PNGase-Treated
ORAI1V5HA/GS Control
ORAI1V5HA/GS PNGase-Treated
ORAI1-HA
Ponceau S
PNGase Treatment
V5HA/+
V5HA/GS
Relative Band Intensity
14.0
6.1
23.0
8.3
36.9
14.4
*
Upper Band
Lower Band
Total

© The Author(s)

Figure EV4. Western blots analyses using commercially available ORAI1 and HA antibodies in various tissues.

(**A**) Representative western blots of muscle lysates from WT mice (ORAI1$^{+/+}$), constitutive muscle-specific ORAI1 KO mice (ORAI1$^{-/-}$), heterozygous V5HA/+ mice (ORAI1$^{V5HA/+}$), and muscle-specific dominant negative ORAI1 transgenic mice (ORAI1$^{D/N}$) probed with commercially available ORAI1 antibodies from Abclonal, ThermoFisher, Santa Cruz Biotechnology (SCBT), and a rabbit polyclonal antibody raised against the first 20 amino acids of the ORAI1 N-terminus. The final gel (right) is the Ponceau S stained gel used for protein loading. No specific ORAI1 band at the expected molecular weight (and absent in the ORAI1 KO lane) was observed for any of these antibodies. (**B**) Representative western blot of endogenous ORAI1 expression probed with rat HA primary antibody across multiple different skeletal muscles (diaphragm, *tibialis anterior* (TA), EDL, SOL, *quadriceps* (Quad), *gastrocnemius* (Gastroc), and FDB) obtained from V5HA/+ mice. The first lane is a *tibialis anterior*(TA) muscle lysate from a WT (*Orai1*$^{+/+}$) mouse used as a negative control. Calnexin and GAPDH loading control blots are shown below the rat HA blot. Two different Orai1-V5HA-tagged isoforms are observed in each muscle. (**C**) Representative western blot of endogenous ORAI1 expression probed with rat HA primary antibody across multiple different organs (*tibialis anterior* (TA), brain, heart, spleen, lung, liver, kidney, pancreas, and submandibular gland (SMG)) obtained from V5HA/+ mice. 10 µg (left) or 50 µg (right) lysate was loaded in each lane. Calnexin loading control blots are shown below the rat HA blots. A *tibialis anterior* muscle lysate from a control WT mouse (*Orai1*$^{+/+}$) was used as a negative control. Note: Low level ORAI1 protein expression in the heart is best observed when loading 50 µg of lysate. Two distinct Orai1-V5HA-tagged isoforms are observed, though at somewhat different levels of glycosylation. (**D**) Representative rat HA western blot (upper) of skeletal muscle (*tibialis anterior* (TA)), brain, spleen, and liver lysates from *Orai1*$^{V5HA/+}$ mice without (C) and after treatment with PNGase (P). A skeletal muscle (*tibialis anterior* (TA)) lysate from a control WT mouse (*ORAI1*$^{+/+}$) was used as a negative control. The lower blot shows protein loading using Ponceau S stain. (**E**) Schematic of full-length and N-terminal truncated V5-3xHA-tagged ORAI1 isoforms (left) and a representative western blot of *tibialis anterior* (TA) immunoprecipitation (IP) using either a rat HA antibody (left lane pairs) or the rabbit polyclonal antibody raised against the first 20 amino acids of the mouse ORAI1 N-terminus (right lane pairs) and then blotted with rat HA primary antibody. While both ORAI1 isoforms are readily pulled-down with the rat HA antibody, the larger full-length ORAI1 isoform is preferentially pulled-down by the N-terminal antibody that recognizes the full-length isoform. These results are consistent with the smaller isoform reflecting an alternative N-terminal translation initiation as reported previously (Desai et al, 2015; Fukushima et al, 2012). (**F**) Representative HA western blot (left) of *tibialis anterior* muscle in the absence (control) and after treatment with PNGase (PNGase-treated) of skeletal muscle (*tibialis anterior*) from either a control V5HA/+ mouse or a V5HA/GS mouse. The lower blot shows total protein loading using Ponceau S stain. Average (± SEM) bar plot of relative HA-reactive upper, lower, and total (upper+lower) band intensity after PNGase treatment for muscle lysates from V5HA/+ (*black*, $n = 6$) and V5HA/GS mice (*pink*, $n = 6$) (right). Protein expression levels were normalized to Ponceau S. Mean relative band intensity for each condition is noted in red above each bar. Significance was calculated using 2-way ANOVA followed by Holm–Sidak's multiple comparisons post hoc test. * indicates adjusted $P < 0.05$ with all $P < 0.0001$.

 © The Author(s)

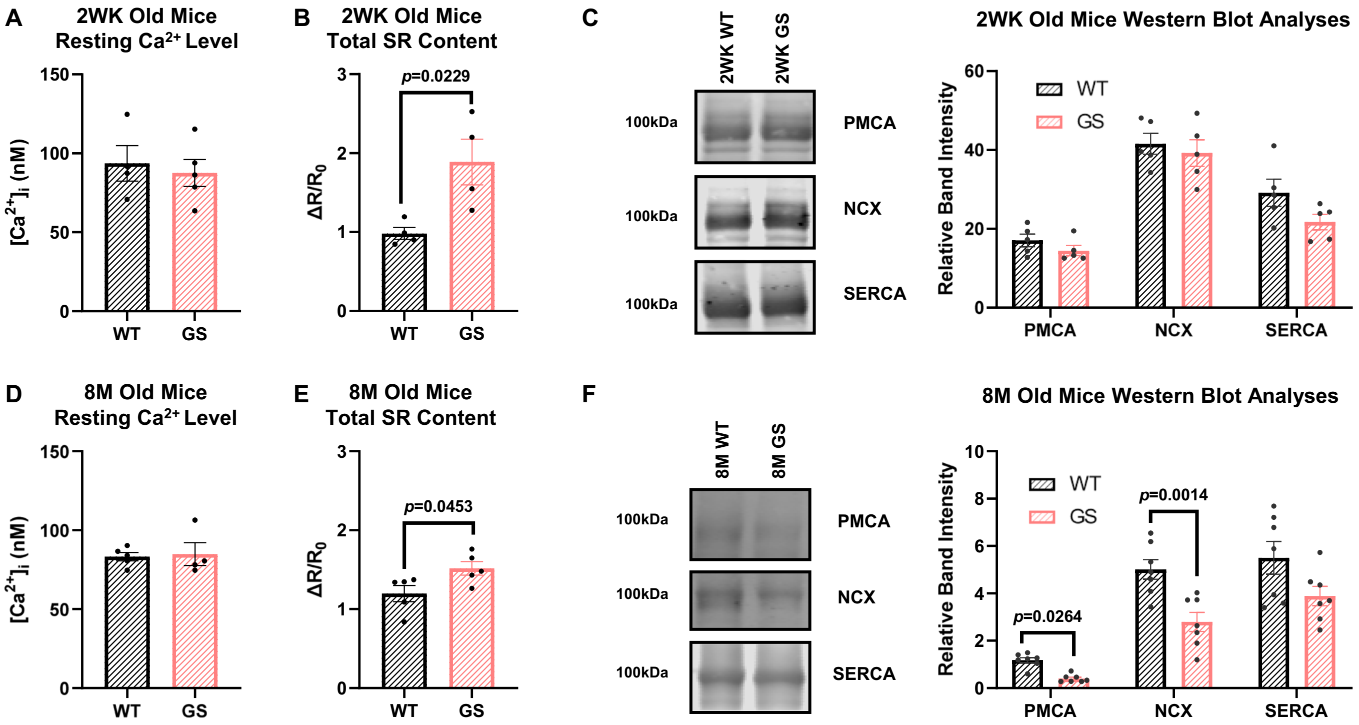

Figure EV5. GS mice show altered Ca^{2+} handling ability at both 2 WK and 8 M of age.

(**A**, **D**) No significant alterations in resting Ca^{2+} level were observed in FDB fibers from GS mice at both 2 WK and 8 M of age. For 2 WK old mice, $n = 4$ and 5 for WT and GS mice, respectively. For 8 M old mice, $n = 5$ and 4 for WT and GS mice, respectively. (**B**, **E**) SR Ca^{2+} storage was significantly increased in FDB fibers from GS mice at both 2 WK and 8 M of age. For 2 WK old mice, $n = 4$ WT mice and $n = 4$ GS mice. For 8 M old mice, $n = 5$ WT mice and $n = 5$ GS mice. Significance was calculated using Student's t test. The exact P value is labeled in the figure when $P < 0.05$. (**C**) Expression of PMCA, NCX and SERCA were not significantly altered in *tibialis anterior* muscle lysates from 2 WK old GS mice. Protein expression levels were normalized to Ponceau S. $n = 5$ for all experiment groups. (**F**) Expression of PMCA and NCX, but not SERCA, were significantly decreased in *tibialis anterior* muscle lysates from 8 M old GS mice. Protein expression levels were normalized to Ponceau S. $n = 5$ for all experiment groups. Significance was calculated using 2-way ANOVA followed by Holm-Sidak's multiple comparisons post hoc test. The exact P value is labeled in the figure when adjusted $P < 0.05$.

© The Author(s)
