# Supplementary material for: An Orai1 gain-of-function tubular aggregate myopathy mouse model phenocopies key features of the human disease
Source: EMBO J. 2024 Oct 17;43(23):5941–71. doi: 10.1038/s44318-024-00273-4 (PMC11612304; doi:10.1038/s44318-024-00273-4)
Supplement: Supplementary file 2 — Peer Review File [file 44318_2024_273_MOESM2_ESM.pdf]

THE EMBO JOURNAL

An *Orai1* gain-of-function tubular aggregate myopathy mouse model phenocopies key features of the human disease

Nan Zhao, Antonio Michelucci, Laura Pietrangelo, Sundeep Malik, Linda Groom, Jennifer Leigh, Thomas O'Connor, Takahiro Takano, Paul Kingsley, James Palis, Simona Boncompagni, Feliciano Protasi, and Robert Dirksen

Corresponding author(s): Robert Dirksen (Robert_Dirksen@URMC.Rochester.edu)

Review Timeline:

Submission Date:	24th Jan 24
Editorial Decision:	27th Feb 24
Appeal Received:	3rd Jul 24
Editorial Decision:	30th Aug 24
Revision Received:	19th Sep 24
Accepted:	27th Sep 24

Editor: Kelly Anderson

Transaction Report:

(Note: With the exception of the correction of typographical or spelling errors that could be a source of ambiguity, letters and reports are not edited. Depending on transfer agreements, referee reports obtained elsewhere may or may not be included in this compilation. Referee reports are anonymous unless the Referee chooses to sign their reports.)

1st Editorial Decision **27th Feb 2024**

Dear Dr. Dirksen,

Thank you for submitting your manuscript (EMBOJ-2024-116743) to The EMBO Journal. We have now received three reports back and, based on their comments, we unfortunately will not move forward with publication in The EMBO Journal. As you will see below, the referees raise substantial concerns including whether the model is suitably relevant to human myopathy given the divergent phenotypes and whether enough novel biological insight is provided.

However, given the general interest in this area, I discussed your work with my colleague Martina Rembold at EMBO Reports who found the work interesting and would be happy to consider a revised version of your manuscript which includes the following: "To revise your study for EMBO Reports, we ask you to strengthen the following aspects: - add further data on mitochondrial function (OXPHOS, glycolysis, metabolic enzymes) and ideally mitochondrial morphology (ref#1, point 2, ref#2, point 7). Transcriptomics on muscle from GS mice (referee 1, point 2) are not required. - provide a more thorough description and analysis of the tubular aggregates observed in different muscles of OraiG100S/+ mice and TAM patients (if available), along the lines suggested by referee 2. - reconcile the data on Orai1 protein downregulation as adaptive response (ref#1, ref#2, ref#3) and the observed discrepancies regarding SOCE and disease onset (ref#2, point 4). I am happy to discuss the revision further via e-mail or video call. Please contact me at m.rembold@emboreports.org."

We very much hope you will be interested in this option: please follow the link below for transfer.

Yours sincerely,

Kelly M Anderson, PhD
Editor, The EMBO Journal
k.anderson@embojournal.org

Referee #1:

Review of Zhao et al. Zhao et al report a new heterozygous knock-in mouse with a point mutation in Orai1 (G100S) that shows tubular aggregate myopathy (TAM) with compromised skeletal muscle function. The gist of this study is that the authors claim to provide a model to study a form of heritable TAM in humans that is characterized with the presence of tubular aggregates, muscle weakness and exercise intolerance. They report that 8-month-old Orai1 GS+/- present with tubular aggregates, muscle weakness, exercise intolerance and elevated creatine kinase (CK) levels, phenocopying the human myopathy. The equivalent Orai1 point mutation in humans is G98S and is a gain of function (GoF) mutation with enhanced Orai1 activity under basal conditions, in the absence of store depletion. The authors report that constitutive calcium entry in the GS+/- mouse is only seen in skeletal muscle during early development but not in adult muscle and contend that this is at least partially explained by a postnatal adaptive reduction in Orai1 protein expression. Finally, the authors performed proteomic analysis on 2-week-old and 8-month-old wildtype and GS+/- mice and showed that mitochondrial-related pathways are altered in skeletal muscle of the GS+/- mouse.

This is an important manuscript that reports a novel Knock-in mouse with significant relevance to a human myopathy. The authors have also generated an HA-tagged Orai1 knock-in mouse that was crossed to the GS+/- mouse to evaluate the effect of the GS mutation on the expression of wildtype Orai1 protein. The mouse was characterized for muscle force generation as well as calcium measurements of Orai1 activity under basal and store-depletion activation in fast twitch EDL, FBD and SOL muscles. However, the study is somewhat descriptive with a paucity of molecular insights beyond expected findings on muscle weakness and constitutive calcium activity. Interesting observations are reported that include the adaptive postnatal downregulation of wildtype Orai1 and the mitochondrial phenotype, but neither of these observations are studied in-depth to shed light on their connection to the GS mutation. This and some missing data and controls are described below:

1) The authors acknowledge that crossing GS and HA- mice will determine only changes in expression of wildtype Orai1 proteins, which is a weakness. How the GS mutation is affecting the expression of wildtype Orai1 and whether this also applies to the GS protein is not clear. Orai1 mRNA is not affected, and the authors speculated about potential effects on protein trafficking and glycosylation, but none of this is substantiated by evidence. First, we know from previous studies that human Orai1 G98S locates normally to the plasma membrane and mediates activity. The presence of constitutive calcium flux in 2 weeks old (Fig 4B) is not consistent with defects in trafficking or glyosylation. The suggestion that the Orai1 higher band is decreased much more significantly than the shorter band is not supported by the data presented. Both looks decreased to the same extent and the final densitometry numbers provided appear to this reviewer to be likely an artifact in densitometry measurements, likely because the short Orai1 band in the wildtype is saturated (Fig 5G, H). Glycosylation is an unlikely explanation as all bands are there in both WT and GS, just less dense in GS. One recommendation is to declycosylate the proteins samples; in this case, one should expect to see two distinct bands at 25KD and 33KD and one would expect to reliably determine whether both short and long Orai1 are decreased equally or differentially in the muscle of GS mice.

2) The authors provide no data on mitochondrial structure, shape and morphology using TEM, including a thorough comparison of the two types of mitochondrial populations between WT and GS mice at 2 weeks and 8 months of age. They also provide no direct measurements of mitochondrial function or metabolism in general. The proteomics data provided and the alterations in mitochondrial-related pathways is quite vague and superficial. Previous studies on several cell types (T cells, B cells, astrocytes and Smooth muscle), showed that mice lacking either STIM or Orai have defective OXPHOS and glycolysis with altered expression of several metabolic enzymes and transporters (e.g. GLUT) regulating these pathways. Presumably, the decrease in Orai1 reported in the GS mouse could explain these mitochondrial alterations, but from this manuscript 8 mitochondrial-related proteins involved in lipid oxidation and OXPHOS are actually upregulated (not downregulated) and its not clear how these data can be rationalized within the current literature on Orai1 channels. Have the authors considered performing transcriptomics on muscle from GS mice to contrast with previous published findings on other cell types mentioned above?

3) It is not clear why the authors do not provide direct measurements of SR calcium as was done for mitochondria.
Minor:
4) It was quite difficult to evaluate this manuscript because the literature cited in the manuscript is referenced solely with the year of publication, making difficult to identify the exact paper referenced in the list of references.
5) For FTEM in Figure 2E, It is missing the wildtype controls
6) Fig 3C, the Y axis is mislabeled as "cumulative rest"
7) Lane 738, lists one wrong reference (2010); it should be a 2017 reference (PMID28219928).

Referee #2:

In the present study, Zhao and colleagues describe a novel Orai1 mouse model for tubular aggregate myopathy. The article presents some novel data, but the study is largely descriptive and lacks relevant functional studies. To my opinion, the manuscript is not suitable for the EMBO Journal in its current state. Here are my comments:

Major points:
1. Although Orai1 G100S mice manifest muscle weakness, they only partially recapitulate the human disorder. The authors point out that patients with the Orai1 G100S mutation show fiber size variability, type I fiber predominance, internal nuclei and fibrosis on muscle biopsies, but none of these histological features is seen in G100S mice. Additional Orai1-typical signs like miosis, hypocalcemia, or - described in one patient with G100S mutation - bleeding diathesis are also absent, questioning the relevance of the mouse model for further physiological investigations or the development of therapies.

2. In contrast to other existing TAM mouse models, Orai1 G100S mice exhibit tubular aggregates in muscle fibres, which is certainly an interesting point. However, aggregates were not found in the soleus although muscle force was reduced. What is then the relevance of the tubular aggregates and their contribution to the disease? The authors speculate that the increased resistance to fatigue of the soleus muscle may explain the absence of aggregates, but what is the link between both? And why is there no difference in force decline between EDL and soleus after tetanic stimulation? A major difference between EDL and soleus is the fibre type composition. EDL is a fast muscle and soleus a slow muscle. Are aggregates only found in type II fibres? How about mixed muscles like the gastrocnemius? To my opinion, it would be interesting to investigate the tubular aggregates in detail. What is their composition? Is it the same as the tubular aggregates in TAM patients and the aggregates occurring during the ageing process in WT mice?

3. It is intriguing that G100S mice are phenotypically normal at 4 months and develop a muscle phenotype at 8 months, while calcium entry is increased in muscle fibers from young mice and normal in older mice. The authors hypothesize that Orai1 expression diminishes over time to compensate calcium entry - but why does the disease then start after this compensatory and protective effect?

4. Figure 4: constitutive calcium entry is increased in FDB muscle fibres from embryonic development to 5 weeks of age, but SOCE is strongly decreased in the same period. Do the authors have an explanation for this contradictory result? The increased calcium entry indicates that the Orai1 channel is leaky, and the activation of SOCE through TG is expected to amplify (and not to attenuate) calcium entry and Mn^{2+} quenching.

5. The methods used to determine the Orai1 protein levels are very indirect and the results are not convincing. Figure S4A shows two Orai1 bands in different muscles - probably the alpha and beta isoforms. But figure S4B shows a different picture with a single intermediate band in spleen, lung, liver and kidney. What is this if not Orai1 alpha and Orai1 beta? And why is there no signal in heart? According to the gtex database, Orai1 expression is similar in heart, kidney in liver. Has this experiment been repeated? In figure S4C, the Orai bands without PNGase treatment are again different and e.g. higher in spleen and liver than in brain and muscle. In addition, IF (although only semi-quantitative) in figure S6 does not seem to indicate a reduction of Orai1

protein levels. It may be better to test all commercially available Orai1 antibodies (or to make one) instead of evaluating Orai1 expression through V5HA mice.

6. In the same line, the IP results are not convincing. The authors state that only full-length Orai1 is pulled down with the N-terminal Orai1 antibody. This is not true. The western blot on figure S4D clearly shows both Orai1 bands in V5HA mice. And why is full-length Orai1 reduced 4-fold ("almost abolished") in V5HA/G100S mice although the V5HA is expected to produce 50% normal protein? Taken all WB and IP results together, it seems difficult to draw any conclusions on Orai1 protein levels.

7. The proteomics data are interesting and represent a novelty. They indicate an implication of mitochondrial pathways, but no functional data are presented. To emphasize the physiological relevance of the proteomics data, it is necessary to perform additional quantitative experiments on mitochondrial respiration, and potentially also on mitochondrial fusion and fission. And what did proteomics reveal about Orai1 protein levels? Orai1 does not seem to appear in the downregulated proteins.

Minor comments:
1. Lines 448-449: the sentence "The maximum rate of EDL force production during tetanic stimulation was also reduced significantly only in EDL muscle" is not quite clear to me.

2. The western blot in figure 5A shows two main Stim1 bands at two weeks and three main bands at 8 months. Which is the right one? Which one has been used to quantify the protein level?

3. Lines 537-539: The authors state that the smaller Orai1 band on figure 5G represents a combination of the glycosylated longer Orai1 isoform and the unglycosylated shorter Orai1 isoform. However, the PNGase experiments in figure S4C clearly show that unglycosylated full-length Orai1 has a higher molecular weight than the glycosylated short Orai1 isoform.

4. The authors speculate that impaired Orai1 glycosylation contributes to the reduction of SOCE in Orai1 G100S mice. Actually, it is the inverse. Impaired Orai1 glycosylation results in channel overactivity (Dorr et al., Science Signaling 2016).

5. Figure S3: It is a bit surprising to see that 4 Orai1 G100S mice have the exact same number of monocytes in blood.

Referee #3:

The paper by Zhao et al. describes a novel mouse model that expresses Orai1 G100S, which is analogous to the GOF mutant ORAI1 G98S identified in patients presenting with Tubular Aggregate Myopathy (TAM). Similar to the Stim1 GOF models, they document muscle weakness and exercise intolerance in the GS-mouse. They also detect tubular aggregates (TAs) in fast twitch EDL and FDB muscle, and document constitutive calcium entry in FDB muscle fibers in 2-5wk old GS mice, which was absent in FDB fibers isolated from GS mice older than 4 months. The authors argue that the reduced Orai1 protein levels detected in tibialis anterior muscle in the GS mice may compensate for the Orai1 G100S-mediated constitutive calcium influx.

The authors present a substantial collection of results, which warrants publication, but significant revisions of the presentation are required to increase the quality of the manuscript allowing a thorough review of the manuscript.

First of all, the authors need to correct the major deficiencies in citations and the reference list, which significantly reduce the quality of the paper and impede a thorough review of the manuscript: The citations in the text consist of only the publication years and the reference list lacks journal names. These deficiencies cause challenges for the review as exemplified by a statement on page 4 «Three different STIM1 mouse models ... were previously described (2020; 2018; 2007; 2019)». There are four different Stim1 GOF mouse models described in the four years mentioned (Grosse et al. 2007, Gamage et al. 2018, Silva-Rojas et al. 2019, and Cordero-Sanchez et al. 2020). The paper by Grosse et al. 2007 lacks in the reference list. On page 28, the authors mention an additional mouse model, Stim-L96V, but such a paper lacks in the reference list. The authors describe the clinical features in patients with Stormorken syndrome, but they do not cite the original paper by Stormorken et al. (1985), and the paper from his group identifying the causal mutation in 2014.

The authors used a HA antibody to explore if impaired maturation or mis-localization of Orai1 caused the reduction in constitutive calcium entry and reduced Orai1 protein level in muscles in GS mice older than 4 months. However, these experiments detect only the wild type protein in Orai1-V5HA/GS mice expressing both Orai1 WT with a C-terminus HA epitope and Orai1 G100S. There are Orai1 antibodies used by other researchers that could be used to quantify the Orai1 G100S level and assess the intracellular localization.

Additional comments:
1) On page 16 the authors state «The number and size of TA inclusions were greater in muscles from 18M old GS mice (Fig. 2B and D)». The statement indicates a significant difference in the number and size of TA inclusions in muscles at 18M and 12M, which is not indicated in Fig. 2. Further, they claim that «TA structures in fast twitch EDL and FDB muscles (but not SOL muscles) of 8M old GS mice (Fig. 2E)». The results in SOL muscles are not included in Fig. 2.
2) On page 19, the authors state that «fibers were pretreated for 1 hour with a cocktail of SERCA pump inhibitors in order to fully

deplete SR Ca2+ stores». This is not indicated in Fig. 4.
3) The legend in Fig. 5 does not completely match the content. In addition, the numbers of significantly down-regulated proteins are not consistent in Fig. 6A-C.
4) The authors should comment on a possible connection between the muscle phenotype in the GS model and the cannibalization of the pups in crossings with female GS mice.
5) The manuscript should be carefully checked for typographical errors and inconsistencies to increase the precision level (E.g. «herteromeric ORAI1 channels», «plasma membrane» not «surface membrane», «These findings suggest compensatory changes in SOL muscles of GS mice designed limit exercise intolerance could partially explain the absence of TAs in SOL muscles», «..likely exhibit a low level of persistent muscle damage experience might experience milder muscle damage»).

** As a service to authors, EMBO Press provides authors with the possibility to transfer a manuscript that one journal cannot offer to publish to another EMBO publication or the open access journal Life Science Alliance launched in partnership between EMBO Press, Rockefeller University Press and Cold Spring Harbor Laboratory Press. The full manuscript and if applicable, reviewers' reports, are automatically sent to the receiving journal to allow for fast handling and a prompt decision on your manuscript. For more details of this service, and to transfer your manuscript please click on Link Not Available. **

Please do not share this URL as it will give anyone who clicks it access to your account.

We would like to thank the Reviewing Editor and three expert reviewers for their very thoughtful and constructive in-depth comments for improving the manuscript. As detailed in the following point-by-point author response, the revised manuscript includes multiple additional experiments, exciting new supportive results, and addresses all reviewer comments and concerns.

Summary of Major Changes to the Manuscript:
As recommended by the Reviewing Editor, Reviewer #1 (Point 2) and Reviewer #2 (Point 7), we completed additional experiments that quantify both morphological and functional changes in skeletal muscle mitochondria from 8 month old GS mice (see new Fig. 7 and Table 5). These studies include EM mitochondrial images/analyses from EDL sections, measurements of oxygen consumption and complex I-V activity from purified mitochondria, and quantification of mitochondrial membrane potential and ROS production (TMRE and MitoSOX fluorescence) in intact single FDB fibers. These comprehensive mitochondrial morphology/function studies demonstrate significant mitochondrial damage and dysfunction in skeletal muscle of 8 month old GS mice, consistent with the proteomics results summarized in Fig. 6.

The Reviewing Editor and reviewers also requested more comprehensive analyses of tubular aggregates (TAs) across different muscles, ages, and sexes. Thus, we now include new data quantifying TAs in four muscle groups (EDL, FDB, tibialis anterior, and *soleus*; Fig. 2C-G), as well as TAs in EDL muscles from 8 month old male/female mice (Fig. S4A-C) and in EDL muscles across three different ages (4 months, 8 months, and 12 months; Fig. S4D-F).

A third issue raised by the reviewers (Reviewer #1, Point 1; Reviewer #2, Point 75; Reviewer #3 main paragraph) is our justification for using V5HA-tagged ORAI1 knock-in mice to assess changes in WT ORAI1 subunit expression in GS mice when a commercially available ORAI1 antibody could potentially assess both WT and GS monomers. To answer this question, we added an additional supplementary figure (Fig. S5A) showing that in our hands, none of the commercially available ORAI1 antibodies tested, as well as our own N-terminal polyclonal antibody, are not sensitive enough to specifically detect endogenous ORAI1 expression in western blots of skeletal muscle lysates (using muscle-specific ORAI1 KO mice as a negative control). This is why we developed knock-in mice with a V5-3xHA tag added to the ORAI1 C-terminus, which at least enables quantification of WT ORAI1 subunits using a rat HA antibody (Fig. 5F-H, Fig. S5B-F).

Finally, we also completed the excellent PNGase experiments suggested by Reviewer 1. Results of these studies show that both the short and long ORAI1 isoforms are similarly reduced in muscle of GS mice (Fig. S5F). We also conducted whole blood electrolyte analyses as inferred by Reviewer 2, which found that GS mice exhibit significant hypocalcemia, similar to that observed for human TAM patients with the analogous G100S ORAI1 mutation (Fig. 1D and Fig. S2D).

Below we address all comments and suggestions raised by the Reviewing Editor and reviewers, as well as describe specific changes to the revised manuscript made in response to these issues.

Reviewing Editor Comments:

To revise your study, we ask you to strengthen the following aspects: - add further data on mitochondrial function (OXPHOS, glycolysis, metabolic enzymes) and ideally mitochondrial morphology (ref#1, point 2, ref#2, point 7).

As requested, the revised manuscript now provides new data (Fig. 7 and Table 5) where we quantify changes in mitochondrial morphology and function in skeletal muscle of 8 month old GS mice. EM images of longitudinal sections of EDL muscle show significant mitochondrial damage (defined as disrupted external membranes, vacuolation of internal membranes, and/or myelin figure formation), reduced number of mitochondria at the A band, and increased size in EDL muscle of 8M old GS mice. Clark-type electrode measurements of oxygen consumption revealed significantly reduced maximal state II respiration and respiratory control ratio (RCR) in mitochondria purified from skeletal muscle of 8 month old GS mice compared to that of age-matched WT mice, consistent with reduced mitochondrial respiratory function. We also measured other parameters of mitochondrial function, including biochemical measurements of complex I-V activity from purified mitochondria, as well as mitochondrial membrane potential (with TMRE) and the rate of mitochondrial reactive oxygen species (ROS) generation (with MitoSOX) in single intact FDB fibers. These data indicate that complex I (and III and V) activity is increased, the mitochondrial membrane potential is hyperpolarized, and ROS production is enhanced in muscle of GS mice. Since ROS generation from complex I is strongly dependent on mitochondrial membrane potential (Starkov & Fiskum, 2003), these findings are consistent with increased ROS generation from complex I in skeletal muscle of GS mice. In addition, the increase in complex III activity could also contribute to the increase in mitochondrial ROS production observed in FBD fibers of GS mice.

Transcriptomics on muscle from GS mice (referee 1, point 2) are not required. – provide a more thorough description and analysis of the tubular aggregates observed in different muscles of OraiG100S/+ mice and TAM patients (if available), along the lines suggested by referee 2. - reconcile the data on Orai1 protein downregulation as adaptive response (ref#1, ref#2, ref#3) and the observed discrepancies regarding SOCE and disease onset (ref#2, point 4).

The revised manuscript now includes more comprehensive analyses of tubular aggregates (TAs) across different muscles, ages, and sexes. Specifically, we now include new data quantifying TAs in four muscles (EDL, FDB, tibialis anterior, and *soleus*; Fig. 2C-G), as well as TAs in EDL muscles across three ages (4 months, 8 months, and 12 months; Fig. S4D-F) and in EDL muscles from 8 month old male and female mice (Fig. S4A-C). As there are only a few known families identified with this particular ORAI1 mutation and we are not a clinical lab with access to human biopsy samples, we were unable to provide parallel analyses on muscle from TAM patients. Interestingly, TAs were significantly more prevalent in GS mice at 8M and 12M of age, but not at 4M of age. These results are consistent with a slowly developing TA histopathogy in GS mice, validating the rationale for conducting most experiments in this study at 8M of age. Below, we provide explanations for the timeline of the postnatal reduction in constitutive Ca^{2+} entry with the observed reduction in SOCE across all ages and slow disease course progression.

Referee #1 Comments:

Review of Zhao et al. report a new heterozygous knock-in mouse with a point mutation in Orai1 (G100S) that shows tubular aggregate myopathy (TAM) with compromised skeletal muscle function. The gist of this study is that the authors claim to provide a model to study a form of heritable TAM in humans that is characterized with the presence of tubular aggregates, muscle weakness and exercise intolerance. They report that 8-month-old Orai1 GS+/- present with tubular aggregates, muscle weakness, exercise intolerance and elevated creatine kinase (CK) levels, phenocopying the human myopathy. The equivalent Orai1 point mutation in humans is G98S and is a gain of function (GoF) mutation with enhanced Orai1 activity under basal conditions, in the absence of store depletion. The authors report that constitutive calcium entry in the GS+/- mouse is only seen in skeletal muscle during early development but not in adult muscle and contend that this is at least partially explained by a postnatal adaptive reduction in Orai1 protein expression. Finally, the authors performed proteomic analysis on 2-week-old and 8-month-old wildtype and GS+/- mice and showed that mitochondrial-related pathways are altered in skeletal muscle of the GS+/- mouse.

This is an important manuscript that reports a novel Knock-in mouse with significant relevance to a human myopathy. The authors have also generated an HA-tagged Orai1 knock-in mouse that was crossed to the GS+/- mouse to evaluate the effect of the GS mutation on the expression of wildtype Orai1 protein. The mouse was characterized for muscle force generation as well as calcium measurements of Orai1 activity under basal and store-depletion activation in fast twitch EDL, FBD and SOL muscles.

However, the study is somewhat descriptive with a paucity of molecular insights beyond expected findings on muscle weakness and constitutive calcium activity. Interesting observations are reported that include the adaptive postnatal downregulation of wildtype Orai1 and the mitochondrial phenotype, but neither of these observations are studied in-depth to shed light on their connection to the GS mutation. This and some missing data and controls are described below:

We thank the reviewer for their acknowledgement of the relevance and novelty of our knock-in mouse model of TAM. As requested, we added new data quantifying altered mitochondrial structure and function in skeletal muscle of 8M old GS mice (new Fig. 7 and Table 5), which validate the proteomic findings of this study (Fig. 6). We also completed the PNGase studies suggested below by the reviewer, which revealed that expression of both the short and long ORAI1 isoforms are similarly reduced (~60% each) in skeletal muscle of 8 month old GS mice. These findings, in combination with the observed unaltered level of ORAI1 transcript (Fig. S6), are consistent with an increased degradation rate of heteromeric ORAI1 hexamers in GS mice, contributing to the reduction in maximal SOCE observed across all ages (see Fig. 4G).

Given the new mitochondrial data, the manuscript was revised (page 33-34, lines 861-918) to discuss prior data linking ORAI1 localization and Ca^{2+} entry with altered mitochondrial activity, as well as consideration of connections between mitochondrial dysfunction and skeletal muscle

disorders including Duchenne muscular dystrophy and cachexia (Carson et al, 2016; Padrao et al, 2013; Pant et al, 2015). As observed with TAM, these conditions are similarly reported to exhibit muscle weakness, altered SOCE activity, and increased mitochondrial damage.

1) The authors acknowledge that crossing GS and HA- mice will determine only changes in expression of wildtype Orai1 proteins, which is a weakness. How the GS mutation is affecting the expression of wildtype Orai1 and whether this also applies to the GS protein is not clear.

We understand the reviewer's concern that our approach, specifically designed to measure the expression level of wildtype (WT) ORAI1 subunits and not G100S ORAI1 subunits, is a limitation. However, our observation that both short and long WT ORAI1 isoforms are similarly reduced in muscle of GS mice without a change in ORAI1 transcript is intriguing, consistent with the observed reduction in SOCE activity across all ages and suggests that heteromeric (WT:GS) ORAI1 hexamers exhibit an enhanced rate of degradation. Ongoing studies are testing this idea but extend beyond the scope of this study.

Before we generated the ORAI1 C-terminal V5-3xHA-tagged knock-in mice, we tested multiple different commercially available ORAI1 antibodies, as well as a rabbit ORAI1 polyclonal antibody we raised against the first 20 amino acids of the ORAI1 N-terminus. Unfortunately, none of these antibodies provide sufficient sensitivity to detect endogenous ORAI1 in skeletal muscle (though they all work to varying degrees for overexpressed ORAI1) (Fig. S5A). Therefore, we generated ORAI1 V5-3xHA-tagged knock-in mice and validated that a rat-HA antibody readily and specifically detects endogenous ORAI1 in muscle and other tissues (see Fig. S5) as an alternate approach to assess WT ORAI1 subunits (both long and short isoforms). We believe that these mice will be of great value to many others in the ORAI1 field.

Orai1 mRNA is not affected, and the authors speculated about potential effects on protein trafficking and glycosylation, but none of this is substantiated by evidence. First, we know from previous studies that human Orai1 G98S locates normally to the plasma membrane and mediates activity. The presence of constitutive calcium flux in 2 weeks old (Fig 4B) is not consistent with defects in trafficking or glyosylation.

We agree with the reviewer that our data indicate that altered ORAI1 glycosylation (see new Fig. S5H) and trafficking to the T-tubule (see Fig. S7) are unlikely to be the reason for reduced SOCE activity in muscle fibers of GS mice and revised the manuscript to make this point clear. Since ORAI1 has been reported to localize to TAs in aged male mice (Boncompagni et al., 2020), we felt that it was important for us to confirm that ORAI1 T-tubule localization was not altered in muscle of GS mice.

The suggestion that the Orai1 higher band is decreased much more significantly than the shorter band is not supported by the data presented. Both looks decreased to the same extent and the final densitometry numbers provided appear to this reviewer to be likely an artifact in densitometry measurements, likely because the short Orai1 band in the wildtype is saturated (Fig 5G, H). Glycosylation is an unlikely explanation as all bands are there in both WT and GS,

just less dense in GS. One recommendation is to declycosylate the proteins samples; in this case, one should expect to see two distinct bands at 25KD and 33KD and one would expect to reliably determine whether both short and long Orai1 are decreased equally or differentially in the muscle of GS mice.

We thank the reviewer for pointing out that the upper and lower ORAI1 bands seem to be reduced to a similar extent and also for suggesting deglycosylation studies to more definitively quantify changes in the expression of the long and short ORAI1 isoforms. Thus, we quantified full-length and short ORAI1 isoforms in skeletal muscle from 8M old WT and GS mice before and after PNGase treatment to deglycosylate the proteins. These studies confirm the reviewer's point that both the short and long ORAI1 isoforms are similarly reduced ~60% in muscle of GS mice. These new data are included in the revised manuscript (see new Fig. S5F) and the Discussion section was revised accordingly.

2) The authors provide no data on mitochondrial structure, shape and morphology using TEM, including a thorough comparison of the two types of mitochondrial populations between WT and GS mice at 2 weeks and 8 months of age. They also provide no direct measurements of mitochondrial function or metabolism in general. The proteomics data provided and the alterations in mitochondrial-related pathways is quite vague and superficial. Previous studies on several cell types (T cells, B cells, astrocytes and Smooth muscle), showed that mice lacking either STIM or Orai have defective OXPHOS and glycolysis with altered expression of several metabolic enzymes and transporters (e.g. GLUT) regulating these pathways. Presumably, the decrease in Orai1 reported in the GS mouse could explain these mitochondrial alterations, but from this manuscript 8 mitochondrial-related proteins involved in lipid oxidation and OXPHOS are actually upregulated (not downregulated) and its not clear how these data can be rationalized within the current literature on Orai1 channels.

As discussed above, the revised manuscript now includes new data (Fig. 7 and Table 5) where we quantify changes in mitochondrial morphology and function in skeletal muscle of 8 month old GS mice. EM images of longitudinal sections of EDL muscle from 8M old WT and GS mice show significant mitochondrial damage (defined as disrupted external membranes, vacuolation of internal membranes, and/or myelin figure formation), reduced number of mitochondria at the A band, and increased size. Clark-type electrode measurements of oxygen consumption revealed significantly reduced maximal state II respiration and respiratory control ratio (RCR) in mitochondria purified from skeletal muscle of 8 month old GS mice compared to that from age-matched WT mice, consistent with reduced mitochondrial function. We also measured other parameters of mitochondrial function including biochemical measurements of complex I-V activity from purified mitochondria, as well as mitochondrial membrane potential (with TMRE) and the maximum rate of mitochondrial reactive oxygen species (ROS) generation (with MitoSOX) in single intact FDB fibers. These data indicate that complex I (and complex II and V) activity is increased, the mitochondrial membrane potential is hyperpolarized, and ROS production is enhanced in muscle of GS mice. Since ROS generation from complex I is strongly dependent on mitochondrial membrane potential (Starkov & Fiskum, 2003), these findings are consistent with increased ROS generation from complex I in skeletal muscle of GS mice. In

addition, the increase in complex III activity could also contribute to the increase in mitochondrial ROS production observed in FDB fibers of GS mice. These points are included in the Discussion section of the revised manuscript (page 28, lines 708-718; p 34, lines 904-918).

Have the authors considered performing transcriptomics on muscle from GS mice to contrast with previous published findings on other cell types mentioned above?

While we agree with the reviewer that transcriptomic studies of skeletal muscle from GS could not only support the proteomics results, but also provide important additional insights regarding potential changes in RNA-based regulatory mechanisms (e.g. micro-RNAs, long non-coding RNAs, etc), the time and cost of these studies extend beyond the scope of this work.

3) It is not clear why the authors do not provide direct measurements of SR calcium as was done for mitochondria.

We used an indirect approach (rapid store Ca^{2+} release with CPA/Ionomycin in 0 Ca) to assess total Ca^{2+} store content in single muscle fibers, as we have done previously (Carrell et al., 2016; Garcia-Castaneda et al., 2022; Michelucci et al., 2019; Michelucci et al., 2020). These studies found that total Ca^{2+} store content is significantly increased in muscle fibers from GS mice (Fig. S8B and E). We have previously used an SR-targeted Ca^{2+} probe (D1ER) to more directly measure SR Ca^{2+} content in single muscle fibers (Michelucci et al., 2020). However, the D1ER signal is dominated by Ca^{2+} stored within the junctional SR, and thus, does not readily assess contributions of other compartments including the longitudinal SR, mitochondria, and Ca^{2+} stored within tubular aggregates. While direct comparative measurements in each of these compartments separately would be interesting, this would involve significant assay development, optimization, and validation that would take considerably more time than was available for this resubmission.

Minor:

4) It was quite difficult to evaluate this manuscript because the literature cited in the manuscript is referenced solely with the year of publication, making difficult to identify the exact paper referenced in the list of references.

We deeply apologize for this error in formatting of the original manuscript. The revised manuscript now includes proper formatting of in-text citations and references.

5) For FTEM in Figure 2E, it is missing the wildtype controls

We thank the reviewer for this suggestion. The revised manuscript now includes representative control WT EM images in both EDL (Fig. 2C) and FDB (Fig. 2D) fibers.

6) Fig 3C, the Y axis is mislabeled as "cumulative rest"

Y axis labels in Fig. 3B and C were corrected to ‘Cumulative Rests’.

7) Lane738, lists one wrong reference (2010); it should be a 2017 reference (PMID 28219928).

We thank the reviewer for pointing out this error, which is corrected in the revised manuscript.

Referee #2 Comments:

In the present study, Zhao and colleagues describe a novel Orai1 mouse model for tubular aggregate myopathy. The article presents some novel data, but the study is largely descriptive and lacks relevant functional studies. To my opinion, the manuscript is not suitable for the EMBO Journal in its current state. Here are my comments:

Major points:

1. Although Orai1 G100S mice manifest muscle weakness, they only partially recapitulate the human disorder. The authors point out that patients with the Orai1 G100S mutation show fiber size variability, type I fiber predominance, internal nuclei and fibrosis on muscle biopsies, but none of these histological features is seen in G100S mice. Additional Orai1-typical signs like miosis, hypocalcemia, or - described in one patient with G100S mutation - bleeding diathesis are also absent, questioning the relevance of the mouse model for further physiological investigations or the development of therapies.

We agree with the reviewer that some aspects of TAM in humans (e.g. fiber size variability, type I fiber predominance, internal nuclei, fibrosis) are not fully recapitulated in 8 month old GS mice. However, as TAM is a progressive disorder, it is possible that some of these other characteristics may develop over time as these mice age. Future studies will be needed to identify later-onset phenotypes as GS mice age (e.g. from 12-24 months). We have clarified this point in the Discussion section of the revised manuscript (page 31, lines 782-789).

As pointed out by the reviewer, individuals with the G98S ORAI1 TAM mutation exhibit hypocalcemia. Thus, we conducted electrolyte analyses of whole blood from 8 month old WT and GS mice. These studies found that GS mice also exhibit significant hypocalcemia (Fig. 1D) in the absence of other major changes in blood chemistry including normal levels of sodium, potassium, glucose, and pH (Fig. S2D). These results are also presented in the Phenotyping section of the Results (page 20, lines 491-494). While we have not evaluated whether GS mice exhibit miosis, this is something that would certainly interesting to assess in the future.

While bleeding diathesis was reported for one individual with the G98S ORAI1 mutation, this phenotype is not observed in other patients with this mutation or GS mice. Thus, the absence of bleeding diathesis in GS mice is consistent with the majority of individuals with this ORAI1 variant.

2. In contrast to other existing TAM mouse models, Orai1 G100S mice exhibit tubular aggregates in muscle fibres, which is certainly an interesting point. However, aggregates were not found in the *soleus* although muscle force was reduced. What is then the relevance of the tubular aggregates and their contribution to the disease? The authors speculate that the increased resistance to fatigue of the *soleus* muscle may explain the absence of aggregates, but what is the link between both? And why is there no difference in force decline between EDL and *soleus* after tetanic stimulation? A major difference between EDL and *soleus* is the fibre type composition. EDL is a fast muscle and *soleus* a slow muscle. Are aggregates only found in type II

fibres? How about mixed muscles like the gastrocnemius? To my opinion, it would be interesting to investigate the tubular aggregates in detail. What is their composition? Is it the same as the tubular aggregates in TAM patients and the aggregates occurring during the ageing process in WT mice?

Firstly, we appreciate reviewer's acknowledgement of the importance of the GS mice being the first mouse model of TAM with TAs. As requested by the reviewers, we now provide more comprehensive detailed analyses of TAs in GS mice, including quantification of TAs across different sexes (Fig. S2A-C), ages (4, 8, and 12 months; Fig. S2D-F), and muscle groups (EDL, FDB, tibialis anterior, and *soleus*; Fig. 2C-G). Importantly, TAs present in GS mice are similar to those reported in muscle biopsies from TAM patients as they exhibit a similar honeycomb-like structure observed by EM and also stain pink with Gomori Trichrome (Fig. 2A and B).

As we observed a similar reduction in peak specific force both in muscles with (EDL) and without (*soleus*) TAs, the relevance of TAs in the disease (e.g. whether pathogenic, adaptive, or an epiphenomenon) remains unclear. Thus, the observed reduction in specific force in both EDL and *soleus* muscles of 8M GS mice likely involves alternative mechanisms. Our prior proteomic (Fig. 6) and new mitochondrial structure/function data (new Fig. 7, Table 5) suggest a potential role for alterations in energy production and bioenergetics. Thus, we speculate that TAs are not directly responsible for the observed contractile deficits, but rather represent a consequence of metabolic alterations downstream of energetic insufficiency. Moreover, as *soleus* muscles are richer in mitochondria than EDL muscles, TAs may be absent in *soleus* muscle because ATP levels are not as limiting as might be the case in more glycolytic fast-twitch muscles.

In contrast to the reviewer's comment above ('And why is there no difference in force decline between EDL and *soleus* after tetanic stimulation?'), the data in Fig. 3J and K indeed show a slower force decline (greater fatigue resistance) in *soleus* compared to EDL. While the example traces may appear to exhibit similar time courses, the time scale is 2x longer for the *soleus* muscle trace. Thus, average time to half decay is >2x slower for *soleus* (~5 seconds for WT EDL and ~13 seconds for WT *soleus*) and the maximum rate of relaxation is similarly >2x slower (~-50 mN/sec for WT EDL and ~-20 mN/sec for WT *soleus*), which are shown graphically in Fig. 3J and K.

3. It is intriguing that G100S mice are phenotypically normal at 4 months and develop a muscle phenotype at 8 months, while calcium entry is increased in muscle fibers from young mice and normal in older mice. The authors hypothesize that Orai1 expression diminishes over time to compensate calcium entry - but why does the disease then start after this compensatory and protective effect?

We thank the reviewer for raising this important question. Our results suggest that constitutive Ca^{2+} entry mediated by gain-of-function G100S ORAI1 during early muscle development (embryonic through 1-2 months postnatal) drives changes in the developmental muscle proteome that results in altered mitochondrial and bioenergetic function. Over time, this results in a slowly developing myopathy characterized by weakness, exercise intolerance, elevated CK

levels, and the formation of TAs. Consistent with this idea, we find significant constitutive Ca^{2+} entry in immature myotubes and individual myofibers during early development. In addition, proteome alterations that impact mitochondrial structure/function are already well-established as early as 2 weeks of age (Fig. 6). We propose that elimination of this constitutive Ca^{2+} entry in adult muscle fibers (e.g. ≥ 4 months of age - the precise mechanism yet to be determined) is designed to limit Ca^{2+}-induced muscle damage/necrosis, which is supported by the absence of significant muscle damage, regeneration, and central nucleation in histological analyses of adult GS muscle (see Fig. 1F). We consider these concepts in the Discussion section of the revised manuscript (p. 32, lines 820-827).

4. Figure 4: constitutive calcium entry is increased in FDB muscle fibres from embryonic development to 5 weeks of age, but SOCE is strongly decreased in the same period. Do the authors have an explanation for this contradictory result? The increased calcium entry indicates that the Orai1 channel is leaky, and the activation of SOCE through TG is expected to amplify (and not to attenuate) calcium entry and Mn2+ quenching.

Our Western blot results in 2 week and 8 month old GS mice indicate that the reduction in SOCE observed early during development and throughout adulthood is due to a marked reduction in ORAI1 expression at all ages compared to age-matched WT mice (e.g. ~40-60% at both 2 weeks and 8 months of age - Fig. 5G and H; Fig. S5F). However, in spite of this reduction in ORAI1 expression, significant constitutive Ca^{2+} entry in muscle is still observed early in development, but not in adult muscle (Fig. 4B-D). Our interpretation of these results is that this low-level expression of WT and gain-of-function GS ORAI1 subunits is responsible for the low level of constitutive Ca^{2+} entry observed early in development. If the expression was normal, then this entry would be even larger and more damaging. Moreover, we speculate that a key adaptation during development prevents or blocks this constitutive entry in adult muscle. Our data indicate that this protective adaptation does not involve complete loss of ORAI1 expression (see Fig. 5H) or mis-localization of ORAI1 channels away from the T-tubule or TAs (see Fig. S7). Alternatively, the loss of constitutive Ca^{2+} entry in adult muscle could involve an inhibitory post-translational modification, selective silencing of the GS allele in adult muscle, or recruitment of a protein that blocks constitutive channel activity. Future studies will be needed to evaluate these and other possible inhibitory mechanisms. These issues are now included in the Discussion section of the revised manuscript (page 32-33, lines 833-850).

In terms of the relative amplitudes of the maximum rate of constitutive and store-operated Ca^{2+} entry, we agree with the reviewer's suggestion that activation of SOCE is expected to further amplify Ca^{2+} entry. Indeed, in fibers from 5 week old GS mice, maximal constitutive Ca^{2+} entry rate is only ~$0.3*10^3$ count/sec, while the maximal rate of store-operated Ca^{2+} entry in fibers at this age is >$1*10^3$ count/sec (or > 3-fold higher).

5. The methods used to determine the Orai1 protein levels are very indirect and the results are not convincing. Figure S4A shows two Orai1 bands in different muscles - probably the alpha and beta isoforms. But figure S4B shows a different picture with a single intermediate band in spleen, lung, liver and kidney. What is this if not Orai1 alpha and Orai1 beta? And why is there no signal

in heart? According to the gtex database, Orai1 expression is similar in heart, kidney in liver. Has this experiment been repeated? In figure S4C, the Orai bands without PNGase treatment are again different and e.g. higher in spleen and liver than in brain and muscle.

As we knocked-in a V5-3xHA tag to the extreme ORAI1 C-terminus of the endogenous ORAI1 gene, the HA immunoblots provide a direct (not indirect) measure of WT ORAI1 subunit expression and the absence of any signal in tissues from WT mice confirm the specificity of this approach (see Fig. 5F-H and Fig. S5). The HA Western blot of different tissues from V5-3xHA mice shows multiple bands for each tissue (Fig. S5C), likely reflecting different degrees of ORAI1 alpha and beta expression, as well as different degrees of glycosylation of each isoform. Interestingly, the glycosylation patterns are somewhat similar in the different excitable tissues evaluated (e.g. skeletal muscle, brain, heart), while non-excitable tissues appear to exhibit a more dominant upper band (e.g. spleen, lung, liver kidney, and SMG). While the gtex database (an RNA database) suggests similar ORAI1 expression in heart, kidney and liver, these results do not necessarily reflect protein expression. In spite of the gtex database, our results indicate that ORAI1 protein expression is actually quite low in the heart, at least in comparison to kidney, liver, spleen, lung, SMG, and skeletal muscle (Fig. S5C, *left*). We now provide cardiac Western blot data from hearts of 3 additional mice at 5x higher protein loading where multiple ORAI1 bands between 37-50 kD (similar to muscle) are readily observed (Fig. S5C, *right*). These results demonstrate that ORAI1 protein expression in mouse heart is much lower than that observed for the other tissues tested.

In addition, IF (although only semi-quantitative) in figure S6 does not seem to indicate a reduction of Orai1 protein levels.

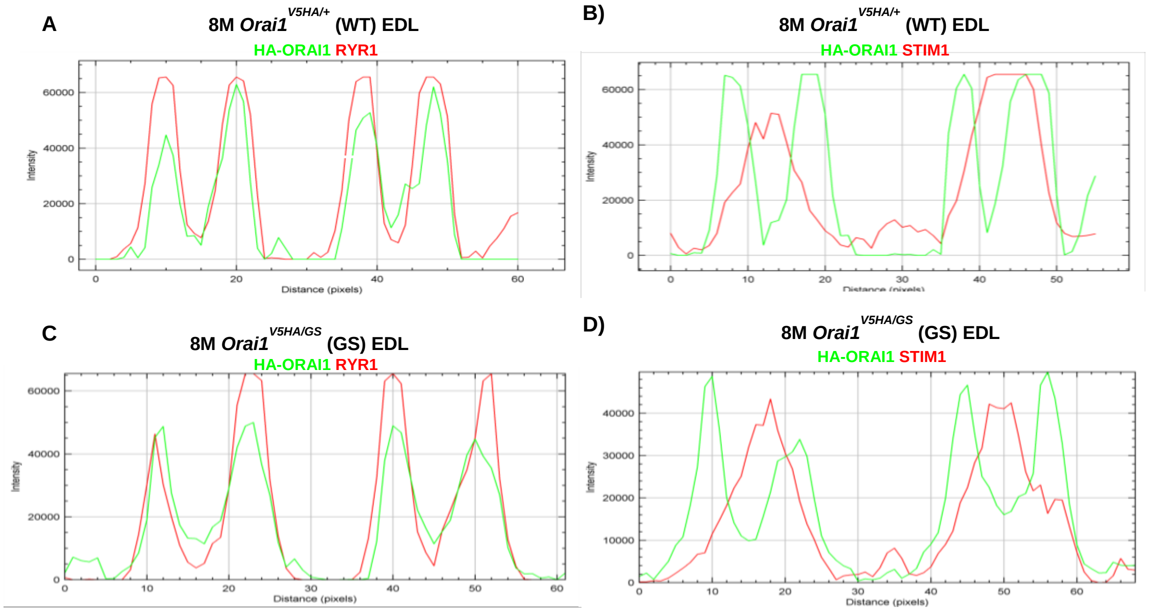

Figure A. Raw fluorescence intensity analysis of data presented in Fig. S7. Traces are plotted from two consecutive triads in EDL muscle bundle co-stained with ORAI1 and RYR1 or ORAI1 and STIM1 antibodies. Orai1 is shown in green while STIM1 or RYR1 are shown in red.

As acknowledged by the reviewer, immunofluorescence (IF) is inherently not a reliable approach to quantify protein expression in a cell. Nevertheless, we analyzed the IF images shown in Fig. S7 since they were carefully collected under identical staining, excitation, and collection conditions. Raw fluorescence intensity profiles for ORAI1 (using an HA Ab) and either RYR1 or STIM1 in two consecutive triads from the muscle fibers used in Fig. S7 are shown above in Fig. A. Average ORAI fluorescence intensity for the muscle fiber from the WT V5HA/+ mouse is ~65,000 AU, while that for the muscle fiber from the V5HA/GS mouse is only ~45,000 AU (reduced ~30%), roughly comparable to what we observe by Western blot.

It may be better to test all commercially available Orai1 antibodies (or to make one) instead of evaluating Orai1 expression through V5HA mice.

As discussed above in our response to Comment #1 of Reviewer #1, prior to generating the ORAI1 C-terminal V5-3xHA-tagged knock-in mice, we tested multiple different commercially available ORAI1 antibodies, as well as a rabbit ORAI1 polyclonal antibody against the ORAI1 N-terminus (see new Fig. S5A). Unfortunately, none of these antibodies provide sufficient sensitivity to detect endogenous ORAI1 in skeletal muscle (though they all work to varying degrees for overexpressed ORAI1). Therefore, we generated ORAI1 V5-3xHA-tagged mice and validated that a rat-HA antibody readily and specifically detects endogenous ORAI1 in skeletal muscle and other tissues (in this case using skeletal muscle from WT mice as a negative control) as an alternate approach to assess WT ORAI1 subunit expression (both long and short isoforms). We believe that these mice will be of great value to many others in the ORAI1 field.

6. In the same line, the IP results are not convincing. The authors state that only full-length Orai1 is pulled down with the N-terminal Orai1 antibody. This is not true. The western blot on figure S4D clearly shows both Orai1 bands in V5HA mice.

We apologize for the poorly worded statement in the original version of the manuscript. What we meant to state was that the N-terminal antibody only recognizes the full-length isoform. As the short and long isoforms form heterotypic hexameric channels, the N-terminal antibody can pull-down both long and short isoform subunits when they are assembled together. As pointed out by the reviewer, our pull-down studies show that while the N-terminal antibody preferentially pulls-down the full-length ORAI1, a low level of the short form is also observed, likely reflecting pull-down from heteromeric hexamers. We corrected this sentence in the revised manuscript (p. 42, lines 1095-1097).

And why is full-length Orai1 reduced 4-fold ("almost abolished") in V5HA/G100S mice although the V5HA is expected to produce 50% normal protein? Taken all WB and IP results together, it seems difficult to draw any conclusions on Orai1 protein levels.

Since *Orai1*$^{V5HA/+}$ (WT) and *Orai1*$^{V5HA/GS}$ (GS) mice both possess a single tagged WT ORAI1-V5HA allele, tissues from these mice are expected to produce 50% of tagged WT ORAI1-V5HA protein (both long and short isoforms as the tag is on the C-terminus). However,

following the suggestion of Reviewer #1, our new PNGase deglycosylation studies found a similar reduction of both ORAI1 short and long isoforms in skeletal muscle of 8 month old GS mice (Fig. S5F). In the absence of PNGase treatment (Fig. 5G and H), the higher apparent fractional reduction in ORAI1 “upper band” (representing glycosylated full-length ORAI1) compared to the “lower band” (representing glycosylated short ORAI1) in muscle of both 2 week old and 8 month old GS mice could reflect a greater relative reduction in glycosylation of full length ORAI1.

7. The proteomics data are interesting and represent a novelty. They indicate an implication of mitochondrial pathways, but no functional data are presented. To emphasize the physiological relevance of the proteomics data, it is necessary to perform additional quantitative experiments on mitochondrial respiration, and potentially also on mitochondrial fusion and fission.

As recommended by the Reviewing Editor, Reviewer #1, and Reviewer #2, we completed new experiments that quantify morphological and functional changes in skeletal muscle mitochondria from 8 month old GS mice (see new Fig. 7 and Table 5). EM images of longitudinal sections of EDL muscle from 8M old WT and GS mice show significant mitochondrial damage (defined as disrupted external membranes, vacuolation of internal membranes, and/or myelin figure formation), reduced number of mitochondria at the A band, and increased size. Clark-type electrode measurements of oxygen consumption revealed significantly reduced maximal state II respiration and respiratory control ratio (RCR) in mitochondria purified from skeletal muscle of 8 month old GS mice versus that of age-matched WT mice, consistent with reduced mitochondrial function. We also measured other parameters of mitochondrial function including biochemical measurements of complex I-V activity from purified mitochondria, as well as mitochondrial membrane potential (with TMRE) and the maximum rate of mitochondrial reactive oxygen species (ROS) generation (with MitoSOX) in single intact FDB fibers. These data indicate that complex I (and III and V) activity is increased, the mitochondrial membrane potential is hyperpolarized, and ROS production is enhanced in muscle of GS mice. Since ROS generation from complex I is strongly dependent on mitochondrial membrane potential (Starkov & Fiskum, 2003), these findings are consistent with increased ROS generation from complex I in skeletal muscle of GS mice. In addition, the increase in complex III activity could also contribute to the increase in mitochondrial ROS production observed in FBD fibers of GS mice. These points are included in the Discussion section of the revised manuscript (page 33-34, lines 861-918).

And what did proteomics reveal about Orai1 protein levels? Orai1 does not seem to appear in the downregulated proteins.

Our proteomic analyses did not identify ORAI1 protein even in muscle of WT mice, most likely due to the combination of: 1) low-level ORAI1 expression in muscle (Note: commercially available antibodies that detect overexpressed ORAI1 do not detect endogenous levels of ORAI1 in muscle) and 2) the majority of ORAI1 is transmembrane (because of their hydrophobic nature and fewer lysine and arginine residues available to generate MS-detectable peptides when digested with trypsin). All raw proteomic data will be submitted as Source Data.

Minor comments:

1. Lines 448-449: the sentence "The maximum rate of EDL force production during tetanic stimulation was also reduced significantly only in EDL muscle" is not quite clear to me.

We thank the reviewer for pointing out this lack of clarity. This statement was meant to indicate that the maximum rate of force production (dF/dt) during tetanic stimulation was significantly reduced in EDL muscle of GS mice. These data are shown as the positive dF/dt values plotted in Fig. 3E (ΔRise). To better clarify this statement, we revised this sentence as follows: 'The maximum rate of force production during tetanic stimulation (dF/dt) was also significantly reduced in EDL muscles of GS mice (Fig. 3E, *left*)' (page 22, lines 545-547).

2. The western blot in figure 5A shows two main Stim1 bands at two weeks and three main bands at 8 months. Which is the right one? Which one has been used to quantify the protein level?

For our STIM1 analyses, we only quantified the upper two bands, which correspond to the expected molecular weights for STIM1L and STIM1S in skeletal muscle. For muscle from 2 week old mice, the lowest band is likely a non-specific protein or degradation product present at this early age. This lower band was not observed in skeletal muscle from 8 month old mice.

3. Lines 537-539: The authors state that the smaller Orai1 band on figure 5G represents a combination of the glycosylated longer Orai1 isoform and the unglycosylated shorter Orai1 isoform. However, the PNGase experiments in figure S4C clearly show that unglycosylated full-length Orai1 has a higher molecular weight than the glycosylated short Orai1 isoform.

The reviewer is correct. As suggested by Reviewer #1, in order to more directly quantify expression levels of the short and long ORAI1 isoforms, we completed new PNGase deglycosylation studies using *tibialis anterior* muscle lysates from 8 month old WT and GS mice (Fig. S5F). These studies confirm the reviewer's statement that the unglycosylated full-length ORAI1 isoform has a higher molecular weight than the smaller glycosylated short ORAI1 isoform. Thus, we removed this statement in the revised manuscript.

4. The authors speculate that impaired Orai1 glycosylation contributes to the reduction of SOCE in Orai1 G100S mice. Actually, it is the inverse. Impaired Orai1 glycosylation results in channel overactivity (Dorr et al., Science Signaling 2016).

We thank the reviewer for raising this point. The impact of glycosylation on ORAI1 activity is controversial. While expression of glycosylation-deficient ORAI1 (N223A) in HEK293 cells does not alter SOCE compared to WT ORAI1 (Kilch et al., 2013), SOCE is increased in Jurkat cells (Dorr et al., 2016) and SCID fibroblasts (Gwack et al., 2007) following expression of N223A-ORAI1. Thus, the functional impact of ORAI1 glycosylation exhibits cell type specific effects. Our Western blot data show the ORAI1 glycosylation in skeletal muscle is very different from that observed in spleen, lung, kidney and other tissues. Moreover, the effect of glycosylation on ORAI1 activity on skeletal muscle has not been reported. Given all of this uncertainty, we revised the statement in the manuscript to 'altered ORAI1 post-translational modification' with no

specific reference to the type or directionality of the modification.

5. Figure S3: It is a bit surprising to see that 4 Orai1 G100S mice have the exact same number of monocytes in blood.

This apparent anomaly is due to the relatively low precision of the Heska Hematrue Veterinary Hematology Analyzer used to make these measurements coupled with the low numbers of circulating monocytes, which make up only ~1.5% of all WBCs in peripheral mouse blood. Thus, the values reported in the figure are accurate, but measured with relatively low precision. If requested, the original spreadsheet of cell count data for each mouse showing these results can be provided.

Referee #3 Comments:

The paper by Zhao et al. describes a novel mouse model that expresses Orai1 G100S, which is analogous to the GOF mutant ORAI1 G98S identified in patients presenting with Tubular Aggregate Myopathy (TAM). Similar to the Stim1 GOF models, they document muscle weakness and exercise intolerance in the GS-mouse. They also detect tubular aggregates (TAs) in fast twitch EDL and FDB muscle, and document constitutive calcium entry in FDB muscle fibers in 2 - 5wk old GS mice, which was absent in FDB fibers isolated from GS mice older than 4 months. The authors argue that the reduced Orai1 protein levels detected in tibialis anterior muscle in the GS mice may compensate for the Orai1 G100S-mediated constitutive calcium influx.

The authors present a substantial collection of results, which warrants publication, but significant revisions of the presentation are required to increase the quality of the manuscript allowing a thorough review of the manuscript.

First of all, the authors need to correct the major deficiencies in citations and the reference list, which significantly reduce the quality of the paper and impede a thorough review of the manuscript: The citations in the text consist of only the publication years and the reference list lacks journal names. These deficiencies cause challenges for the review as exemplified by a statement on page 4 «Three different STIM1 mouse models ... were previously described (2020; 2018; 2007; 2019)». There are four different Stim1 GOF mouse models described in the four years mentioned (Grosse et al. 2007, Gamage et al. 2018, Silva-Rojas et al. 2019, and Cordero - Sanchez et al. 2020). The paper by Grosse et al. 2007 lacks in the reference list. On page 28, the authors mention an additional mouse model, Stim-L96V, but such a paper lacks in the reference list. The authors describe the clinical features in patients with Stormorken syndrome, but they do not cite the original paper by Stormorken et al. (1985), and the paper from his group identifying the causal mutation in 2014.

We appreciate reviewer's acknowledgement of the importance of this new mouse model of TAM and extent of the data presented. We also thank the reviewer for pointing out the inadequacy of our in-text citation and reference formats, which are corrected in the revised manuscript. In addition, the three missing references (Grosse et al., 2007, Conte et al., 2021, and Stormorken et al., 1985) mentioned by the reviewer are now included in the revised manuscript.

The authors used a HA antibody to explore if impaired maturation or mis - localization of Orai1 caused the reduction in constitutive calcium entry and reduced Orai1 protein level in muscles in GS mice older than 4 months. However, these experiments detect only the wild type protein in Orai1-V5HA/GS mice expressing both Orai1 WT with a C - terminus HA epitope and Orai1 G100S. There are Orai1 antibodies used by other researchers that could be used to quantify the Orai1 G100S level and assess the intracellular localization.

As discussed in our reply to Comment 1 of Reviewer #1, we appreciate the reviewer's concern that our approach, which specifically measures the expression of wildtype (WT) ORAI1 subunits and not also G100S subunits, is a limitation of the study. However, our observation that both

short and long WT ORAI1 isoforms are similarly reduced in muscle of GS mice without a change in ORAI1 transcript is intriguing, consistent with the observed reduction in SOCE activity across all ages and suggests that heteromeric (WT:GS) ORAI1 hexamers exhibit an enhanced rate of degradation. Ongoing studies are testing this idea but extend beyond the scope of this study.

Before we generated the ORAI1 C-terminal V5-3xHA-tagged knock-in mice, we tested multiple different commercially available ORAI1 antibodies, as well as a rabbit ORAI1 polyclonal antibody we raised against the first 20 amino acids of the ORAI1 N-terminus. Unfortunately, none of these antibodies provide sufficient sensitivity to detect endogenous ORAI1 in skeletal muscle (though they all work to varying degrees for overexpressed ORAI1) (Fig. S5A). Therefore, we generated ORAI1 V5-3xHA-tagged knock-in mice and validated that a rat-HA antibody readily and specifically detects endogenous ORAI1 in muscle and other tissues (see Fig. S5) as an alternate approach to assess WT ORAI1 subunits (both long and short isoforms). We believe that these mice will be of great value to many others in the ORAI1 field.

Additional comments:

1) On page 16 the authors state «The number and size of TA inclusions were greater in muscles from 18M old GS mice (Fig. 2B and D)». The statement indicates a significant difference in the number and size of TA inclusions in muscles at 18M and 12M, which is not indicated in Fig. 2. Further, they claim that «TA structures in fast twitch EDL and FDB muscles (but not SOL muscles) of 8M old GS mice (Fig. 2E)». The results in SOL muscles are not included in Fig. 2.

We thank the reviewer for pointing out the lack of scientific rigor in our prior statement. In the revised manuscript, we now use EM to carefully quantify TAs (percent of fibers with TAs, number of TAs/fiber, and TA size) in EDL muscles across three different ages (4 months, 8 months, and 12 months), as well as for EDL muscles from male and female 8M old WT and GS mice. Interestingly, TAs were significantly more prevalent in GS mice at 8M and 12M of age but were not present in 4M of age. No sex differences were observed in 8M old GS mice. These results are consistent with a slowly developing TA histopathology in GS mice, thus validating the rationale for why most experiments in this study were conducted at 8M of age.

We did not observe any TAs in *soleus* muscles from 8 month old GS (or WT) mice. Since these are negative data, we do not include these results as a figure, but rather, just as a statement. However, as requested by the reviewer, we provide representative EM images of *soleus* muscles from 8 month old WT and GS mice in Fig. B below.

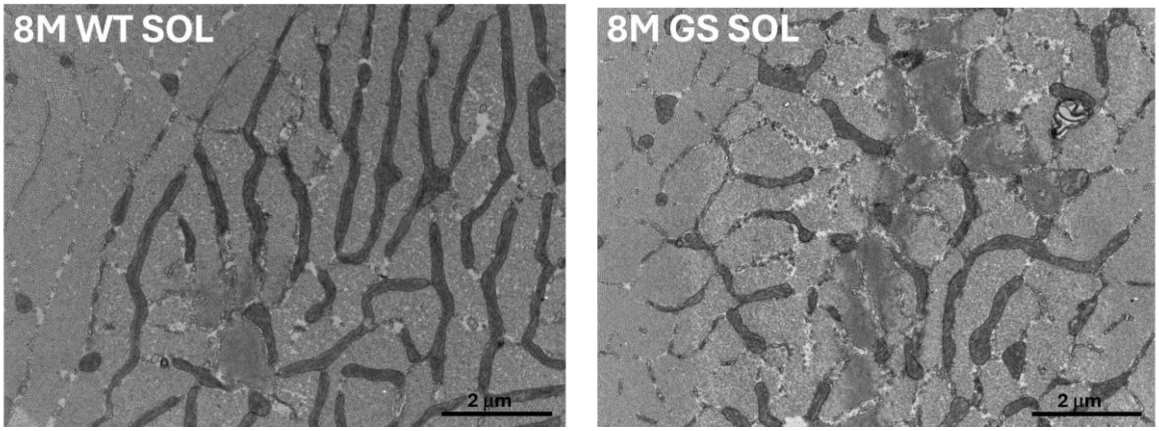

Figure B. Representative EM images of cross-sections of *soleus* muscle from 8M old WT (left) and GS (right) mice. TAs are not observed in either muscle.

2) On page 19, the authors state that «fibers were pretreated for 1 hour with a cocktail of SERCA pump inhibitors in order to fully deplete SR Ca2+ stores». This is not indicated in Fig. 4.

We appreciate the reviewer's suggestion to clarify the SERCA pump inhibitor cocktail treatment in Fig. 4. Fibers were pre-treated for 1 hour to fully deplete SR Ca^{2+} stores prior to the start of each recording. SERCA inhibitors were not included in the incubation solution used during the recordings (CPA and thapsigargin do not wash out over such a short time). Therefore, to clarify, we revised Fig. 4 to include a label under the representative SOCE traces that the fibers were pre-treated with 15 μM CPA + 1 μM Thapsigargin.

3) The legend in Fig. 5 does not completely match the content. In addition, the numbers of significantly down-regulated proteins are not consistent in Fig. 6A-C.

We thank the reviewer for pointing out the inconsistency between the legend and content in Fig. 5 and Fig. 6A-C. We corrected the figures accordingly in the revised manuscript.

4) The authors should comment on a possible connection between the muscle phenotype in the GS model and the cannibalization of the pups in crossings with female GS mice.

We thank the reviewer for inviting a comment regarding this intriguing observation and aspect of the GS mice. We agree that it would be helpful if we could identify a clear link between the GS mutation in ORAI1 and the observed cannibalization of the pups by heterozygous GS dams. However, the reason for this observed maternal infanticide is unclear. Common reasons for maternal infanticide in mice include environmental stimulations and/or altered hormone production (mostly oxytocin, progesterone and prolactin). Limited literature is available to directly associate SOCE/intracellular Ca^{2+} dynamics with murine maternal infanticide. As one possible link, SOCE is known to play a role in anterior pituitary gland function (Nunez et al., 2019), including prolactin production and oxytocin storage/release. Since alterations in prolactin and oxytocin levels have been linked to maternal infanticide in mice (McCarthy, 1990; Saltzman et al.,

2006), the cannibalization of newborn pups by GS dams could potentially reflect altered SOCE activity in the pituitary glands of these mice. However, given that this is admittedly a major speculation without any data in our study to support the idea, we did not to add this to the revised manuscript but would be willing to do so if requested by the reviewers and Reviewing Editor.

5) The manuscript should be carefully checked for typographical errors and inconsistencies to increase the precision level (E.g. «herteromeric ORAI1 channels», «plasma membrane» not «surface membrane», «These findings suggest compensatory changes in SOL muscles of GS mice designed limit exercise intolerance could partially explain the absence of TAs in SOL muscles», «..likely exhibit a low level of persistent muscle damage experience might experience milder muscle damage»)

We thank the reviewer for their careful reading of the manuscript and identification of specific errors in the text. We corrected all of the examples given above and carefully checked for typographical and grammatical errors in the revised manuscript.

Author Reply Letter References:

Boncompagni, S., Pecorai, C., Michelucci, A., Pietrangelo, L., & Protasi, F. (2020). Long-Term Exercise Reduces Formation of Tubular Aggregates and Promotes Maintenance of Ca(2+) Entry Units in Aged Muscle. *Front Physiol*, *11*, 601057. https://doi.org/10.3389/fphys.2020.601057

Carrell, E. M., Coppola, A. R., McBride, H. J., & Dirksen, R. T. (2016). Orai1 enhances muscle endurance by promoting fatigue-resistant type I fiber content but not through acute store-operated Ca2+ entry. *FASEB J*, *30*(12), 4109-4119. https://doi.org/10.1096/fj.201600621R

Dorr, K., Kilch, T., Kappel, S., Alansary, D., Schwar, G., Niemeyer, B. A., & Peinelt, C. (2016). Cell type-specific glycosylation of Orai1 modulates store-operated Ca2+ entry. *Sci Signal*, *9*(418), ra25. https://doi.org/10.1126/scisignal.aaa9913

Garcia-Castaneda, M., Michelucci, A., Zhao, N., Malik, S., & Dirksen, R. T. (2022). Postdevelopmental knockout of Orai1 improves muscle pathology in a mouse model of Duchenne muscular dystrophy. *J Gen Physiol*, *154*(9). https://doi.org/10.1085/jgp.202213081

Gwack, Y., Srikanth, S., Feske, S., Cruz-Guilloty, F., Oh-hora, M., Neems, D. S., Hogan, P. G., & Rao, A. (2007). Biochemical and functional characterization of Orai proteins. *J Biol Chem*, *282*(22), 16232-16243. https://doi.org/10.1074/jbc.M609630200

Kilch, T., Alansary, D., Peglow, M., Dorr, K., Rychkov, G., Rieger, H., Peinelt, C., & Niemeyer, B. A. (2013). Mutations of the Ca2+-sensing stromal interaction molecule STIM1 regulate Ca2+ influx by altered oligomerization of STIM1 and by destabilization of the Ca2+ channel Orai1. *J Biol Chem*, *288*(3), 1653-1664. https://doi.org/10.1074/jbc.M112.417246

McCarthy, M. M. (1990). Oxytocin inhibits infanticide in female house mice (Mus domesticus). *Horm Behav*, *24*(3), 365-375. https://doi.org/10.1016/0018-506x(90)90015-p

Michelucci, A., Boncompagni, S., Pietrangelo, L., Garcia-Castaneda, M., Takano, T., Malik, S., Dirksen, R. T., & Protasi, F. (2019). Transverse tubule remodeling enhances Orai1-dependent Ca(2+) entry in skeletal muscle. *Elife*, *8*. https://doi.org/10.7554/eLife.47576

Michelucci, A., Boncompagni, S., Pietrangelo, L., Takano, T., Protasi, F., & Dirksen, R. T. (2020). Pre-assembled Ca2+ entry units and constitutively active Ca2+ entry in skeletal muscle of calsequestrin-1 knockout mice. *J Gen Physiol*, *152*(10). https://doi.org/10.1085/jgp.202012617

Nunez, L., Bird, G. S., Hernando-Perez, E., Perez-Riesgo, E., Putney, J. W., Jr., & Villalobos, C. (2019). Store-operated Ca(2+) entry and Ca(2+) responses to hypothalamic releasing hormones in anterior pituitary cells from Orai1-/- and heptaTRPC knockout mice. *Biochim Biophys Acta Mol Cell Res*, *1866*(7), 1124-1136. https://doi.org/10.1016/j.bbamcr.2018.11.006

Saltzman, W., Ahmed, S., Fahimi, A., Wittwer, D. J., & Wegner, F. H. (2006). Social suppression of female reproductive maturation and infanticidal behavior in cooperatively breeding Mongolian gerbils. *Horm Behav*, *49*(4), 527-537. https://doi.org/10.1016/j.yhbeh.2005.11.004

Starkov, A. A., & Fiskum, G. (2003). Regulation of brain mitochondrial H2O2 production by membrane potential and NAD(P)H redox state. *J Neurochem*, *86*(5), 1101-1107. https://doi.org/10.1046/j.1471-4159.2003.01908.x

Dear Bob,

Congratulations on a great revision! Overall, the referees have been positive. However there remain some concerns that we ask you to (non-experimentally) address in a new revision. In addition to the comments below, Referee 1 has contributed the additional comments related to the Western data:

"Having extensive experience with long/short Orai1, its glycosylation, expression and detection by Westerns, I am satisfied with the authors' answer. This paper has novelty, but is obviously not perfect... No paper is!"

When you submit your revised version, please also take care of the following editorial items and add this also to your point-by-point response:

1. Please upload an author checklist found on our author guide website.

2. Please upload figures as high res figure files. Up to 5 suppl. figures can be made EV figures with the nomenclature "Figure EV1" etc., the remaining ones should be compiled in an appendix PDF with the legends underneath the figures and the nomenclature "Appendix Figure S1" etc. The appendix file will need a table of contents including page numbers.

3. Please include up to five keywords, which may or may not appear in the title, should be given in alphabetical order, below the abstract, each separated by a slash (/).

4. Please include author Jennifer Leigh into our online eJP system.

5. Please rename the data availability section to "Data Availability" section. Also, we do not allow datasets available upon request and rather require that datasets are made freely accessible.

6. Please merge the Acknowledgement section with the funding section and ensure the funding is also uploaded to our online eJP site.

7. Please remove the author contribution section from the main manuscript.

8. Please rename the conflict of interest section to Disclosure and Competing Interests section.

9. Please ensure that the references fit the EMBO style, no more than 10 author names listed before et al and remove DOIs.

10. We require the publication of source data, particularly for electrophoretic gels and blots and graphs, with the aim of making primary data more accessible and transparent to the reader. It would be great if you could provide me with a PDF file per figure that contains the original, uncropped and unprocessed scans of all or key gels used in the figure or for graphs, an Excel spreadsheet with the original data used to generate the graphs. The PDF files should be labeled with the appropriate figure/panel number, and should have molecular weight marker; further annotation could be useful but is not essential. The PDF files will be published online with the article as supplementary "Source Data" files.

11. Please provide a reagent table as described in our author guide.

12. We do not allow references to data not shown. Please remove this language from p21, p27, and p38.

13. We include a synopsis of the paper (see http://emboj.embopress.org/). Please provide me with a general summary statement and 3-5 bullet points that capture the key findings of the paper.

14. We also need a summary figure for the synopsis. The size should be 550 wide by 200-440 high (pixels). You can also use something from the figures if that is easier.

15. Please correct the section order to: Abstract, Keywords, Introduction, Results, Discussion, Methods, Acknowledgements, Disclosure and competing interests statement, References, Figure legends, Tables and their legends, Expanded View Figure legends

16. Please note that the specific URLs for PXD053472 dataset is not provided in the data availability statement.

17. Please note that reviewer access code for PXD053472 dataset is not provided in the data availability statement.

18. Figure legends: Please note that the legend of figure 4g is mislabeled as figure 4d in the manuscript. This needs to be rectified.

19. Please note that the exact p values are not provided in the legends of figures 1c-e; 2e-g; 3a-k; 4d, g; 5g-h; 7b-f, h-i.

20. Please indicate the statistical test used for data analysis in the legends of figures 1c-e; 2e-g; 3a-k; 4d, g; 5g-h; 7b-f, h-i.

21. Please note that information related to n is missing in the legends of figures 1c-e, g-k; 2e-g; 3a-k; 4a; 5a-b, g-h; 7b-c, e-f, h-i.

22. Please note that the error bars are not defined in the legends of figures 1c-e; 7b-f, h-i.

23. Please note that axis gap is not labeled appropriately in figure 1f.

I thank you for the opportunity to consider your work for publication. I look forward to your revision.

Warm wishes,
Kelly

Kelly M Anderson, PhD
Editor, The EMBO Journal
k.anderson@embojournal.org

Further information is available in our Guide For Authors: https://www.embopress.org/page/journal/14602075/authorguide

Use the link below to submit your revision:

https://emboj.msubmit.net/cgi-bin/main.plex

Referee #1:

Responsive revision. No further comments.

Referee #2:

In the revised version of the manuscript, the authors addressed all my concerns, performed additional experiments, and increased the overall quality of the article. However, the work remains largely descriptive and there are still a couple of open questions. Here are my comments:

Major points:
1) I understand the necessity to generate Orai1V5HA mice due to the absence of suitable Orai1 antibodies, and the authors state that these mice have a similar physical appearance, body mass and SOCE activity as WT animals. However, GS mice also have a similar appearance, and I think that the V5HA mice should undergo additional muscle force experiments to clearly show that the HA tag does not interfere with Orai1 function.
2) I am still not convinced by the Western blot data, and to my opinion, the experiments should be repeated or - in parts - removed from the manuscript. Why are the CASQ1 and STIM1 bands different at 2 weeks and 8 months in figures 5A and 5B? The CASQ1 signal is much weaker at 8 months (which is unexpected) and it is still not clear to me which STIM1 band is the right one. On the WB at 2 weeks, there is a band at ~110 kDa, a double band at ~80 kDa, and a lower band at ~70 kDA. In the response to my comments, the authors state that they quantified the upper two bands. Do they mean the ~110 kDa band or the ~80 kDa double band or both? At 8 months, there seem to be two bands above 100 kDa and only one below 100 kDa. I am also wondering how the (very weak and blurry) upper Orai1 band in figures 5G and 5H was quantified. Most importantly, the Orai1 bands are quite inconsistent across the analyzed tissues. The ~50 kDa and 37 KDa bands correspond to both known ORAI1 isoforms shown in figure S5F, and bands of smaller size corresponding to unglycosylated isoforms appear after PNGase treatment. However, there is only a single Orai1 band in spleen and lung in figure S5C (unglycosylated larger Orai1 isoform?), but the IP data in figure S5E show two Orai1 bands in spleen. In figure S5D, the Orai1 band of the unglycosylated smaller isoform appears lower in heart than in the other tissues. The authors state that "similar HA-reactive bands were also observed in ... multiple other tissues ...", but I have the feeling that these bands are not similar at all.
3) I stumbled over another article on the same GS mouse on BioRxiv with roughly the same authors: "Voluntary wheel running mitigates disease in an Orai1 gain-of-function mouse model of tubular aggregate myopathy". I am wondering about the novelty of the present manuscript and the potential redundancy of both articles. Indeed, both the present manuscript and the BioRvix article describe experiments on tubular aggregates, cellular calcium content, proteomics, and mitochondrial function in Orai1 GS muscle.

Minor points:
1) Line 526: the sentence "These findings were corroborated by quantitative EM measurements of honeycomb-like TA structures in fast twitch EDL, FDB and tibialis anterior sections of 8 months old GS mice (Fig 2C, D)" is a bit misleading since tibialis anterior is not shown in figures 2C and 2D.
2) I am wondering about the absence of tubular aggregates in fast-twitch EDL muscles from 18-months-old WT mice. From the literature, it seems that TAs occur in normal mice with age and are abundant in fast-twitch muscles at 18 months.
3) Figure 3F: EDL muscles from 8-months-old GS and WT mice show different curve shapes after repetitive stimulations, which is an interesting finding. The authors state that this is due to a lack of SOCE in GS mice at that age. However, SOCE was measured in FDB fibers (figure 4G) and not in EDL. Moreover, SOCE was measured after induced calcium store depletion, which was not performed for the experiments shown in figure 3F. These are quite different experimental conditions.
4) The authors observed an exercise intolerance of GS mice upon rotarod and treadmill tests. How is this compatible with the increased resistance to fatigue of EDL and SOL muscles after repetitive and sustained stimulation (figure 3)?
5) It is somewhat intriguing that myotubes and myofibers from young mice show increased constitutive calcium entry although the resting calcium levels and the expression levels of PMCA and NCX remain normal. This may be explained by the increased calcium content in the SR, but SERCA expression is normal. Did the proteomics data reveal abnormal protein levels of SERCA regulators like sarcolipin?
6) The authors observed an elevated calcium store content in FDB fibers from GS mice and suggest that this may be due to sequestered calcium in the tubular aggregates. But are tubular aggregates integral parts of the reticulum and do they release calcium?
7) Figures 8D and S8E: in myofibers from 8-months-old GS mice, resting calcium levels were normal and the total calcium store content was elevated. The authors speculate that this may be "explained by a reduction in Ca2+ efflux mechanisms (i.e. PMCA and NCX)". However, PMCA and NCX are both located at the plasma membrane. How does a reduction of PMCA and NCX influence the calcium content in organelles?
8) Proteomics on GS muscle samples showed the dysregulation of numerous mitochondrial proteins. This is actually quite common in muscle disorders and probably not specifically associated with the Orai1 mutation. This is supported by the fact that mitochondrial pathways were altered at both analyzed GS ages although constitutive calcium entry was only observed at younger ages.
9) It is not quite clear to me whether there are 3 or 4 STIM1 mouse models. The introduction cites 4 references, but the results and discussion only 3.
10) Discussion: To my opinion, the sentence "...suggesting a potential role of TAs in overall health and quality of life" is misleading. First of all, TAs are restricted to skeletal muscle and in addition, STIM1 mice do not show tubular aggregates and manifest a more severe phenotype than GS mice.

Referee #3:

The paper by Zhao et al. describes a mouse model expressing Orai1 G100S, which is analogous a GOF mutation identified in TAM patients. They document muscle weakness, exercise intolerance as well as tubular aggregates in fast twitch EDL and FDB muscle. The constitutive calcium entry documented in FDB muscle fibers in 2-5wk old GS mice, however, was absent in mice older than 4 months, which may be explained by the reduced Orai1 protein levels suggesting a compensation mechanism. The paper contains a substantial collection of results, which warrants publication, but some improvements are needed to increase the quality of the manuscript:

In line 83-85, the authors state that «Three different STIM1 mouse models were previously described...». There are four different Stim1 GOF mouse models described: Stim1-D84G (Grosse et al. 2007), Stim1-R304W (Gamage et al. 2018), Stim1-R304W (Silva-Rojas et al. 2019), and Stim1-I115F (Cordero-Sanchez et al. 2020). Even though the two Stim1-R304W-lines show overlapping muscle phenotypes, they also show differences e.g. concerning thrombocytopenia and anemia. This is incorrectly described (lines 510-513, 747-750 and 788-790) and should be corrected. Moreover, the reported protective reduction of STIM1 protein expression in skeletal muscle (lines 852-854) should cite the correct paper.

Also in line 891, the authors mention an additional mouse model, Stim1-L96V, but they refer to Conte et al., 2021, who presented experiments in STIM1 L96V-patient-derived skeletal muscle cells.

In line 474-476, the authors claim that that crossing female GS mice with male WT mice resulted in parental infanticide immediately after birth. In order to include this result, the authors need to report the numbers of crossings performed.

The manuscript would still benefit from carefully check for typographical errors.

We would like to thank the Reviewing Editor and three reviewers for their appreciation of the additions and improvement of the revised manuscript and also for they thoughtful and constructive additional suggestions for improving the manuscript. As detailed in the following point-by-point author response, the manuscript was revised to address all reviewer comments and concerns.

Referee #1
No comments

Referee #2
Major points:
1) I understand the necessity to generate Orai1V5HA mice due to the absence of suitable Orai1 antibodies, and the authors state that these mice have a similar physical appearance, body mass and SOCE activity as WT animals. However, GS mice also have a similar appearance, and I think that the V5HA mice should undergo additional muscle force experiments to clearly show that the HA tag does not interfere with Orai1 function.
While we appreciate the reviewer’s concern on a theoretical basis, we not only reported that the V5HA mice exhibit normal size/weight, but we also showed thar muscle fibers from these mice exhibit normal SOCE function, magnitude, Orai1 localization, and Orai1 expression (Fig. 5C-F).

2) I am still not convinced by the Western blot data, and to my opinion, the experiments should be repeated or - in parts - removed from the manuscript. Why are the CASQ1 and STIM1 bands different at 2 weeks and 8 months in figures 5A and 5B? The CASQ1 signal is much weaker at 8 months (which is unexpected) and it is still not clear to me which STIM1 band is the right one. On the WB at 2 weeks, there is a band at ~110 kDa, a double band at ~80 kDa, and a lower band at ~70 kDA. In the response to my comments, the authors state that they quantified the upper two bands. Do they mean the ~110 kDa band or the ~80 kDa double band or both? At 8 months, there seem to be two bands above 100 kDa and only one below 100 kDa.
Quantitative comparisons regarding relative Stim1 and Casq1 expression between 2 weeks old mice and 8 months old mice cannot be inferred from the data shown in our original Fig. 5A and B since the 2 week and 8 months old samples were run on separate gels, at different times, and scanned with different exposures. In order to make a direct quantification, we re-ran 2 weeks and 8 months old samples on the same gel (separate gels for Stim1 and Casq1) and quantified bands from a single scan. The results confirm what we originally concluded (i.e. no significant difference in Stim1 or Casq1 expression level in TA muscle of GS mice at either age), but now enable direct quantitative comparison of relative expression levels at 2 weeks and 8 months of age. We replaced prior Fig. 5A and B with these new data in the revised manuscript.

I am also wondering how the (very weak and blurry) upper Orai1 band in figures 5G and 5H was quantified. Most importantly, the Orai1 bands are quite inconsistent across the analyzed tissues. The ~50 kDa and 37 KDa bands correspond to both known ORAI1 isoforms shown in figure S5F, and bands of smaller size corresponding to unglycosylated isoforms appear after PNGase treatment. However, there is only a single Orai1 band in spleen and lung in figure S5C (unglycosylated larger Orai1 isoform?), but the IP data in figure S5E show two Orai1 bands in spleen. In figure S5D, the Orai1 band of the unglycosylated smaller isoform appears lower in heart than in the other tissues. The authors state that "similar HA-reactive bands were also observed

in ... multiple other tissues ...", but I have the feeling that these bands are not similar at all.

For analysis of upper and lower Orai1 regions, the exact areas of analysis are shown in the example image to the right. The inconsistency in the Orai1 bands across tissues in SFig. 5F is due to differential glycosylation of each of the two different Orai1 isoforms (as well as different relative expression of the two isoforms in each tissue). Consistent with this, following the suggestion of Rev 1, deglycosylation with PNGase treatment reduces the complex glycosylation smears (or “blurry” bands) in each tissue down to two discrete bands that are similar across all tissues (see the Orai1$^{V5HA/+}$ PNGase-treated TA-P, Brain-P, Liver-P, and Spleen-P lanes in SFig. 5D). These PNGase experiments demonstrate that the size of the two core deglycosylated Orai1 long and short isoforms are the same across all tissues. We think that this is quite convincing with the new PNGase data.

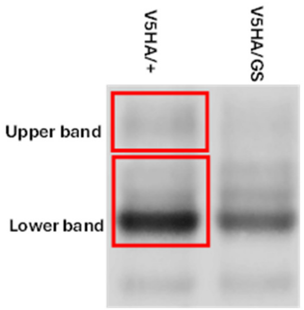

In spite of that stated above by the reviewer, the data in SFig. 5D does not include heart tissue (only skeletal muscle brain, spleen and liver). As for spleen, both upper and lower HA-reactive bands are clearly seen in SFig. 5C, though relative expression of the lower band in spleen (also lung, pancreas, and SMG) is considerably less than that observed in muscle, brain, liver and kidney. Reduced relative expression of the lower band in spleen is corroborated by the IP data shown in SFig. 5E (Note: Prior SFig. 5 is now EV-Fig. 4). Differences in relative long and short isoform expression likely reflects differential levels OF tissue-specific Orai1 alternative translation.

Importantly, we demonstrated numerous times the specificity of the rat HA antibody by using muscles lysates from WT mice (Orai1+/+) as controls (see Fig. 5F and SFig. 5B-E). The absence of detectable bands in muscle lysates from WT mice demonstrates that the bands observed in muscle lysates from V5HA-Orai1 mice can only originate from the V5HA-tagged Orai1 protein (which also run at the appropriate molecular wight). WT control mice were used as negative controls since these blots to demonstrate that the rat HA antibody does not detect the untagged Orai1 protein. In fact, these blots also show that the rat HA antibody does not significantly detect any other muscle protein at the dilution and exposure level used. As an alternative confirmation, we tested the rat HA antibody on muscle lysates WT mice (Orai1$^{+/+}$), V5HA/+ mice (Orai1$^{V5HA/+}$), and muscle-specific Orai1 knockout (Orai1$^{-/-}$) mice. These results confirm the lack of reactivity of the rat HA antibody also in muscle lysates from Orai1-/- mice.

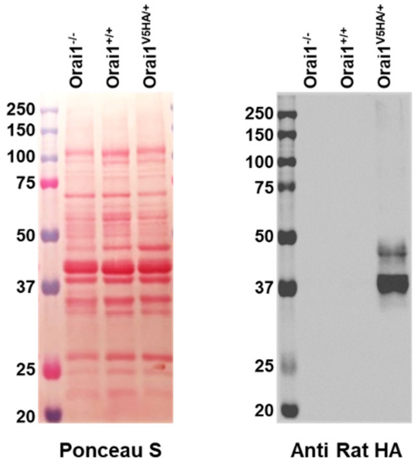

3) I stumbled over another article on the same GS mouse on BioRxiv with roughly the same authors: "Voluntary wheel running mitigates disease in an Orai1 gain-of-function mouse model of tubular aggregate myopathy". I am wondering about the novelty of the present manuscript and the potential redundancy of both articles. Indeed, both the present manuscript and the BioRvix article describe experiments on tubular aggregates, cellular calcium content, proteomics, and mitochondrial function in Orai1 GS muscle.

We thank the reviewer raising this point. The manuscript in *BioRxiv* is a completely different study

where we “treated” WT and GS mice with 6 months of voluntary wheel running (VWR) exercise. As an intervention study, all analyses involved comparing results from sedentary (mouse singly housed in wheel cages with locked wheels, compared to communally housed in this manuscript) and VWR mice (singly housed in wheel cages with unlocked wheels). Thus, though the sedentary control studies in this study be similar to those reported in the current manuscript, they were collected as a completely separate dataset under different conditions. Every experiment in that manuscript assesses the impact of 6 months of VWR on each outcome variable. Results from the current manuscript under consideration provide baseline data for that follow-up intervention study. Also, to clarify, the *bioRxiv* manuscript ***does not*** include any mitochondrial function studies – all of these studies were initiated during the resubmission of this manuscript, well after the *bioRxiv* manuscript was deposited. (Note: the VWR study was deposited in *bioRxiv* before this submission in order to accommodate the lead student on that project, Tom O’Connor, who needed to graduate with this first-author submission).

Minor points:
1) Line 526: the sentence "These findings were corroborated by quantitative EM measurements of honeycomb-like TA structures in fast twitch EDL, FDB and tibialis anterior sections of 8 months old GS mice (Fig 2C, D)" is a bit misleading since tibialis anterior is not shown in figures 2C and 2D.
Corrected.

2) I am wondering about the absence of tubular aggregates in fast-twitch EDL muscles from 18-months-old WT mice. From the literature, it seems that TAs occur in normal mice with age and are abundant in fast-twitch muscles at 18 months.
The reviewer is correct that TAs can be observed at a low frequency in EDL muscles from 18M old male, but not female, WT mice. While we show a representative Gomori image of a WT EDL muscle with no TAs (which is what is most typically observed at this age), we did not compare and quantify the frequency of TAs at this age (we do compare/quantify TAs in EDL muscles at 4, 8, and 12 months – ages where TAs are very infrequent in WT mice). We revised the statement in the manuscript to indicate that the 18M image shown in Fig. 2B is a representative image.

3) Figure 3F: EDL muscles from 8-months-old GS and WT mice show different curve shapes after repetitive stimulations, which is an interesting finding. The authors state that this is due to a lack of SOCE in GS mice at that age. However, SOCE was measured in FDB fibers (figure 4G) and not in EDL. Moreover, SOCE was measured after induced calcium store depletion, which was not performed for the experiments shown in figure 3F. These are quite different experimental conditions.
While we agree with the reviewer, we previously reported in prior publications that the increase in specific force seen during the initial 10-15 repetitive high frequency stimulation trains (or “bump phase”) in EDL muscles strongly correlates with interventions that either reduce/block SOCE in FDB fibers (e.g. Orai1 KO, transgenic expression of dnOrai1, addition of Orai1 inhibitors like Btp2, or removal of extracellular calcium) or enhance SOCE (acute exercise, long term voluntary wheel running, Casq1 KO) (Boncompagni *et al*, 2018; Michelucci *et al*, 2019; Michelucci *et al*, 2020; O'Connor *et al*, 2023). Thus, our findings that both SOCE in FDB fibers and the “bump phase” of specific force production during the initial 10-15 repetitive high frequency stimulation trains in EDL

muscle are essentially abolished in GS mice is entirely consistent with our prior published findings.

4) The authors observed an exercise intolerance of GS mice upon rotarod and treadmill tests. How is this compatible with the increased resistance to fatigue of EDL and SOL muscles after repetitive and sustained stimulation (figure 3)?

We are not entirely sure why this is the case, but we assume that prolonged (e.g. 1 hour duration) *in vivo* treadmill and rotarod behavioral studies more complex, extensive, and involve the activity of multiple muscle groups than acute (150 seconds) repetitive high frequency stimulation studies conducted on excised EDL muscles.

5) It is somewhat intriguing that myotubes and myofibers from young mice show increased constitutive calcium entry although the resting calcium levels and the expression levels of PMCA and NCX remain normal. This may be explained by the increased calcium content in the SR, but SERCA expression is normal. Did the proteomics data reveal abnormal protein levels of SERCA regulators like sarcolipin?

The reviewer raises an interesting question. We checked our proteomic database and sarcolipin was not detected in TA muscles from either WT or GS mice at 2 weeks of age or 8 months of age. This is not unexpected since sarcolipin is primarily expressed only in slow twitch muscle (e.g. *soleus*) in mice and we have not been able to detect sarcolipin in western blots of TA muscle.

6) The authors observed an elevated calcium store content in FDB fibers from GS mice and suggest that this may be due to sequestered calcium in the tubular aggregates. But are tubular aggregates integral parts of the reticulum and do they release calcium?

Our measurements of total Ca^{2+} store content were assessed by addition of ionomycin (together with CPA SERCA inhibitor in 0 Ca Ringers or "ICE"). ICE releases Ca^{2+} from all intracellular Ca^{2+} storage compartments, which include SR, mitochondria, vesicles, and lysosomes. In normal skeletal muscle, this signal primarily originates from Ca^{2+} stored within the SR, which is the predominant intracellular Ca^{2+} storage compartment of skeletal muscle. However, if TAs provide an additional intracellular Ca^{2+} storage compartment as others have suggested, then this could explain in part the larger Ca^{2+} store content observed in TA muscle from 8M old GS mice.

7) Figures 8D and S8E: in myofibers from 8-months-old GS mice, resting calcium levels were normal and the total calcium store content was elevated. The authors speculate that this may be "explained by a reduction in Ca2+ efflux mechanisms (i.e. PMCA and NCX)". However, PMCA and NCX are both located at the plasma membrane. How does a reduction of PMCA and NCX influence the calcium content in organelles?

We removed this speculation from the revised manuscript. The sentence was revised to simply state the findings as follows: "Similarly, the resting Ca^{2+} concentration was also unaltered (Fig. EV5D) and total Ca^{2+} store content was significantly elevated (Fig. EV5E) in muscle fibers from 8M old GS mice, while Ca^{2+} efflux mechanisms were reduced (i.e. PMCA and NCX expression) (Fig. EV5F).

8) Proteomics on GS muscle samples showed the dysregulation of numerous mitochondrial proteins. This is actually quite common in muscle disorders and probably not specifically associated with the Orai1 mutation. This is supported by the fact that mitochondrial pathways

were altered at both analyzed GS ages although constitutive calcium entry was only observed at younger ages.
We agree with the reviewer. This is mentioned in the Discussion section (e.g. lines 548-551).

9) It is not quite clear to me whether there are 3 or 4 STIM1 mouse models. The introduction cites 4 references, but the results and discussion only 3.
As pointed out by Reviewer #3, four different STIM1 TAM knock-in mouse models have been generated, but two of these models involve the same Stim1 variant (R304W). We revised the manuscript throughout to more accurately reflect the Stim1 TAM models in the published literature.

10) Discussion: To my opinion, the sentence "...suggesting a potential role of TAs in overall health and quality of life" is misleading. First of all, TAs are restricted to skeletal muscle and in addition, STIM1 mice do not show tubular aggregates and manifest a more severe phenotype than GS mice.
We agree with the reviewer, and thus, removed this phrase from the sentence in question.

Referee #3
In line 83-85, the authors state that «Three different STIM1 mouse models were previously described...». There are four different Stim1 GOF mouse models described: Stim1-D84G (Grosse et al. 2007), Stim1-R304W (Gamage et al. 2018), Stim1-R304W (Silva-Rojas et al. 2019), and Stim1-I115F (Cordero-Sanchez et al. 2020). Even though the two Stim1-R304W-lines show overlapping muscle phenotypes, they also show differences e.g. concerning thrombocytopenia and anemia. This is incorrectly described (lines 510-513, 747-750 and 788-790) and should be corrected. Moreover, the reported protective reduction of STIM1 protein expression in skeletal muscle (lines 852-854) should cite the correct paper.
We thank the reviewer for raising these important points. We revised the manuscript (prior lines 83-85, 510-513, 747-750 and 788-790) to more accurately reflect the published literature, including differences between the blood-related phenotypes of the two Stim1-R304W mouse lines. We also revised the manuscript (prior lines 852-854) to cite the correct reference regarding the protective reduction in Stim1 protein expression in homozygous Stim1-R304W mice.

Also in line 891, the authors mention an additional mouse model, Stim1-L96V, but they refer to Conte et al., 2021, who presented experiments in STIM1 L96V-patient-derived skeletal muscle cells.
Thank you for pointing out this error (i.e. Conte et al., 2021 studied L96V patient derived muscle cells not L96V knock-in mice). We corrected this in the revised manuscript (lines 532-534).

In line 474-476, the authors claim that that crossing female GS mice with male WT mice resulted in parental infanticide immediately after birth. In order to include this result, the authors need to report the numbers of crossings performed.
We observed this for 16 different successful crosses with female GS mice. This is now included in the revised manuscript (lines 124-126).

The manuscript would still benefit from carefully check for typographical errors.
We carefully reviewed and corrected the manuscript for typographical errors.

References:

Boncompagni S, Michelucci A, Pietrangelo L, Dirksen RT, Protasi F (2018) Addendum: Exercise-dependent formation of new junctions that promote STIM1-Orai1 assembly in skeletal muscle. *Sci Rep* 8: 17463

Michelucci A, Boncompagni S, Pietrangelo L, Garcia-Castaneda M, Takano T, Malik S, Dirksen RT, Protasi F (2019) Transverse tubule remodeling enhances Orai1-dependent Ca(2+) entry in skeletal muscle. *Elife* 8

Michelucci A, Boncompagni S, Pietrangelo L, Takano T, Protasi F, Dirksen RT (2020) Pre-assembled Ca2+ entry units and constitutively active Ca2+ entry in skeletal muscle of calsequestrin-1 knockout mice. *J Gen Physiol* 152

O'Connor TN, Zhao N, Orciuoli HM, Brasile A, Pietrangelo L, He M, Groom L, Leigh J, Mahamed Z, Liang C *et al* (2023) Voluntary wheel running mitigates disease in an Orai1 gain-of-function mouse model of tubular aggregate myopathy. *bioRxiv*

Point-by-point Author Responses to Editor Queries:

1. Please upload an author checklist found on our author guide website.
UPDATED

2. Please upload figures as high res figure files. Up to 5 suppl. figures can be made EV figures with the nomenclature "Figure EV1" etc., the remaining ones should be compiled in an appendix PDF with the legends underneath the figures and the nomenclature "Appendix Figure S1" etc. The appendix file will need a table of contents including page numbers.
EV1–S1; EV2–S2; EV3–S4; EV4–S5; EV5–S8
Appendix Fig. S1–S3; Appendix Fig. S2–S6; Appendix Fig. S3–S7; Appendix Fig. S4–S9

3. Please include up to five keywords, which may or may not appear in the title, should be given in alphabetical order, below the abstract, each separated by a slash (/).
UPDATED

4. Please include author Jennifer Leigh into our online eJP system.
UPDTAED

5. Please rename the data availability section to "Data Availability" section. Also, we do not allow datasets available upon request and rather require that datasets are made freely accessible.
UPDATED

6. Please merge the Acknowledgement section with the funding section and ensure the funding is also uploaded to our online eJP site.
UPDATED

7. Please remove the author contribution section from the main manuscript.
UPDATED

8. Please rename the conflict of interest section to Disclosure and Competing Interests section.
UPDATED

9. Please ensure that the references fit the EMBO style, no more than 10 author names listed before et al and remove DOIs.
UPDATED

10. We require the publication of source data, particularly for electrophoretic gels and blots and graphs, with the aim of making primary data more accessible and transparent to the reader. It would be great if you could provide me with a PDF file per figure that contains the original, uncropped and unprocessed scans of all or key gels used in the figure or for graphs, an Excel spreadsheet with the original data used to generate the graphs. The PDF files should be labeled with the appropriate figure/panel number, and should have molecular weight marker; further annotation could be useful but is not essential. The PDF files will be published online with the article as supplementary "Source Data" files.

UPDATED; completed per Source Data guidelines

11. Please provide a reagent table as described in our author guide.
UPDATED

12. We do not allow references to data not shown. Please remove this language from p21, p27, and p38.
UPDATED

13. We include a synopsis of the paper (see http://emboj.embopress.org/). Please provide me with a general summary statement and 3-5 bullet points that capture the key findings of the paper.
DONE

14. We also need a summary figure for the synopsis. The size should be 550 wide by 200-440 high (pixels). You can also use something from the figures if that is easier.
DONE

15. Please correct the section order to: Abstract, Keywords, Introduction, Results, Discussion, Methods, Acknowledgements, Disclosure and competing interests statement, References, Figure legends, Tables and their legends, Expanded View Figure legends.
UPDATED
16. Please note that the specific URLs for PXD053472 dataset is not provided in the data availability statement.
UPDATED

17. Please note that reviewer access code for PXD053472 dataset is not provided in the data availability statement.
UPDATED

18. Figure legends: Please note that the legend of figure 4g is mislabeled as figure 4d in the manuscript. This needs to be rectified.
UPDATED

19. Please note that the exact p values are not provided in the legends of figures 1c-e; 2e-g; 3a-k; 4d, g; 5g-h; 7b-f, h-i.
UPDTAED (Note: for multiple successive t-tests across time (e.g. Fig. 3I&J), it was not reasonable to include exact p-values for each time point.

20. Please indicate the statistical test used for data analysis in the legends of figures 1c-e; 2e-g; 3a-k; 4d, g; 5g-h; 7b-f, h-i.
UPDATED

21. Please note that information related to n is missing in the legends of figures 1c-e, g-k; 2e-g; 3a-k; 4a; 5a-b, g-h; 7b-c, e-f, h-i.
UPDATED

22. Please note that the error bars are not defined in the legends of figures 1c-e; 7b-f, h-i.
UPDATED

23. Please note that axis gap is not labeled appropriately in figure 1f.
UPDATED

Dear Bob,

Congratulations on an excellent manuscript, I am pleased to inform you that your manuscript has been accepted for publication in the EMBO Journal. Thank you for your comprehensive response to the referee concerns and for providing detailed source data. It has been a pleasure to work with you to get this to the acceptance stage.

I will begin the final checks on your manuscript before submitting to the publisher next week. Once at the publisher, it will take about 3 weeks for your manuscript to be published online. As a reminder, the entire review process, including referee concerns and your point-by-point response, will be available to readers.

I will be in touch throughout the final editorial process until publication. In the meantime, I hope you find time to celebrate!

Yours sincerely,
Kelly

Kelly M Anderson, PhD
Editor, The EMBO Journal
k.anderson@embojournal.org

Your manuscript will be processed for publication by EMBO Press. It will be copy edited and you will receive page proofs prior to publication. Please note that you will be contacted by Springer Nature Author Services to complete licensing and payment information.

You may qualify for financial assistance for your publication charges - either via a Springer Nature fully open access agreement or an EMBO initiative. Check your eligibility: https://www.embopress.org/page/journal/14602075/authorguide#chargesguide

Should you be planning a Press Release on your article, please get in contact with embo_production@springernature.com as early as possible in order to coordinate publication and release dates.

>>> Please note that it is The EMBO Journal policy for the transcript of the editorial process (containing referee reports and your response letter) to be published as an online supplement to each paper. If you do NOT want this, you will need to inform the Editorial Office via email immediately. More information is available here: https://www.embopress.org/transparent-process#Review_Process

** Click here to be directed to your login page: https://emboj.msubmit.net

EMBO Press Author Checklist

Corresponding Author Name: Robert T. Dirksen
Journal Submitted to: The EMBO Journal
Manuscript Number: EMBOJ-2024-116743R-Q

USEFUL LINKS FOR COMPLETING THIS FORM
The EMBO Journal - Author Guidelines
EMBO Reports - Author Guidelines
Molecular Systems Biology - Author Guidelines
EMBO Molecular Medicine - Author Guidelines

Reporting Checklist for Life Science Articles (updated January
This checklist is adapted from Materials Design Analysis Reporting (MDAR) Checklist for Authors. MDAR establishes a minimum set of requirements in transparent reporting in the life sciences (see Statement of Task: 10.31222/osf.io/9sm4x). Please follow the journal's guidelines in preparing your
Please note that a copy of this checklist will be published alongside your article.

Abridged guidelines for figures
1. Data
The data shown in figures should satisfy the following conditions:
- the data were obtained and processed according to the field's best practice and are presented to reflect the results of the experiments in an accurate and unbiased manner.
- ideally, figure panels should include only measurements that are directly comparable to each other and obtained with the same assay.
- plots include clearly labeled error bars for independent experiments and sample sizes. Unless justified, error bars should not be shown for technical
- if n<5, the individual data points from each experiment should be plotted. Any statistical test employed should be justified.
- Source Data should be included to report the data underlying figures according to the guidelines set out in the authorship guidelines on Data

2. Captions
Each figure caption should contain the following information, for each panel where they are relevant:
- a specification of the experimental system investigated (eg cell line, species name).
- the assay(s) and method(s) used to carry out the reported observations and measurements.
- an explicit mention of the biological and chemical entity(ies) that are being measured.
- an explicit mention of the biological and chemical entity(ies) that are altered/varied/perturbed in a controlled manner.
- the exact sample size (n) for each experimental group/condition, given as a number, not a range;
- a description of the sample collection allowing the reader to understand whether the samples represent technical or biological replicates (including how many animals, litters, cultures, etc.).
- a statement of how many times the experiment shown was independently replicated in the laboratory.
- definitions of statistical methods and measures:
  - common tests, such as t-test (please specify whether paired vs. unpaired), simple $\chi 2$ tests, Wilcoxon and Mann-Whitney tests, can be unambiguously identified by name only, but more complex techniques should be described in the methods section;
  - are tests one-sided or two-sided?
  - are there adjustments for multiple comparisons?
  - exact statistical test results, e.g., P values = x but not P values < x;
  - definition of 'center values' as median or average;
  - definition of error bars as s.d. or s.e.m.

Please complete ALL of the questions below.
Select "Not Applicable" only when the requested information is not relevant for your study.

Materials

Newly Created Materials	Information included in the manuscript?	In which section is the information available? (Reagents and Tools Table, Materials and Methods, Figures, Data Availability Section)
New materials and reagents need to be available; do any restrictions apply?	Yes	Material and Methods

Antibodies	Information included in the manuscript?	In which section is the information available? (Reagents and Tools Table, Materials and Methods, Figures, Data Availability Section)
For **antibodies** provide the following information:<br>- Commercial antibodies: RRID (if possible) or supplier name, catalogue number and or/clone number<br>- Non-commercial: RRID or citation	Yes	Reagent and Tools Table

DNA and RNA sequences	Information included in the manuscript?	In which section is the information available? (Reagents and Tools Table, Materials and Methods, Figures, Data Availability Section)
**Short novel DNA or RNA including primers, probes**: provide the sequences.	Yes	Material and Methods

Cell materials	Information included in the manuscript?	In which section is the information available? (Reagents and Tools Table, Materials and Methods, Figures, Data Availability Section)
**Cell lines:** Provide species information, strain. Provide accession number in repository **OR** supplier name, catalog number, clone number, and/**OR** RRID.	Yes	Reagent and Tools Table
**Primary cultures:** Provide species, strain, sex of origin, genetic modification status.	Yes	Reagent and Tools Table
Report if the cell lines were recently **authenticated** (e.g., by STR profiling) and tested for mycoplasma contamination.	Not Applicable	

Experimental animals	Information included in the manuscript?	In which section is the information available? (Reagents and Tools Table, Materials and Methods, Figures, Data Availability Section)
**Laboratory animals or Model organisms:** Provide species, strain, sex, age, genetic modification status. Provide accession number in repository **OR** supplier name, catalog number, clone number, **OR** RRID.	Yes	Reagent and Tools Table and Material and Methods
**Animal observed in or captured from the field:** Provide species, sex, and age where possible.	Not Applicable	
Please detail **housing and husbandry conditions**.	Yes	Material and Methods

Plants and microbes	Information included in the manuscript?	In which section is the information available? (Reagents and Tools Table, Materials and Methods, Figures, Data Availability Section)
**Plants:** provide species and strain, ecotype and cultivar where relevant, unique accession number if available, and source (including location for collected wild specimens).	Not Applicable	
**Microbes:** provide species and strain, unique accession number if available, and source.	Not Applicable	

Human research participants	Information included in the manuscript?	In which section is the information available? (Reagents and Tools Table, Materials and Methods, Figures, Data Availability Section)
If collected and within the bounds of privacy constraints report on age, sex and gender or ethnicity for all study participants.	Not Applicable	

Core facilities	Information included in the manuscript?	In which section is the information available? (Reagents and Tools Table, Materials and Methods, Figures, Data Availability Section)
If your work benefited from core facilities, was their service mentioned in the acknowledgments section?	Yes	Material and Methods and Acknowledgement

Design

Study protocol	Information included in the manuscript?	In which section is the information available? (Reagents and Tools Table, Materials and Methods, Figures, Data Availability Section)
If study protocol has been **pre-registered, provide DOI in the manuscript**. For clinical trials, provide the trial registration number **OR** cite DOI.	Not Applicable	
Report the **clinical trial registration number** (at ClinicalTrials.gov or equivalent), where applicable.	Not Applicable	

Laboratory protocol	Information included in the manuscript?	In which section is the information available? (Reagents and Tools Table, Materials and Methods, Figures, Data Availability Section)
Provide DOI OR other citation details if **external detailed step-by-step protocols** are available.	Yes	Material and Methods

Experimental study design and statistics	Information included in the manuscript?	In which section is the information available? (Reagents and Tools Table, Materials and Methods, Figures, Data Availability Section)
Include a statement about **sample size** estimate even if no statistical methods were used.	Yes	Figures
Were any steps taken to minimize the effects of subjective bias when allocating animals/samples to treatment (e.g. **randomization procedure**)? If yes, have they been described?	Yes	Material and Methods
Include a statement about **blinding** even if no blinding was done.	Yes	Material and Methods
Describe **inclusion/exclusion criteria** if samples or animals were excluded from the analysis. Were the criteria pre-established?<br><br>If sample or data points were omitted from analysis, report if this was due to attrition or intentional exclusion and provide justification.	Not Applicable	
For every figure, are **statistical tests** justified as appropriate? Do the data meet the assumptions of the tests (e.g., normal distribution)? Describe any methods used to assess it. Is there an estimate of variation within each group of data? Is the variance similar between the groups that are being statistically compared?	Yes	Material and Methods

Sample definition and in-laboratory replication	Information included in the manuscript?	In which section is the information available? (Reagents and Tools Table, Materials and Methods, Figures, Data Availability Section)
In the figure legends: state number of times the experiment was **replicated** in laboratory.	Yes	Figures
In the figure legends: define whether data describe **technical or biological replicates.**	Yes	Material and Methods

Ethics

Ethics	Information included in the manuscript?	In which section is the information available? (Reagents and Tools Table, Materials and Methods, Figures, Data Availability Section)
Studies involving **human participants**: State details of **authority granting ethics approval** (IRB or equivalent committee(s), provide reference number for approval.	Not Applicable	
Studies involving **human participants**: Include a statement confirming that **informed consent** was obtained from all subjects and that the experiments conformed to the principles set out in the WMA Declaration of Helsinki and the Department of Health and Human Services Belmont Report.	Not Applicable	
Studies involving **human participants:** For publication of **patient photos**, include a statement confirming that consent to publish was obtained.	Not Applicable	
Studies involving experimental **animals**: State details of **authority granting ethics approval** (IRB or equivalent committee(s), provide reference number for approval. Include a statement of compliance with ethical regulations.	Yes	Material and Methods
Studies involving **specimen and field samples:** State if relevant **permits** obtained, provide details of authority approving study; if none were required, explain why.	Not Applicable	

Dual Use Research of Concern (DURC)	Information included in the manuscript?	In which section is the information available? (Reagents and Tools Table, Materials and Methods, Figures, Data Availability Section)
Could your study fall under dual use research restrictions? Please check biosecurity documents and list of **select agents and toxins** (CDC): https://www.selectagents.gov/sat/list.htm	Not Applicable	
If you used a select agent, is the security level of the lab appropriate and reported in the manuscript?	Not Applicable	
If a study is subject to dual use research of concern regulations, is the name of the **authority granting approval and reference number** for the regulatory approval provided in the manuscript?	Not Applicable	

Reporting

The MDAR framework recommends adoption of discipline-specific guidelines, established and endorsed through community initiatives. Journals have their own policy about requiring specific guidelines and recommendations to complement MDAR.

Adherence to community standards	Information included in the manuscript?	In which section is the information available? (Reagents and Tools Table, Materials and Methods, Figures, Data Availability Section)
State if relevant guidelines or checklists (e.g., **ICMJE, MIBBI, ARRIVE, PRISMA**) have been followed or provided.	Not Applicable	
For **tumor marker prognostic studies,** we recommend that you follow the **REMARK** reporting guidelines (see link list at top right). See author guidelines, under 'Reporting Guidelines'. Please confirm you have followed these guidelines.	Not Applicable	
For **phase II and III randomized controlled trials**, please refer to the **CONSORT** flow diagram (see link list at top right) and submit the CONSORT checklist (see link list at top right) with your submission. See author guidelines, under 'Reporting Guidelines'. Please confirm you have submitted this list.	Not Applicable	

Data Availability

Data availability	Information included in the manuscript?	In which section is the information available? (Reagents and Tools Table, Materials and Methods, Figures, Data Availability Section)
Have **primary datasets** been deposited according to the journal's guidelines (see 'Data Deposition' section) and the respective accession numbers provided in the Data Availability Section?	Yes	Data avaliability
Were **human clinical and genomic datasets** deposited in a public access-controlled repository in accordance to ethical obligations to the patients and to the applicable consent agreement?	Not Applicable	
Are **computational models** that are central and integral to a study available without restrictions in a machine-readable form? Were the relevant accession numbers or links provided?	Not Applicable	
If publicly available data were reused, provide the respective **data citations in the reference list**.	Not Applicable	
